# Antimicrobial Use and Awareness of Antimicrobial Resistance in the Livestock Sector in the Western Balkans

**DOI:** 10.3390/antibiotics14080839

**Published:** 2025-08-19

**Authors:** Dora Kovacs, Eran Raizman, Anne Deckert, Natalia Ciria Artiga, Marijana Bošković, Ervin Bučan, Jelena Vračar Filipović, Olta Agolli, Dragana Grbić, Mevlida Hrapović, Ivan Ivanović, Nora Jusufi, Saša Lješković, Ljiljana Milovanović, Tamas Nagy, Miloš Palibrk, Milan Rogošić, Anna Sargsyan, Blagojcho Tabakovski, Daniel Beltran-Alcrudo

**Affiliations:** 1 Food and Agriculture Organization of the United Nations, Regional Office for Europe and Central Asia, 1054 Budapest, Hungary; eran.raizman@fao.org (E.R.); daniel.beltranalcrudo@fao.org (D.B.-A.); 2 Department of Population Medicine, Ontario Veterinary College, University of Guelph, Guelph, ON N1G 2W1, Canada; adeckert@uoguelph.ca; 3 Department of Animal Health and Anatomy, Autonomous University of Barcelona, 08193 Barcelona, Spain; natalia.ciria@uab.cat; 4 Administration of Food Safety, Veterinary and Phytosanitary Affairs of Montenegro, 81000 Podgorica, Montenegrojelena.vracar.filipovic@gmail.com (J.V.F.);; 5 Institute for Development Research and Alternatives, 1017 Tirana, Albania; olta.graca@idracompany.com (O.A.);; 6 Gyermelyi Holding Zrt., 2821 Gyermely, Hungary; 7 Strategic Development Agency, Yerevan 0070, Armenia

**Keywords:** antimicrobial resistance, AMR, antimicrobial use, AMU, Western Balkans, knowledge, attitudes and practices, KAP, livestock, farmers, veterinarians

## Abstract

**Background/Objectives:** Antimicrobial resistance (AMR) is a major threat to human, animal, and environmental health. To tackle AMR in the livestock sector, there is a need to understand the antimicrobial use (AMU) practices of different stakeholders in order to target the common knowledge gaps and inappropriate practices with tailored interventions. In the Western Balkans, published evidence shows the presence of AMR in both humans and animals. Since studies on AMU have mainly been conducted in humans, there is a significant knowledge gap about AMU in the livestock sector. The aim of this study was to assess the knowledge, attitudes, and practices of farmers, veterinarians, veterinary pharmacy personnel, and feed mill personnel related to AMU (focusing on antibiotics) and AMR in Albania, Bosnia and Herzegovina, Kosovo (References to Kosovo shall be understood to be in the context of Security Council resolution 1244 (1999)), Montenegro, North Macedonia, and Serbia. **Methods:** Field interviews were conducted in 2022 with 2815 participants in selected regions of the countries. **Results:** The findings showed that farmers engaged in imprudent practices, including purchasing antimicrobials without a prescription, administering antimicrobials for growth promotion, and disposing of expired antimicrobials in the garbage. Farmers’ main knowledge gaps were related to the duration of antimicrobial treatment and the differentiation between AMR and antimicrobial residues. This study also revealed poor record-keeping on animal treatments and a lack of some biosecurity measures. In terms of the attitudes and practices of veterinarians and veterinary pharmacy personnel, the belief that antimicrobial drugs are important for growth promotion, and the common use and sales of highest priority critically important antimicrobials should be targets for future interventions. **Conclusions:** Despite significant ongoing efforts to tackle AMR, there is still a need for training, awareness-raising, and policy interventions to address the knowledge gaps identified by this study and optimize AMU in the livestock sector in the Western Balkans.

## 1. Introduction

Bacterial antimicrobial resistance (AMR) is one of the main health threats of the 21st century [1]. It is estimated that globally, 1.91 million deaths could be attributable to, and 8.22 million deaths could be associated with AMR in 2050 [2]. The selection of resistant bacteria occurs with all antimicrobial use (AMU), but the overuse and misuse of these drugs are significant contributors to this phenomenon. Low-dose and prolonged use of antimicrobials, such as for growth promotion in food-producing animals, particularly favors the selection of resistant bacteria. These bacteria may then spread to humans via direct contact with infected livestock or indirectly through foods of animal origin, water, and animal waste application to farm fields [3]. Consequently, irresponsible and inappropriate AMU needs to be addressed in food-producing animals to prevent its negative impact on both animal and human health [4]. Interventions that reduce AMU in animals are associated with a decrease in the prevalence of AMR in both animals and humans [5]. To develop antimicrobial stewardship programs for the veterinary sector, first, there is a need to understand how antimicrobials are used in livestock [4].

According to the Global Database for Tracking AMR Country Self-Assessment Survey (TrACSS), countries in the Western Balkans have made significant progress toward tackling AMR in the animal health sector, yet there are still some gaps that need to be addressed. In Albania, since 2023, there has been sustained capacity for training and education on AMR in the veterinary sector and for AMR surveillance in terrestrial animals; however, the capacity for optimizing AMU in animal health remains limited. Bosnia and Herzegovina had no reported capacity for AMR surveillance in animals in 2024 and only limited capacity for veterinary education on AMR and for optimizing AMU in animal health. Montenegro, North Macedonia, and Serbia reported developed or demonstrated capacity by 2024 for veterinary education on AMR, optimizing AMU, and AMR surveillance in veterinary health, which must be sustained over time [6].

Currently, there is published evidence of AMU trends in humans from countries in the Western Balkans, while there is a limited number of studies published about AMU in animals. A recent study showed that—despite a decreasing trend between 2011 and 2019—the total antimicrobial consumption in Albania in the human health sector had increased in 2021 [7]. In addition, there was an increase observed in the use of Watch category antimicrobials [8] throughout the years, with macrolides, fluoroquinolones, and cephalosporins being among the most used antimicrobials in 2021 [7]. In Montenegro, a study on antimicrobial consumption between 2009 and 2015 showed that third-generation cephalosporins were the most commonly used antimicrobials in hospitals, followed by fluoroquinolones and aminoglycosides. This observation was linked to the finding that bacteria (*Klebsiella pneumoniae* and *Escherichia coli*) isolated from the blood of hospitalized patients between 2016 and 2018 showed high rates of resistance to third-generation cephalosporins, aminoglycosides, and aminopenicillins [9]. Ceftriaxone, a third-generation cephalosporin, was also reported to be the most commonly used antimicrobial in hospital settings in Kosovo (References to Kosovo shall be understood to be in the context of Security Council resolution 1244 (1999)) [10,11]. A study on the sales of antimicrobial veterinary medicinal products in Montenegro showed an increase from 2017 to 2019, followed by a decrease in 2021 [12]. However, the proportion of critically important antimicrobials [13] in overall sales increased between 2019 and 2021 [12]. The human use of Watch category antimicrobials [8] in Serbia increased between 2010 and 2019 [14]. According to a study conducted from 2006 to 2020, the use of ceftazidime, meropenem, and fluoroquinolones in humans also increased [15]. Additionally, the same study described an increase in ceftazidime, fluoroquinolone, and piperacillin/tazobactam resistance rates in *Pseudomonas aeruginosa* from 2013 to 2020 in Serbia [15].

Studies about AMR in animals and foods of animal origin from the Western Balkans also show high resistance rates and the widespread occurrence of multidrug-resistant (MDR) bacteria. In a study in Albania, *E. coli* and *Salmonella* spp. isolates from poultry farms showed high to extremely high levels of resistance to various antimicrobials, including fluoroquinolones, which are categorized as critically important [13]. Most isolates (95% of *E. coli* and 82% of *Salmonella* spp.) were determined as MDR [16]. In recent studies in Bosnia and Herzegovina, resistance to beta-lactam antimicrobials was commonly detected in *Staphylococcus* isolates from dogs [17] and in *E. coli* isolates, including extended-spectrum beta-lactamase (ESBL) producers, from poultry farms, retail supermarkets, butcheries, and slaughterhouses [18,19]. Staphylococcal isolates from cows with clinical mastitis in Kosovo also showed high rates of resistance to penicillin and ampicillin [20]. Several studies conducted in Serbia also demonstrated the presence of resistant bacteria, including MDR isolates, in poultry farms [21] and in various food samples (raw chicken meat, bulk tank milk, and meat carcasses) [22,23,24].

Considering the lack of data on AMU in the livestock sector in the Western Balkans and the need to understand AMU patterns to develop targeted antimicrobial stewardship programs, the aim of this study was to assess the knowledge, attitudes, and practices (KAP) of stakeholders in the livestock sector related to AMU and AMR in Albania, Bosnia and Herzegovina, Kosovo, Montenegro, North Macedonia, and Serbia. The ultimate goal was to use the findings of this study to provide an evidence base for targeted trainings, awareness-raising, and policy interventions that can be incorporated into the countries’ national action plans on AMR. This study was part of the Food and Agriculture Organization’s (FAO’s) mandate, aiming to support countries in tackling AMR in the food and agriculture sector. Antimicrobials covered by this study and discussed in this manuscript refer only to antibacterials.

## 2. Results

The following sections present the summary results for the Western Balkan region, i.e., aggregated data from Albania, Bosnia and Herzegovina, Kosovo, Montenegro, North Macedonia, and Serbia. For several of these countries, country-specific results are available in detailed country reports on FAO’s website (https://www.fao.org/antimicrobial-resistance/resources/publications-archive/en/ (accessed on 18 December 2024)) [25,26,27,28,29].

### 2.1. Demographics

A total of 2815 stakeholders were interviewed during this study, which included 2293 farmers, 274 veterinarians, 236 veterinary pharmacy personnel, and 12 feed mill personnel. The total number of interviews performed is shown in Table 1. In North Macedonia, the planned numbers for veterinarian and veterinary pharmacist interviews were 100 and 15, respectively, but in the end, only 13 veterinarians and 3 pharmacists responded to the questionnaires. For Serbia, 100 veterinarians were targeted with the surveys, but a large number of participants withdrew due to a lack of time or unforeseen emergencies. Lack of time was also a reason for many veterinarians and pharmacists from Albania refusing to participate in the surveys. It is important to note that farmers completed the species-specific survey for all the species that they raised for income. For example, a farmer who raised both chickens and pigs for sale would complete both the chicken and pig questionnaires. Therefore, the total number of species-specific surveys completed is greater than the number of farmers participating in the project.

With the exception of feed mill employees, the majority of respondents were male (78% of farmers, 91% of veterinarians, 89% of veterinary pharmacy personnel, and 33% of feed mill personnel). Farmers involved in the survey were predominantly the owners or managers (91%) of the farm, and most of them were either between the ages of 41 and 55 (37%) or over 55 years of age (43%). The median of farmers’ years of experience in livestock farming was 20 years; however, a remarkable proportion reported not having any previous education related to veterinary medicine, animal health, animal husbandry, pharmacology, or other agriculture-related areas (67%). Veterinarians, veterinary pharmacy personnel, and feed mill personnel were younger in general, with the majority being 25–40 or 41–55 years old (45% and 31% of veterinarians, 47% and 36% of veterinary pharmacy personnel, and 17% and 75% of feed mill personnel, respectively). The median years of experience of veterinarians and veterinary pharmacy personnel were 13 years for both target groups. None of the feed mill personnel reported preparing antimicrobial-containing feed for animals; therefore, the results of feed mill surveys are not detailed in the following sections.

### 2.2. Interviews with Farmers

#### 2.2.1. Practices

Record keeping regarding animal treatments was not common among the surveyed farmers. One third of participants kept records about veterinary visits (33%), and even fewer had records about animal medicines purchased (27%), animal treatments (28%), treatment protocols (19%), or prescriptions (21%). Vaccinations were recorded by 43% of farmers. More than one-third of farmers (42%) did not keep records about any of these data. Farmers generally had good biosecurity practices in place, including preventing wild animals from accessing the barns and feed storage, having rodent and pest control measures, and keeping sick animals separated. Chicken farmers also ensured good ventilation in the barns, removed litter and disinfected barns between batches of birds, kept litter in a relatively dry condition, and kept age groups separated. On dairy farms, good milking practices included using pre-milking teat dips, spritzing some milk from each teat before milking, and milking sick cows last. On the other hand, regardless of the species they reared, farmers did not routinely disinfect vehicles at the entrance of the farm, nor did they register visitors or provide protective clothing to them.

Overall, only 30% of farmers reported using antimicrobials in their animals (696 respondents); therefore, the results presented in this section consider only those individuals. Farmers mainly obtained antimicrobials from private veterinarians and veterinary pharmacies, with 67% and 65% of them reporting always or often purchasing from such sources, respectively. When buying antimicrobials, 17% of farmers never had a prescription, and 29% reported only rarely or occasionally having one. In most cases, the decision to use antimicrobials in farm animals was made by private veterinarians, but 43% of farmers also reported that the farm owner was always or often involved in the decision-making. When determining the dose and treatment duration of antimicrobials, farmers mainly requested advice from private veterinarians (83% always or often), but a considerable proportion of farmers also consulted a veterinary pharmacist (37% always or often) or relied on their own experience (37% always or often). Most farmers reported always following the dose (90%) and treatment duration (88%) recommended by the veterinarian when using antimicrobials, even though 54% believed that antimicrobial treatment could be stopped when the animal’s symptoms were improving (Table 2).

Many farmers reported throwing expired drugs into the garbage (42%), while others followed good practices such as consulting veterinarians (29%) or returning drugs to the place of purchase (11%).

#### 2.2.2. Knowledge and Attitudes

A total of 1488 farmers (65% of all those surveyed) reported knowing what antibiotics were. Among those who reported not knowing what they were, 10% (76 of 789 farmers) used these drugs in their animals. When the farmers—who reported knowing what antibiotics were—had to choose the definition of these drugs, 69% picked the correct one (“medicine that kills bacteria”) from a list provided. However, the majority believed that other interpretations were also correct, and only 23% chose the appropriate definition exclusively. The most frequently selected incorrect definitions included “medicine that kills disease” (chosen by 51%) and “medicine that kills germs” (chosen by 47%). More than half of the farmers (60%) reported having heard of AMR before the surveys.

The misconceptions of farmers who used antimicrobials are summarized in Table 2. The most common knowledge gaps were related to the importance of treatment length, the impact of AMU in animals on human health, and the difference between antimicrobial residues and resistance. On the other hand, farmers showed good understanding of the withdrawal period (i.e., that after each treatment, they need to wait a certain number of days before sending the animals for slaughter, or selling their eggs, milk or honey, based on the recommendations of the veterinarian or the pharmacist, or the user instructions of the veterinary medicine), and the importance of prevention and early detection of diseases for reducing AMU. Only 58% of farmers mentioned that they would be interested in learning more about antimicrobials.

### 2.3. Interviews with Veterinarians

Veterinarians generally had good record-keeping practices, with most of them keeping records of the names of antimicrobials sold or prescribed per year (89%), per farm visit (84%), or per farm (78%). Slightly fewer veterinarians recorded data on the volume of antimicrobials sold or prescribed per year (72%), per farm visit (78%), or per farm (63%). Only 6% reported not keeping any of the above-mentioned records about the antimicrobials they prescribed or sold.

Veterinarians most frequently obtained antimicrobials from veterinary pharmacies or wholesalers, with 77% and 74% always or usually purchasing from those sources, respectively. In case of sick animals, antimicrobial treatment was a first-choice measure for 12% of veterinarians, while 76% would first isolate the sick animals, and 10% would send samples for laboratory diagnostics first. When advising AMU to farmers, 82% of veterinarians always examined the animals, and 95% always informed the farmer about the withdrawal period. Almost all veterinarians (93%) reported having had information about AMR included in their veterinary training or education.

Between 78% and 92% of veterinarians would send samples to a diagnostic laboratory in case of infectious diseases on a farm, depending on whether it was a single case or an outbreak, and whether it was in a well-known or a newly visited herd. The highest number (92%) would do so in case of a disease outbreak in a well-known herd. Most veterinarians (87%) had good access to a veterinary diagnostic laboratory; however, they reported various issues that hindered using laboratory services, such as the clients not being willing to pay for testing (27%) or the testing taking too long (10%). In addition, 8% believed that laboratory testing was not necessary. Most veterinarians (82%) reported that antimicrobial susceptibility testing (AST) was available in the veterinary diagnostic laboratory that they worked with, and 99% stated that they would change antimicrobial treatment based on susceptibility results. On the other hand, slightly fewer veterinarians (90%) would consider previous AST results to choose antimicrobial treatments in the future.

### 2.4. Interviews with Veterinary Pharmacy Personnel

The majority of the surveyed veterinary pharmacy personnel (74%) had completed some sort of veterinary pharmacist training or education. Of those, 90% were taught about AMR during their training or education.

Overall, veterinary pharmacy personnel reported keeping thorough records about antimicrobial purchases. Almost all had records about the name and volume of each antimicrobial drug sold per purchase (88% and 85%, respectively) and per year (94% and 89%, respectively). Slightly fewer pharmacies kept detailed records about the volume per animal species (67%), the species and production stage of animals (76%), or the number of animals that antimicrobials were used in (70%). Only 4% of participants reported not keeping records about any of these data.

Veterinary pharmacies mainly obtained medications from veterinary wholesalers (90% always or usually purchased from them), but the market was also noted as a common source of drugs, with 49% of pharmacies always or usually buying there. Veterinary pharmacy customers were mainly the general public, including farmers, accounting for an average of 50% of all drug sales, followed by private and official veterinarians, with 28% and 18% respectively. When selling antimicrobials to farmers directly, only 58% of veterinary pharmacy personnel reported always requiring a prescription; 23% usually required one, but 12% rarely, and 8% never asked for a prescription. On the other hand, 95% of respondents informed customers about the withdrawal period when dispensing antimicrobials.

### 2.5. Most Used Antimicrobials and Common Antimicrobial Use Patterns

As there was a very low response rate by Serbian stakeholders to questions about the most used antimicrobials, the results described in this section show the drug use practices for Albania, Bosnia and Herzegovina, Kosovo, Montenegro, and North Macedonia. It is also noteworthy that not all farmers were aware of which antimicrobials were used in their animals, as treatment was the veterinarian’s responsibility. Among pharmacy personnel, 76% reported data about the most frequently sold antimicrobial-containing products; thus, the following section covers those responses as well. Stakeholders were asked to list the first, second, third, etc., most used/prescribed/sold antimicrobials in their practices (for farmers, veterinarians, and veterinary pharmacy personnel, respectively). The following section summarizes the drugs listed as the first, most used/prescribed/sold choices.

**Figure 1:**
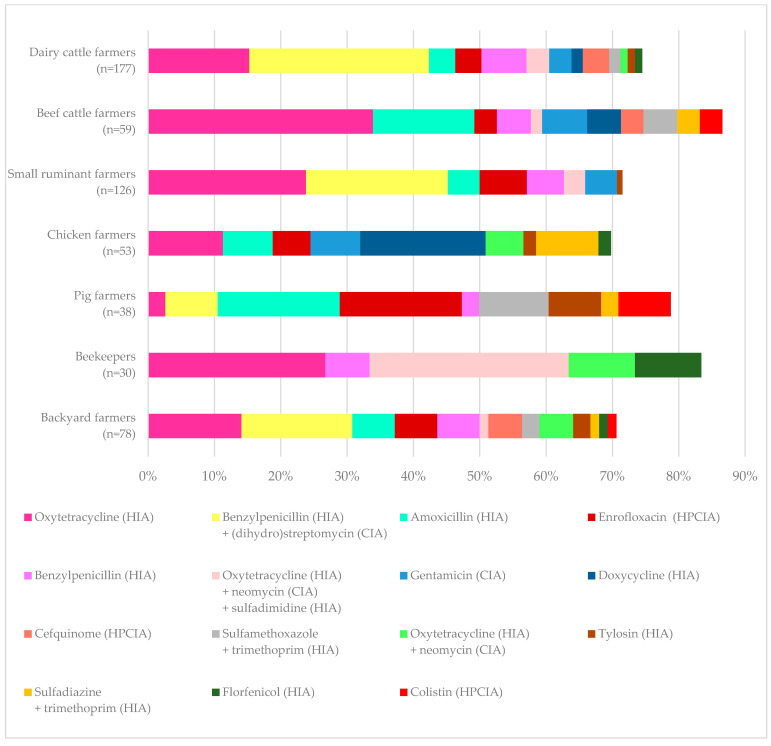
The top 15 substances reported by farmers as the first most used antimicrobials for different animal species and production systems. WHO Category: categorization of antimicrobials based on the World Health Organization (WHO) Medically Important Antimicrobials List for Human Medicine. HPCIA: highest priority critically important antimicrobials, CIA: critically important antimicrobials, HIA: highly important antimicrobials [13].

Farmers listed oxytetracycline, benzylpenicillin, in combination with streptomycin or dihydrostreptomycin, amoxicillin, and enrofloxacin as the first most used antimicrobials. Figure 1 shows the top 15 drugs and drug combinations reported as the first most used antimicrobials by farmers. Overall, oxytetracycline ranked the highest, but there were differences among animal species and production systems. The combination of benzylpenicillin with streptomycin or dihydrostreptomycin was the most used drug in dairy cattle, doxycycline in chickens, and amoxicillin and enrofloxacin in pigs.

**Figure 2:**
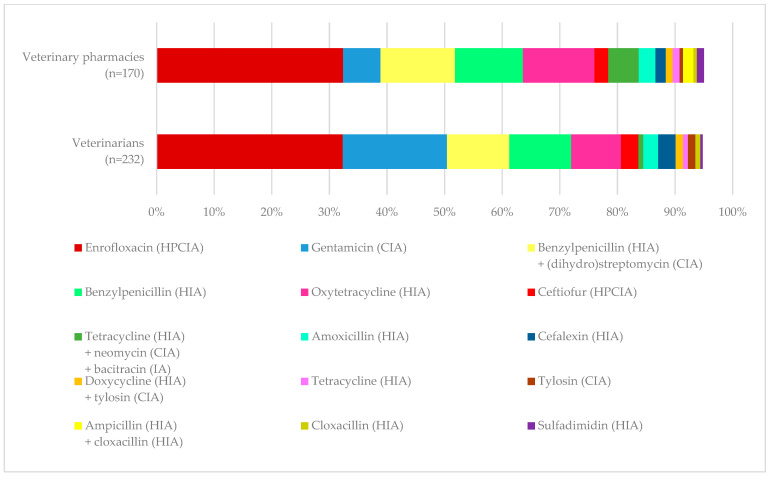
The top 15 substances reported by veterinarians and veterinary pharmacy personnel as the first most frequently prescribed or sold drugs. WHO Category: categorization of antimicrobials based on the World Health Organization Medically Important Antimicrobials List for Human Medicine. HPCIA: highest priority critically important antimicrobials, CIA: critically important antimicrobials, HIA: highly important antimicrobials, IA: important antimicrobials [13].

In line with farmers’ replies, veterinarians and veterinary pharmacy personnel listed enrofloxacin, oxytetracycline, and benzylpenicillin alone or in combination with streptomycin or dihydrostreptomycin among the first most used drugs. The use of enrofloxacin ranked highest among the responses of veterinarians and veterinary pharmacy personnel. Figure 2 shows the top 15 substances reported as the first most frequently prescribed or sold drug by veterinarians and veterinary pharmacy personnel. Although the majority were single drugs, some veterinarians (33%) reported combining individual antimicrobials to create new products when prescribing or selling to farmers.

Antimicrobials were used for the treatment of respiratory and gastrointestinal diseases in all animal species; for mastitis, metritis, and foot diseases in cattle and small ruminants; and for skin and feather problems in chickens. Typical administration routes for birds and mammals included parenteral, intramammary, topical, and oral (as medicated feed or medicated drinking water, bolus, or by drenching). For bees, drugs were administered in sugar water or powdered sugar. Farmers predominantly administered antimicrobials to adult animals. However, it was common to use antimicrobials in young animals among chicken farmers in Albania (in 54% of cases, antimicrobials were used in young animals), chicken (53%) and beef cattle (50%) farmers in Kosovo, and pig farmers in North Macedonia (65%). When an antimicrobial treatment did not yield the expected result, farmers generally consulted with a veterinarian for advice. Farmers in Bosnia and Herzegovina also reported repeating the treatment with the same drug, using another antimicrobial for treatment, or slaughtering the animals for meat.

Veterinarians were more likely to advise AMU for the treatment of diseases (93%) than for prevention (28%). The majority of them favored individual antimicrobial treatment, i.e., always treating the sick animals only (77%), while 13% reported always treating all animals in the pen, and 6% always treated all animals on the farm. Veterinarians also reported that the decision between individual and group treatment was dependent on the animal species to be treated (49% of respondents) and on the age of the animals to be treated (46% of respondents).

The use of antimicrobials for enhancing animal growth and production was reported by 67 farmers (10% of antimicrobial users, 3% of all farmers). Among veterinarians, 9% reported advising farmers to use antimicrobials for growth promotion, and 17% believed that AMU was important for this purpose. In addition, 8% of veterinary pharmacy personnel believed that antimicrobials were important for improving animal growth or production, and 8% thought that they were commonly used for this purpose.

A considerable proportion of respondents believed that the efficacy of antimicrobials had decreased. Among veterinarians and veterinary pharmacy personnel, 59% and 52% believed that antimicrobials were less effective than in the past, respectively, and 47% of farmers reported the same observation.

### 2.6. The Impact of the COVID-19 Pandemic

During or after the lockdowns due to the COVID-19 pandemic, the main difficulty reported by veterinarians was with accessing farms (37%). However, only 8% of farmers mentioned having problems with accessing veterinary support. A small proportion of all stakeholders reported having had problems with accessing disinfectants (17% of veterinarians, 9% of veterinary pharmacy personnel, and 7% of farmers), vaccines (12% of veterinarians, 6% of veterinary pharmacy personnel, and 3% of farmers), or antimicrobials (10% of veterinarians, 5% of veterinary pharmacy personnel, and 3% of farmers). An even smaller proportion of farmers (3%) and veterinarians (3%) had to increase the dose or frequency of antimicrobial treatments. In addition, 1% of pharmacy personnel were forced to sell expired antimicrobials, and 1% of farmers had to use expired antimicrobials.

## 3. Discussion

AMR is a major threat affecting all areas of health and the global economy. It must be tackled using a One Health approach, involving the coordination of numerous actors from the human and veterinary medicine, agriculture, and environment sectors [30]. In line with the recommendations of the WHO Global Action Plan on Antimicrobial Resistance [30] and the FAO Action Plan on Antimicrobial Resistance [31], significant progress is being made in the Western Balkans (Albania, Bosnia and Herzegovina, Kosovo, Montenegro, and North Macedonia) to optimize AMU in animals [6]. Nevertheless, the development, implementation, and monitoring of practices that can lead to reduced and optimized AMU and consequently decrease AMR prevalence require major behavioral changes that take time to become sustainable. This study provides insights into the knowledge, attitudes, and practices of stakeholders in the livestock sector related to AMU and AMR in the Western Balkans and complements previous research conducted in the human health field and among the general public in the region.

One of the key areas for improvement identified in this study is the sale of antimicrobials for animal use without a prescription, despite laws or regulations on prescription and sale of antimicrobials for terrestrial animal use already being in place in some countries at the time of the surveys [6]. This observation is in accordance with previous studies on AMU in humans in the Western Balkans, in which over-the-counter sales of antimicrobials were similarly described. In a study conducted in Albania in 2014–2015, pharmacists reported selling antimicrobials to patients without a prescription on a regular basis, mainly because of the pressure to satisfy their needs, and the fear that customers would, otherwise, turn to another pharmacy [32]. Similar findings were made in another Albanian study from 2014, when young adults reported using antimicrobials without a prescription, either obtained from a pharmacy or by taking leftover quantities from previous treatments or drugs provided by relatives or friends [33]. Similarly, buying antimicrobials in pharmacies without a prescription, taking non-prescribed antimicrobials, and insisting on an antimicrobial prescription against the advice of a doctor were also reported by the general public in Bosnia and Herzegovina, Kosovo, North Macedonia, and Serbia [10,34,35,36,37,38,39]. The consumption of non-prescribed antimicrobials may be based on the patient’s previous experience of taking the same drug or the advice of a colleague, a family member, or the pharmacist [36]. Studies from Bosnia and Herzegovina, Kosovo, and North Macedonia also revealed that many parents gave antimicrobials to their children without a paediatrician’s recommendation due to the following reasons: a lack of time to take the child to a medical examination, a lack of money to pay for the child’s treatment, having used the antimicrobial previously for the same symptom when prescribed by a paediatrician, or following the recommendation of a pharmacist, relatives, or friends [38,40,41]. These findings highlight the need to reinforce the monitoring of prescription use, the antimicrobial supply chain, and AMU, in both the human and animal population, as suggested by previous studies [7,32]. However, it should also be noted that the limited access farmers have to veterinary services may be among the reasons why they purchase antimicrobials without a prescription [E. Raizman, personal communication, 10 March 2025], which must be addressed to allow for better compliance with the national regulations on prescription use.

In terms of the knowledge and attitudes of farmers towards AMU, an important misconception in this study was related to the duration of antimicrobial treatment, i.e., many farmers (54% of those who reported using antimicrobials) believed that they could stop giving antimicrobials to an animal if the symptoms were improving. Similar observations were made in studies with humans from the Western Balkans. A 2014 study in Albania found that approximately one-third of young adults in Albania tended to stop taking antimicrobials when their symptoms disappeared [33]. Not following the doctor’s advice regarding the duration of antimicrobial treatment and discontinuing the treatment when symptoms were improving were also reported as common inadequate practices among the public in Bosnia and Herzegovina [34,35]. In Kosovo, around 40% of parents did not follow the paediatrician’s instructions completely regarding AMU in their children [41]. A study from North Macedonia showed inappropriate practices and misconceptions among parents, such as stopping antimicrobial treatment in their children when their signs were improving [38], and believing that taking less antimicrobials than prescribed does not reduce the effectiveness of the treatment but can be healthier than completing the full course [42]. A study conducted from 2015 to 2016 revealed that 43% of Serbian adults had stopped antimicrobial treatment during their most recent infection when their symptoms began improving [43].

Another knowledge gap observed in farmers was related to the definition of antibiotics: while many were aware that antibiotics act against bacteria, not all could distinguish them from other microorganisms (i.e., believing that antibiotics are “medicine that kills disease” or “medicine that kills germs”). Similar to this observation, a study on the knowledge, attitudes, and behaviors of different stakeholders in Albania revealed that human patients recognized antibiotics as effective in treating infections, but only a few participants differentiated between bacterial and viral infections [32]. Similarly, not all pharmacists interviewed in an Albanian study were aware that antibiotics are not effective against viruses, fungi, and parasites [44]. In Kosovo, only 35% and 26% of citizens were aware that antibiotics are not effective against viruses and colds, respectively [37]. Misconceptions of parents from Tetovo, North Macedonia, included the belief that antibiotics were effective against viruses and colds in their children [42]. Similarly, in a study from 2022, Serbian adults erroneously believed that antibiotics were effective against viruses (59%) and colds (52%) [39]. Studies from the European Union have also shown similar misconceptions among farmers. In Scottish dairy farms, farmers were aware that antibiotics are effective against bacteria, but many of them believed that these drugs also acted against parasites, viruses, and had an anti-inflammatory and/or analgesic effect [4].

The fact that many farmers in the current study believed that resistance occurred when antimicrobials were found in the meat or milk of an animal is also noteworthy. By confusing antimicrobial residues with resistance, farmers may believe that by following the withdrawal period, they eliminate all human health risks arising from AMU in animals, overlooking the possibility of AMR development and spread. With regards to the respondents’ knowledge of the withdrawal period, almost all veterinarians (95%) and veterinary pharmacy personnel (95%) reported informing farmers about the withdrawal period when advising or selling antimicrobials, and it was confirmed by the finding that most farmers (88%) were aware of the concept. However, following the withdrawal period correctly may be challenging in the absence of thorough treatment records, which was observed on many farms.

Another potential area for intervention, based on results from this study, is to target the common use of enrofloxacin (fluoroquinolone antimicrobial), which is recommended to be used based on AST results whenever possible and in situations when less valuable antimicrobials may not be clinically effective [45]. Additionally, the use of other highest priority critically important antimicrobials [13] (colistin, ceftiofur, and cefquinome) was also reported by participants, although less commonly than enrofloxacin. In principle, antimicrobials with a broader spectrum of activity have a stronger impact on the commensal microbiota and the selection of resistance. The use of fluoroquinolones and third- and fourth-generation cephalosporins is associated with the selection of MDR bacteria of animal and public health importance, such as methicillin-resistant staphylococci and ESBL-producing *Enterobacteriaceae*. Therefore, the use of such substances should be reserved for difficult-to-treat infections and should not be used as first-line treatment unless specifically recommended for a certain condition by expert guidelines [46]. Nevertheless, there are factors that may contribute to the overuse of broad-spectrum antimicrobials. A study from Albania with healthcare professionals suggested that the increased use of Watch antimicrobials may be explained by physicians feeling safer when prescribing these broad-spectrum drugs in the absence of AST data [7]. This reasoning may be applicable to veterinarians as well. A study in healthcare settings in Kosovo reported that the main reasons behind the overuse of broad-spectrum antimicrobials were the lack of protocols, AST, prescription monitoring, and stewardship programs [10]. The use of some long-acting formulations of extended-spectrum cephalosporins may also be favored due to the convenience of administration and the short withdrawal periods [47]. It is noteworthy that in the current study, information provided by Serbian respondents on the most used antimicrobials was scarce. Nevertheless, a recent survey among Serbian veterinarians indicated that enrofloxacin was one of the most commonly prescribed drugs in their practice [48], which is in accordance with our findings from the other Western Balkan countries.

In this study, around half of the respondents reported observing a decrease in the efficacy of antimicrobials, which suggests the widespread prevalence of resistant bacteria in the livestock sector and underscores the importance of interventions against AMR. Similar to our findings among livestock sector stakeholders, most of the human healthcare professionals surveyed in Albania acknowledged the existence of AMR, and many of them experienced treatment failure due to it [32]. In a study from 2021 with Serbian farm animal veterinarians, almost all respondents (98%) experienced AMR in their work at least occasionally, and 93% acknowledged AMR as an emerging problem for human and animal health [49].

In this study, 10% of antimicrobial user farmers reported administering antimicrobials for promoting animal growth or production, and 9% of veterinarians would also advise AMU for such purposes. The use of antimicrobials for promoting growth is an important risk factor for the selection of resistant bacteria [3], and should, therefore, be eliminated with targeted interventions, i.e., prohibiting this type of use, educating farmers, improving management and nutrition, and promoting the use of alternatives to antibiotics. Since veterinarians are the main source of advice for farmers, they must be cautious and use their influence to improve AMU practices in livestock production. It is also necessary to enhance veterinarians’ education related to prudent AMU and AMR and provide them with skills to engage farmers in actions against AMR. Farmers may have financial, time, and workforce limitations that hamper the implementation of better disease prevention practices (e.g., optimal housing and hygiene) that could lead to decreased AMU. Promoting the effectiveness of low-cost measures, as well as economic incentives for low antimicrobial users, can encourage farmers to optimize their AMU practices [4]. According to the findings of this study, improving farmers’ record-keeping and biosecurity practices could be potential targets for such interventions in the Western Balkans.

Awareness-raising and training on prudent AMU and AMR can also be beneficial for farmers [4,50]. Future activities should focus on the main knowledge gaps and misconceptions identified in this study, such as the spectrum of activity of antimicrobials, the importance of treatment duration, the difference between antimicrobial residues and resistance, and the handling of expired antimicrobials. Unfortunately, it is noteworthy that in this study, only 58% of farmers were interested in learning more about antimicrobials. Any training and awareness-raising that targets these stakeholders should, therefore, emphasize the health and economic benefits of prudent AMU practices.

Veterinarians may occasionally feel pressure when farmers expect them to prescribe antimicrobials even if it is not clinically necessary [49,51,52], particularly if they have less experience [51]. The training of veterinarians to act as confident and independent advisors can be beneficial in resisting such pressure and in finding alternative solutions to AMU [51]. Additional factors that may influence the prescribing habits of veterinarians include the cost of diagnostic tests or a shortage of their availability, a lack of guidelines, and insufficient education [49]. In this study, some veterinarians also highlighted that laboratory testing was too time-consuming, and others believed that testing was not necessary. Some of these factors could be improved with targeted training for veterinarians and the development of national treatment and diagnostic guidelines for priority bacterial diseases. Treatment guidelines may also be beneficial in reducing the unnecessary use of critically important antimicrobials.

One of the strengths of the present study was the large sample size across six countries in the region. Efforts were made to obtain a representative sample from each stakeholder group and country; however, the target sample size could not always be reached due to professionals refusing to participate for various reasons. Limitations of this study include the potential of social desirability bias, i.e., respondents giving socially accepted answers instead of the truth. By guaranteeing anonymity, we aimed to minimize the risk of this bias. An additional limitation, due to the information in this survey being self-reported, is recall bias. Finally, a small number of respondents refused to answer some questions in the survey, with the most common reasons being concerns that their data would be shared with researchers, the government, or other farmers. This led to some incomplete questionnaires not being excluded from the data analysis in order to capture other relevant information.

## 4. Materials and Methods

Ethics approval for this study was granted by the Research Ethics Boards (REB) of the University of Guelph, Canada (approval number: 20-05-010). The KAP surveys and associated materials, such as field instructions and informed consent forms, were developed by an expert team at FAO. The implementation was carried out in collaboration with national authorities, non-governmental organizations, and national subject matter experts. The interviews were conducted by the Institute for Development Research and Alternatives (IDRA) in Albania and Kosovo; the Association to Combat Land Degradation and Environment Protection “Mother Nature” in Bosnia and Herzegovina; the Administration of Food Safety, Veterinary and Phytosanitary Affairs (AFSVPA) in Montenegro; the Veterinary Chamber of the Republic of North Macedonia (VC) and Blagojcho Tabakovski (FAO) in North Macedonia; and the Regional Development Agency Zlatibor in Serbia. The surveys targeted the main stakeholders in the livestock sector, including farmers of priority livestock species, field veterinarians, veterinary pharmacy personnel, and feed mill personnel, focusing on the main production systems and stakeholders in each country.

### 4.1. Survey Design

A specific set of questionnaires was developed for each target stakeholder group (Supplementary Material Document S1). For farmers, the survey was divided into two parts: a general survey, which had to be completed by all respondents, followed by species-specific questionnaires that were chosen and completed based on the animal species housed on the farm. Species-specific questionnaires were used for all species that were raised for sale to provide income for the farmers. If the animals were raised primarily for the consumption of the household, the backyard questionnaire was used.

The first section of each survey gathered demographic and production information about the participants and their farm, veterinary practice, pharmacy, or feed mill (e.g., age, gender, years of experience, education, farm size and animal species housed, number and type of farms visited by the veterinarian), and the animal diseases that they most frequently encountered. The questionnaires also assessed the likelihood of respondents following best practices related to record keeping, disease management, laboratory diagnostics, hygiene, and biosecurity.

For farmers, questions explored their awareness and understanding of antimicrobials, AMR, and the principles of prudent AMU. Those farmers who reported using antimicrobials were asked to indicate whether they agreed or disagreed with statements that explored their knowledge about the effects of antimicrobials and various aspects of AMU. Veterinarians and veterinary pharmacies were asked to indicate the perceived importance and common use of antimicrobials for various purposes. Respondents were also asked about their experience with the efficacy of antimicrobials.

The surveys investigated the general AMU practices of respondents, covering the main sources of antimicrobials, the use of prescriptions, decision-making on AMU, handling of expired antimicrobials, the use of antimicrobials for growth promotion, observation of the withdrawal period, and AMU for individual or group treatment. In addition, the last section in each survey explored the antimicrobials most commonly used/prescribed/sold/mixed in feed (for farmers, veterinarians, pharmacies, and feed mills, respectively), as well as the indication and details of their use (e.g., treatment or prevention, the age group and proportion of animals medicated, administration route, treatment length, and actions in case of treatment failure).

Respondents were also asked to identify any difficulty they experienced resulting from the impacts of the COVID-19 pandemic, such as the increased incidence of animal diseases or mortality; difficulties with veterinarians accessing farms; shortages in antimicrobials, vaccines, or disinfectants; and the need to use antimicrobials more commonly or at a higher dose.

To facilitate the completion of the last section covering the most used, prescribed, or sold antimicrobials, a coded list of commercially available antimicrobial products was prepared for each country, including pictures of the veterinary medicinal products, their trade name, and the active substance(s) included. Once respondents picked the most commonly used or sold drugs from the list, the respective codes were entered into the survey platform and were then decoded for the active substance(s) during data analysis.

The surveys consisted of multiple-choice, dichotomous, matrix, and rank-order questions. Free text fields were also provided when participants had the option to reply “Other”, in order to explain their answer if none of the pre-defined options were appropriate. Some questions were followed by sub-questions depending on the answer selected (e.g., the questions assessing AMU practices of farmers were only completed if the participants reported using antimicrobials in their animals). For some questions, the respondents could opt for one or more answers (e.g., when choosing the definition of antibiotics) or could give the same rating for multiple options (e.g., consulting both the veterinarian and the veterinary pharmacists for advice on AMU with equal likelihood). Quantitative data on AMU was not collected.

### 4.2. Selection of Respondents

In each country, surveys were conducted in selected regions, between 2 June and 22 November 2022. The regions covered in this study are shown in Supplementary Material Document S2. The selection of target production systems and the number of interviews per region and per stakeholder were determined based on animal inventory data and information provided by national authorities and local experts. In some cases, when the pre-defined targets could not be reached for one stakeholder type or region due to herd migration or refusal to participate, the quotas were redistributed to other stakeholders or regions to achieve the desired national total.

As there were no stationary veterinary pharmacies in Kosovo, the surveys developed for pharmacies were administered to the equivalent registered veterinary ambulances that sold veterinary drugs. Throughout this manuscript, references made to veterinary pharmacies or veterinary pharmacy personnel cover veterinary ambulances in the case of Kosovo.

The questionnaires were administered to personnel who had knowledge about AMU practices and policies at the farm, pharmacy, or feed mill. Veterinarians were selected on the basis of providing direct services to farmers for the selected priority livestock species.

### 4.3. Interview Process

The questionnaires were developed in English and were then translated into the local language(s) of each country to ensure good understanding by participants. Local enumerators were trained on survey implementation and on AMU and AMR before implementation. Pre-testing in field conditions was performed to ensure question clarity during the interviews. All interviews were performed in person, using tablets to record responses. The exceptions were surveys with veterinarians and veterinary pharmacies in North Macedonia, which were conducted online, with the survey links and instructions being sent via email to private veterinary practices and legal entities selling veterinary medicinal products. All survey data was collected in the KoboCollect platform, which was managed by the Strategic Development Agency (SDA) in Armenia.

All participants were required to provide written informed consent before starting the interviews and were assured that their responses would be kept anonymous and their personal information confidential. Participants were allowed to skip questions; nevertheless, they were encouraged to provide answers to all questions. At the end of the surveys, an information leaflet (https://openknowledge.fao.org/handle/20.500.14283/cb1585en (accessed on 18 December 2024)) was provided to participants, which outlined the basic concepts of AMR and prudent AMU in livestock, and the enumerators provided explanations and clarifications related to misconceptions that became apparent during the interviews.

### 4.4. Data Analysis

Data were downloaded from KoboCollect in Microsoft Excel format and were cleaned to remove potential duplicates and errors. Incomplete surveys were not removed from the datasets and were included in this analysis. A descriptive analysis of responses was conducted for each question in Microsoft Excel Version 2506 and R Core Team 4.2.1. (2022).

## 5. Conclusions

To the best of our knowledge, this is the first large-scale survey that describes the knowledge, attitudes, and practices of stakeholders in the livestock sector related to AMU and AMR in the Western Balkans. Despite significant efforts in these countries toward antimicrobial stewardship, this study shows that stakeholders in the livestock sector have misconceptions regarding AMU and AMR and engage in a variety of imprudent practices. The key findings of this study were presented and discussed with national AMR focal points during a regional workshop in Budapest in 2023, and specific follow-up actions were identified to address the areas requiring attention to reduce the development and spread of AMR in the livestock sector. Detailed reports about the study findings and recommendations were shared with the participating countries to be used as a basis for developing and implementing trainings, awareness-raising campaigns, and policy interventions targeted at the main knowledge gaps and most common inappropriate practices identified. Country reports for several countries involved in this survey are available on FAO’s website [25,26,27,28,29].

## Figures and Tables

**Table 1:** Number of interviews performed.

Country	Regions Covered in This Study	Total # Participants	Interviews Performed										
			F	BC	DC	SR	P	C	B	BY	V	VP	FM
Albania	Berat, Dibra, Durrës, Elbasan, Fier, Gjirokastër, Korçë, Kukës, Lezhë, Shkodër, Tirana, Vlorë counties	575	525	*18*	*145*	*156*	-	*93*	*64*	*86*	29	21	-
Bosnia and Herzegovina	Federation of Bosnia and Herzegovina, Republic of Srpska	483	275	*108*	*102*	*30*	*23*	*21*	*46*	*208*	100	100	8
Kosovo	Ferizaj, Gjakova, Gjilan, Mitrovica, Peja, Pristina, Prizren districts	482	352	*27*	*77*	*49*	-	*69*	*66*	*84*	65	65	-
Montenegro	Central, Coastal, North regions	454	359	-	*96*	*79*	*22*	*54*	*56*	*108*	55	40	-
North Macedonia	Pelagonia, Polog, Skopje, Vardar, Eastern, North- eastern, Southeastern, Southwestern regions	546	530	*82*	*97*	*116*	*53*	*23*	*98*	*55*	13	3	-
Serbia	Belgrade, Sumadija and Western Serbia, Southern and Eastern Serbia, Vojvodina statistical regions	275	252	*52*	*76*	*80*	*90*	*68*	*46*	*77*	12	7	4
Total		2815	2293	*287*	*593*	*510*	*188*	*328*	*376*	*618*	274	236	12

F: Farmers, BC: Beef cattle section, DC: Dairy cattle section, SR: Small ruminant section, P: Pig section, C: Chicken section, B: Bee section, BY: Backyard section, V: Veterinarians, VP: Veterinary pharmacy personnel (in Kosovo: veterinary ambulance personnel), FM: Feed mill personnel. Numbers in italics indicate the species-specific surveys completed by farmers. The total number of species-specific surveys completed is greater than the number of farmers that participated (column F), as they were requested to complete a separate survey for each species that they raised for income.

**Table 2:** Farmers’ beliefs about antimicrobials and antimicrobial use.

Statements on Antimicrobials and Antimicrobial Use	Farmers’ Replies			# Replies
	Agree	Disagree	Do Not know	
You can stop giving antibiotics to an animal if their symptoms are improving. (correct: disagree)	**54%**	*37%*	9%	691
If antibiotics are given too often, they might stop working. (correct: agree)	*69%*	**13%**	18%	695
Giving antibiotics to healthy animals will prevent them from becoming sick in the future. (correct: disagree)	**24%**	*54%*	21%	692
Using vaccines can prevent the use of antibiotics. (correct: agree)	*57%*	**18%**	24%	692
Animals can transmit disease to humans. (correct: agree)	*71%*	**16%**	13%	694
Antibiotic use in animals does not affect human health. (correct: disagree)	**30%**	*50%*	20%	695
Antibiotics may be freely discarded without having any action/effect on the environment. (correct: disagree)	**8%**	*68%*	24%	692
Antibiotic resistance occurs when antibiotics are found in the meat or milk of an animal. (correct: disagree)	**49%**	*12%*	39%	687
When you use antibiotics, there is a certain number of days you should wait before selling the animals for slaughter, selling eggs, milk, or honey. (correct: agree)	*88%*	**3%**	9%	694

Correct answers are shown in italics, and misconceptions are shown in bold.

## Data Availability

The original contributions presented in this study are included in the article/Supplementary Material. Further inquiries can be directed to the corresponding author(s).

## Supplementary Materials

The following supporting information can be downloaded at https://www.mdpi.com/article/10.3390/antibiotics14080839/s1, Document S1: Survey instructions and questionnaires_Western Balkans; Document S2: Regions covered in this study with maps. More details of the survey process and the country-specific results are available in the detailed country reports on FAO’s website [25,26,27,28,29].

## Abbreviations

The following abbreviations are used in this manuscript:

AMR	Antimicrobial Resistance
AMU	Antimicrobial Use
AST	Antimicrobial Susceptibility Testing
CIA	Critically Important Antimicrobials
ESBL	Extended-Spectrum Beta-lactamase
FAO	Food and Agriculture Organization
HIA	Highly Important Antimicrobials
HPCIA	Highest Priority Critically Important Antimicrobials
IA	Important Antimicrobials
KAP	Knowledge, Attitudes, and Practices
MDR	Multidrug-resistant
TrACSS	Tracking AMR Country Self-Assessment Survey
WHO	World Health Organization
