# Supplementary material for: Antimicrobial Use and Awareness of Antimicrobial Resistance in the Livestock Sector in the Western Balkans
Source: Antibiotics (Basel). 2025 Aug 19;14(8):839. doi: 10.3390/antibiotics14080839 (PMC12382872; doi:10.3390/antibiotics14080839)
Supplement: Supplementary file 1 [file antibiotics-14-00839-s001.zip › Regions covered in the study with maps.pdf]

Regions covered by the study

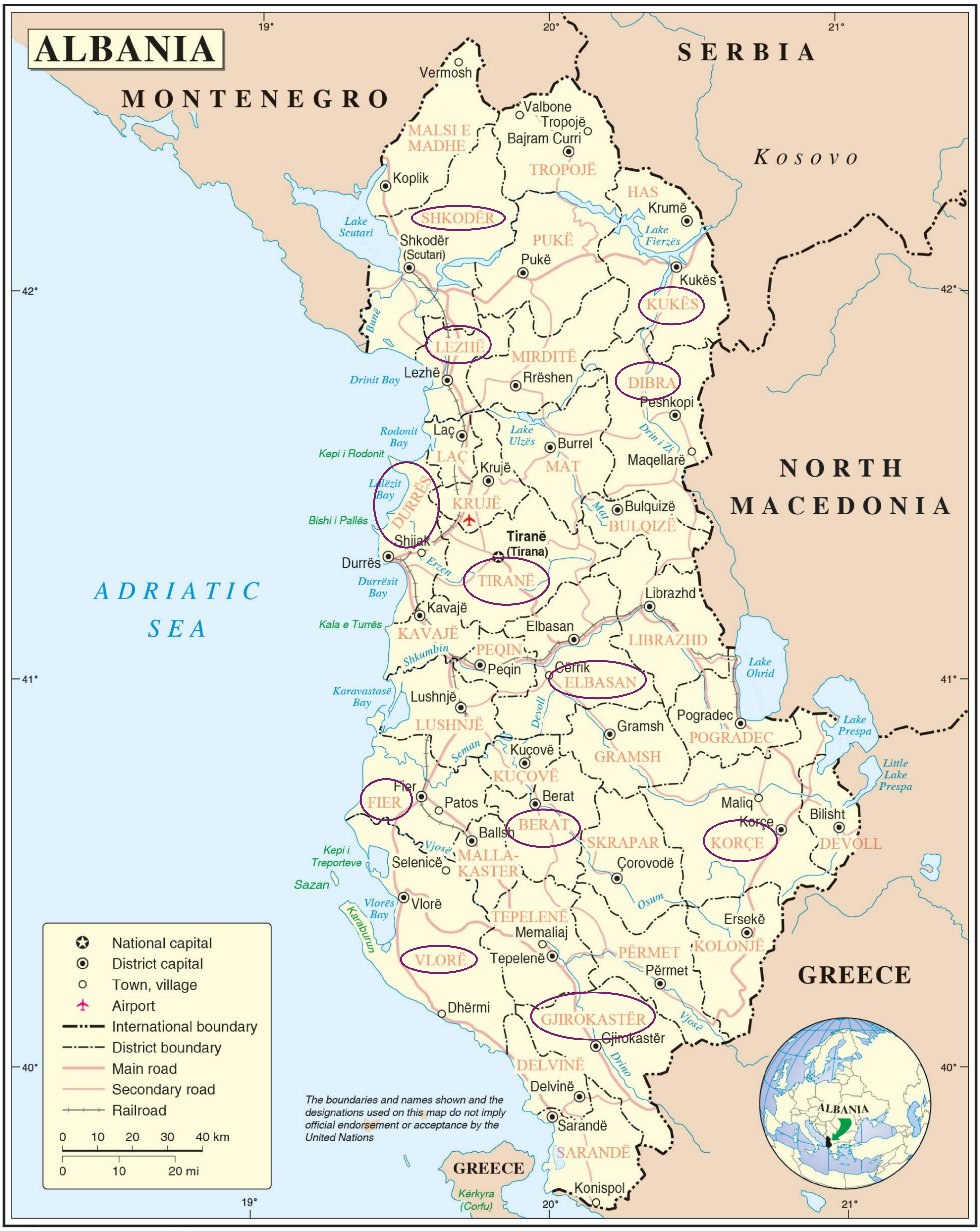

Map No. 3769 Rev. 8 UNITED NATIONS
August 2020

Office of Information and Communications Technology
Geospatial Information Section

Albania: Berat, Dibra, Durrës, Elbasan, Fier, Gjirokastër, Korçë, Kukës, Lezhë, Shkodër, Tirana, Vlorë counties

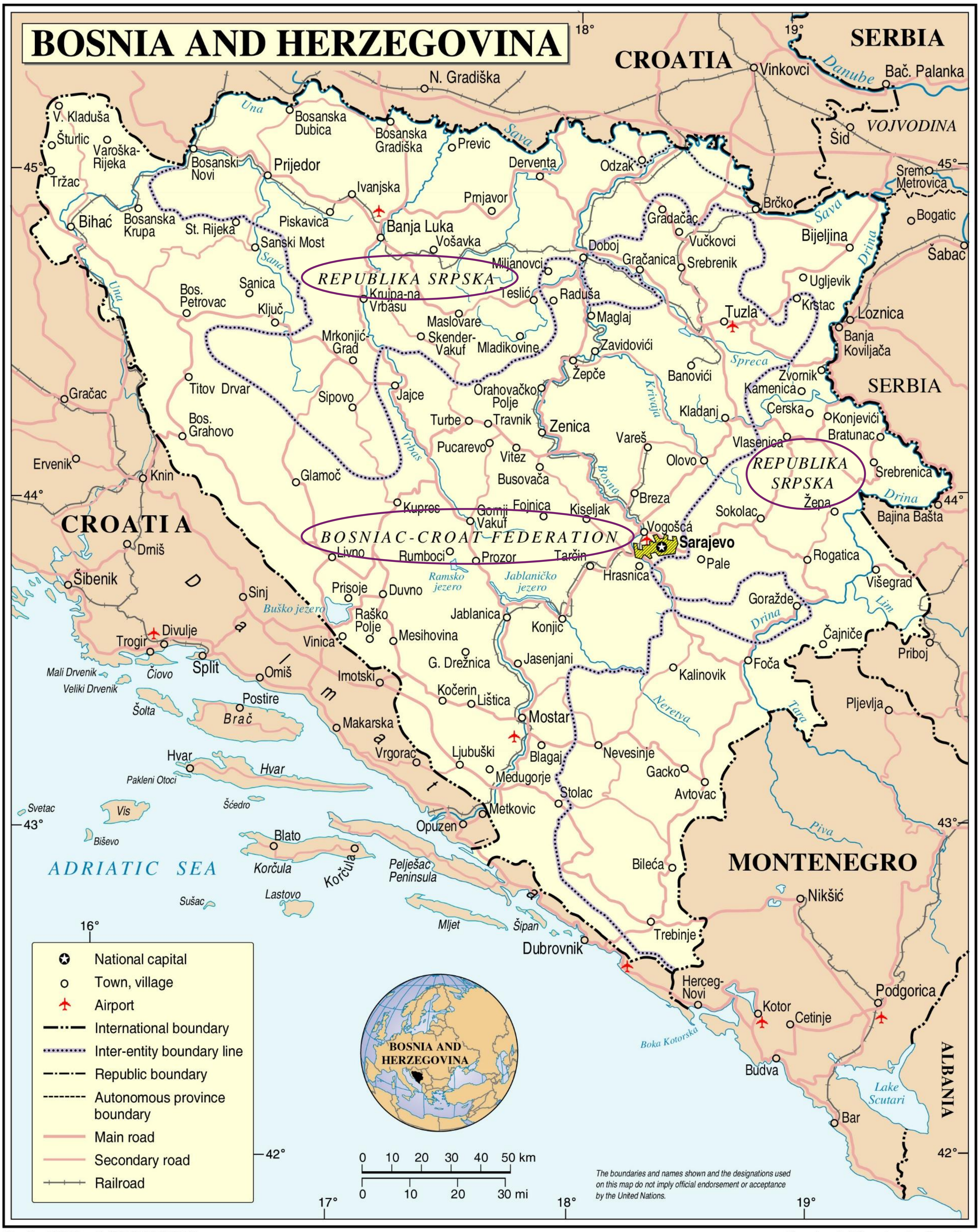

Map No. 3729 Rev. 6 UNITED NATIONS
March 2007

Department of Peacekeeping Operations
Cartographic Section

Bosnia and Herzegovina: Federation of Bosnia and Herzegovina, Republic of Srpska

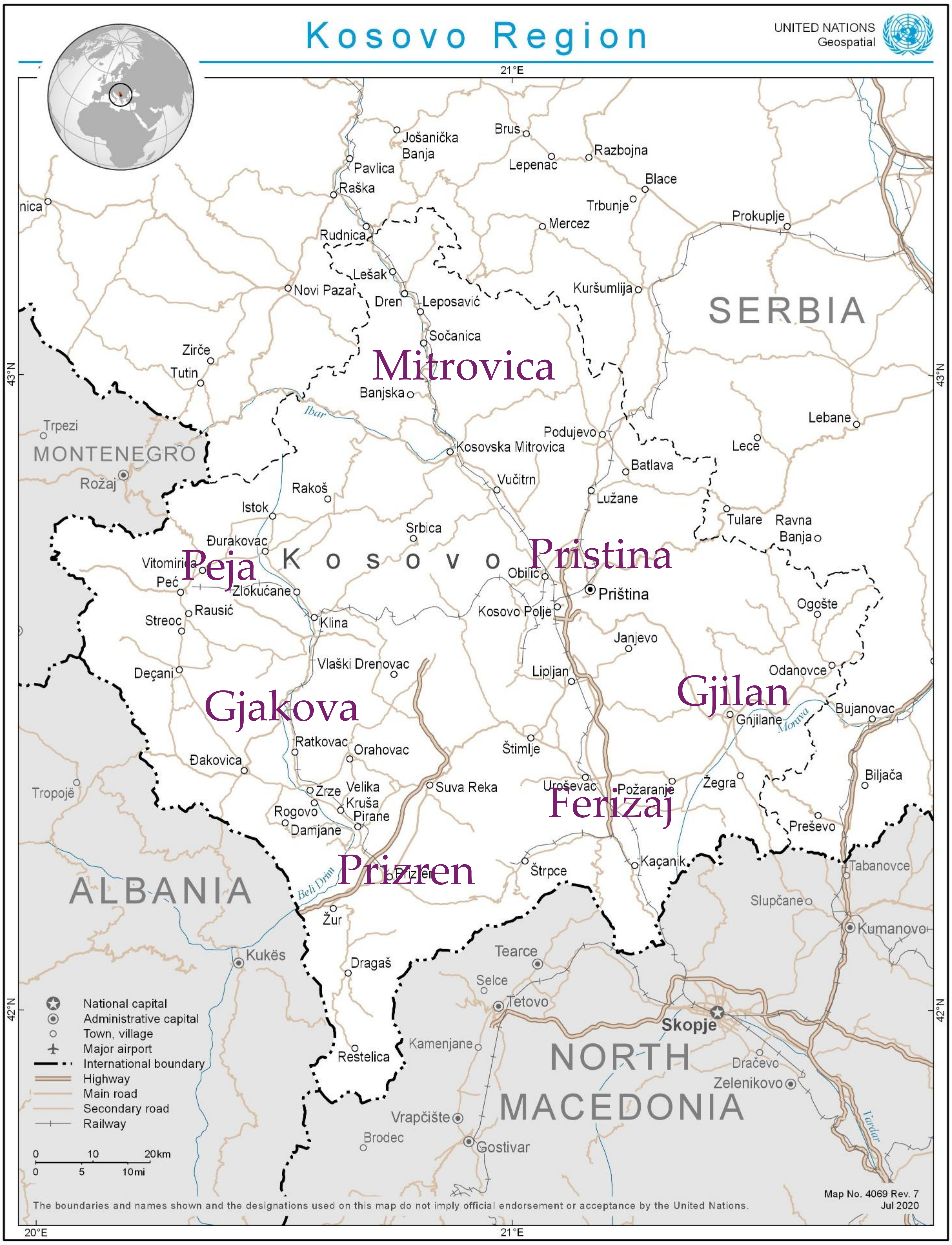

Kosovo: Ferizaj, Gjakova, Gjilan, Mitrovica, Peja, Pristina, Prizren districts

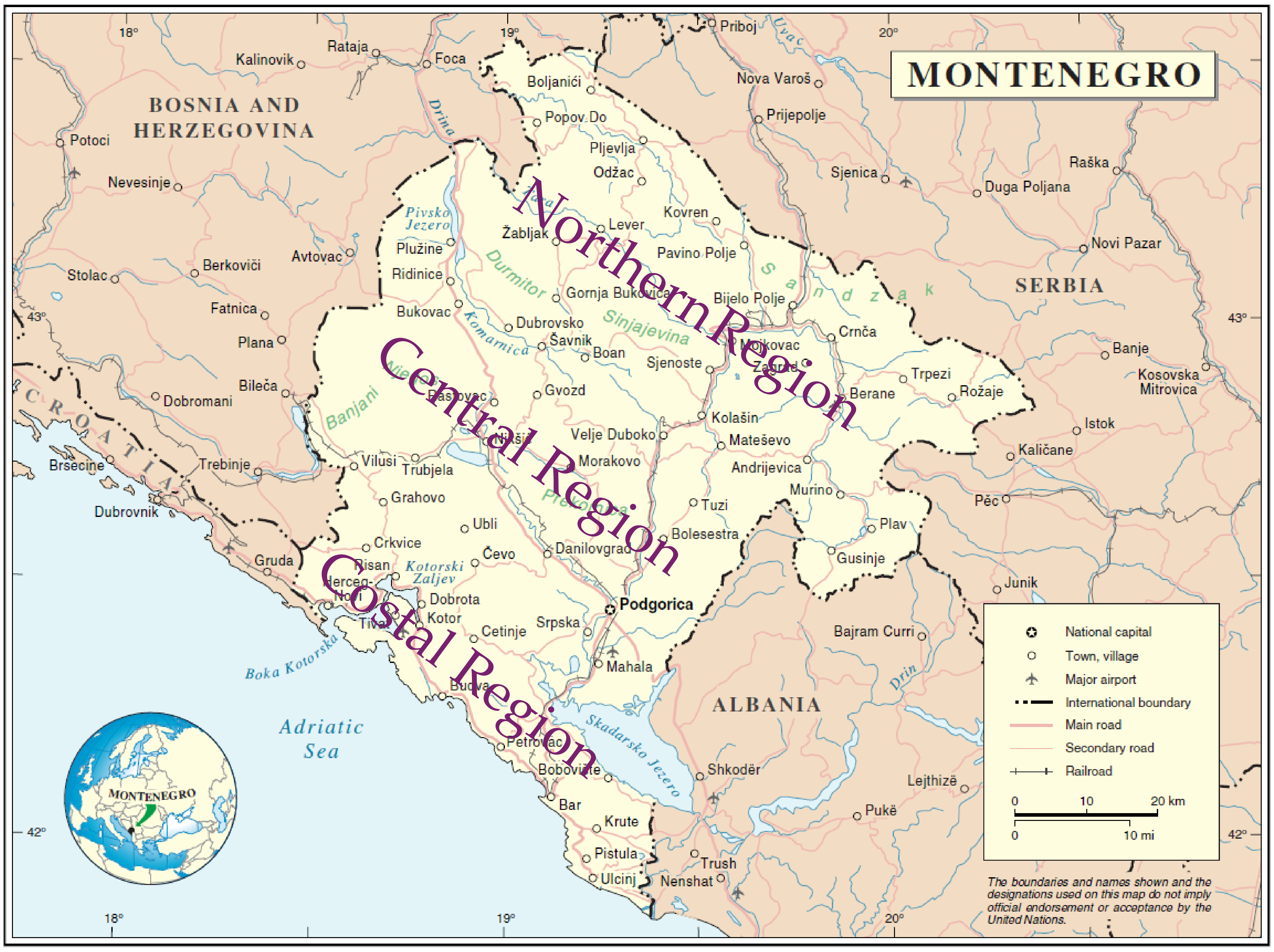

Map No. 4274 UNITED NATIONS
July 2006 (Colour)

Department of Peacekeeping Operations
Cartographic Section

Montenegro: Central, Coastal, North regions

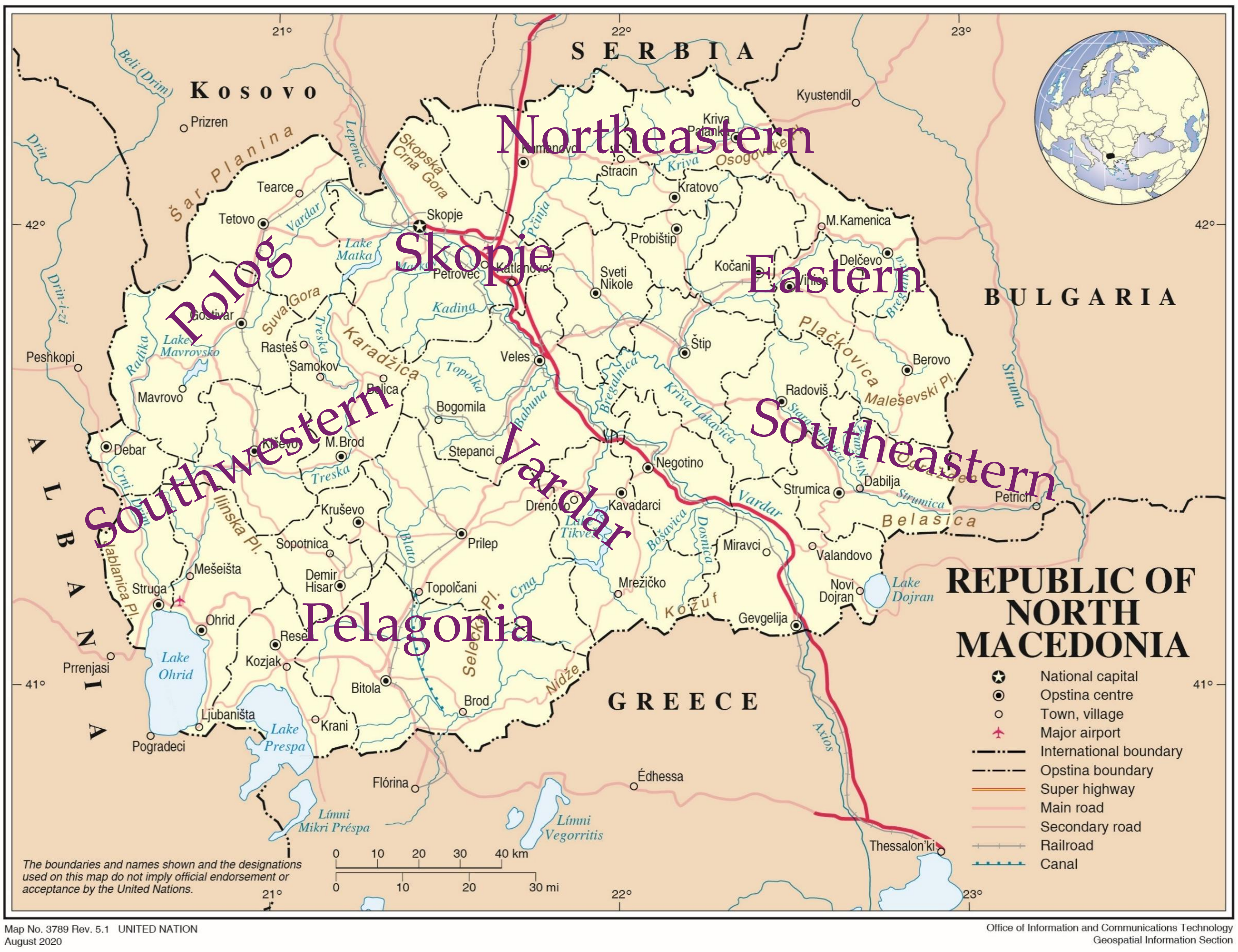

North Macedonia: Pelagonia, Polog, Skopje, Vardar, Eastern, Northeastern, Southeastern, Southwestern regions

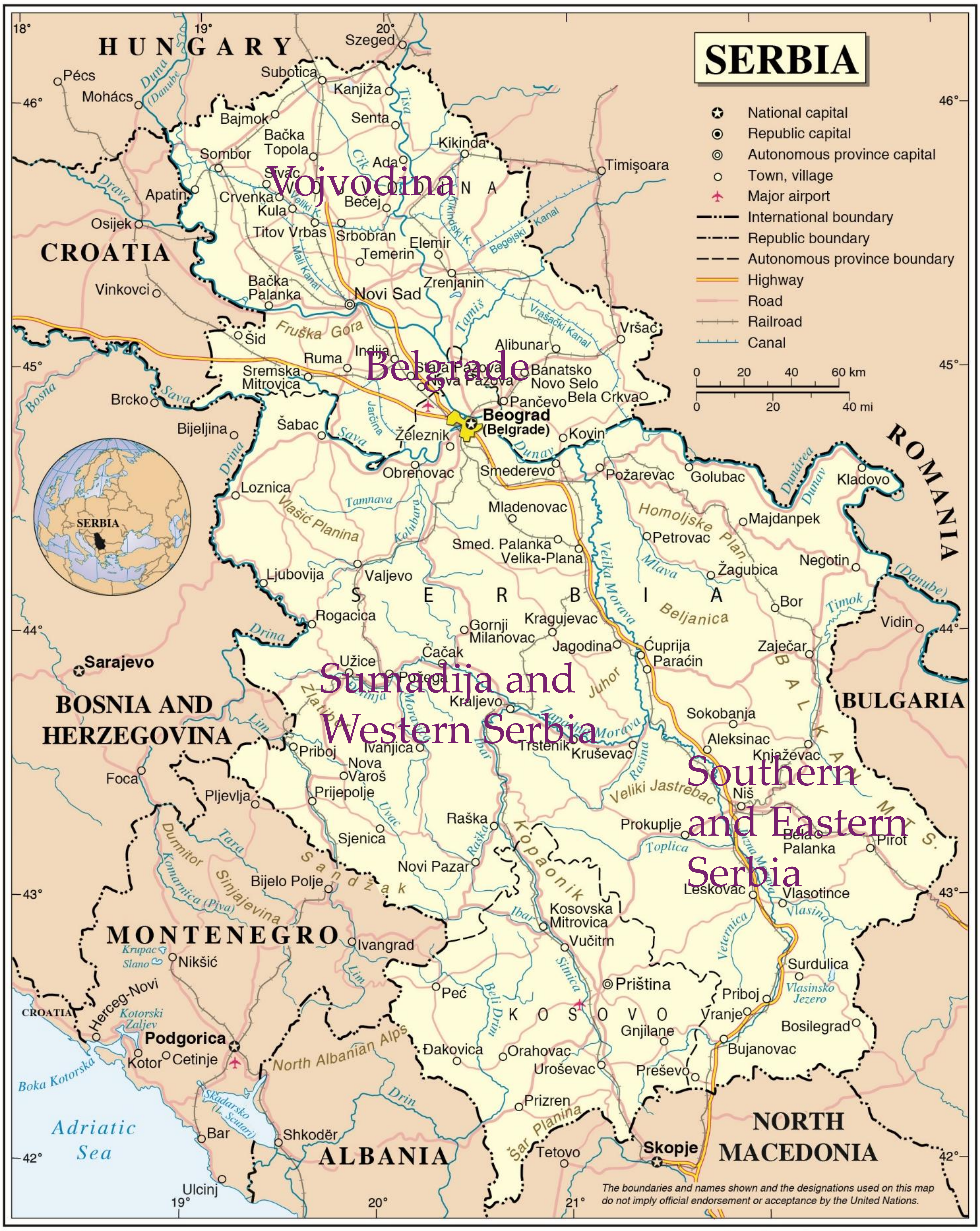

Map No. 4268 Rev.2 UNITED NATIONS
August 2020

Office of Information and Communications Technology
Geospatial Information Section

Serbia: Belgrade, Sumadija and Western Serbia, Southern and Eastern Serbia, Vojvodina statistical regions
