# Supplementary material for: Antimicrobial Use and Awareness of Antimicrobial Resistance in the Livestock Sector in the Western Balkans
Source: Antibiotics (Basel). 2025 Aug 19;14(8):839. doi: 10.3390/antibiotics14080839 (PMC12382872; doi:10.3390/antibiotics14080839)
Supplement: Supplementary file 1 [file antibiotics-14-00839-s001.zip › Survey instructions and questionnaires_Western Balkans.pdf]

<u>Instructions for the implementation of the antimicrobial use (AMU) survey</u>

Sections:

A. KoboCollect administration
B. Participant selection
C. Survey administration
D. Definitions for the antimicrobial use survey
E. Information to provide to participants on antimicrobial resistance
Appendix 1: Instructions for downloading forms and uploading surveys

A. KoboCollect Administration

1. In order to facilitate survey administration, the FAO will create accounts for a. the FAO national consultant in each country, b. a supervisor in each country and c. for each interviewer.
    a. The designated supervisor of interviewers will be responsible for data validation, and data quality. The account for this person will permit them to:
        i. View all surveys from interviewers in their country in order to ensure that the surveys are complete and correct.
        ii. Once the surveys have been reviewed by the supervisor, the "Submit" button should be clicked to initiate upload.
        iii. Once surveys have been submitted, they cannot be edited.
    b. The interviewer accounts will permit each interviewer to:
        i. enter data;
        ii. view and edit surveys that they have entered; and
        iii. save the surveys as “draft” in order to facilitate review by the supervisor.
        Interviewers will not be able to view other interviewers’ surveys.

2. Please see detailed instructions on downloading the survey forms and uploading the results in Appendix 1.
3. Surveys may be completed offline in areas where online completion is not possible. Surveys should be uploaded to the server as soon as reliable connectivity is available, preferably by the end of the day that the survey was completed.
4. All survey interviews will include the GPS coordinates of the premises. The GPS coordinate data will be destroyed at the conclusion of the project.
5. In order to facilitate quality assurance protocols, contact phone numbers will be collected for all survey interviews. This information will be destroyed at the conclusion of the project.
6. When all surveys have been completed, the dataset needs to be cleared at the country level.
7. Along with the dataset, the service provider must also submit:
    a. an Excel file with free text answers (related to answering “Other” options) with the question number, the answer in the original language, and the corresponding English translation, and
    b. pictures taken at the farms (see description in Section C).

B. Participant Selection

1. Regional Representation:

   Survey participants in each category should be enrolled from each of the selected regions as specified in the letter of agreement (LoA).

   If there are insufficient participants in a particular region, additional participants should be enrolled in other regions in order to achieve the desired national total.

2. Participant Selection:

   Survey participants should be identified and recruited as described in the LoA. If there are less than the required potential participants in any category, (for example feed mills) then efforts should be made to enrol all potential participants.

   If identified potential participants decline to participate in the survey, reasons for refusal should be recorded.

3. Participant Identification:

   i. All individual or personal information of participants will remain confidential.

   ii. Participants will be identified using Codes according to the following system.

   Country–Interviewer number–Survey type–Participant number

   a. Three letter country identifier
      i. Montenegro -MNE
   b. Interviewer identification
      i. Sequential numbers should be assigned to identify each interviewer, e.g. 01, 02, etc.
   c. Survey type identifier
      i. Farm – F
      ii. Veterinarian – V
      iii. Veterinary Pharmacy – P
   d. Participant identification
      i. Sequential numbers should be assigned to each participant interviewed by an interviewer, e.g. 001, 002, 003

   For example: the code for the first farm surveyed by interviewer #2 in Kyrgyzstan would be: Kyr-02–F-001

C. Survey Administration

It is essential that the **Informed Consent document** is explained, read and signed by each participant prior to beginning the survey. A signed copy of the Informed Consent must be provided to the participant and a copy must be retained by the service provider (SP) until the completion of the project. This document must NOT be provided to FAO. To facilitate quality assurance protocols, a contact phone number must be collected from all survey participants and retained until the completion of the project. This contact information will only be used to clarify survey responses and ensure data quality and will be destroyed upon completion of the project. The contact phone number must be linked with the Participant Code.

Feed Mills: Administer the survey to an owner or manager that has knowledge of the antimicrobial use policies and practices of the feed mill.

Veterinary Pharmacies: Administer the survey to an owner or manager that has knowledge of the policies and practices of the veterinary pharmacy.

Veterinarians: Administer the survey to a veterinarian or para-veterinarian who provides direct services to farmers (of the selected priority livestock species within the country).

Farmers: Administer the survey to an owner or manager that has knowledge of the antimicrobial use practices of the farm. The survey for farmers consists of several different sections. The sections completed on each farm will depend on the type of animals found on that farm. **<u>All farmers complete Section 1</u>**. **All applicable species-specific sections should also be completed (see diagram below).** The same Participant identification code should be used for all the sections that are completed for one farm.

For example:

If a farmer has commercial or semi-commercial broiler chickens, then the *Farmer Section* and the *Chickens Section* should be completed.
If a farmer has commercial or semi-commercial broiler chickens and commercial or semi-commercial bee production, then the *Farmer Section*, the *Chicken Section*, and the *Bee Section* should be completed.
If a farmer does not have any commercial or semi-commercial production, then the *Farmer Section* and the *Backyard Section* should be completed.

While administering the KoboCollect survey to all farmers, the surveyor should take one picture of the veterinary medicines (where they are stored), and at least one other picture of the place where animals are kept. Pictures should be labelled with the Participant Code followed by "Medicines" or "Farm" (for the first and second type of pictures, respectively).

Chicken survey: When completing the *Chicken Section*, questions referring to birds should only be answered with regards to chickens. Other types of poultry are not included in this section of the questionnaire

Cattle surveys: If cattle are kept for dual purpose (both for meat and milk), then the Dairy Cattle survey should be completed

Please follow the flow chart below for Farmer surveys.

ALL FARMERS NEED TO COMPLETE A MINIMUM OF TWO SURVEYS

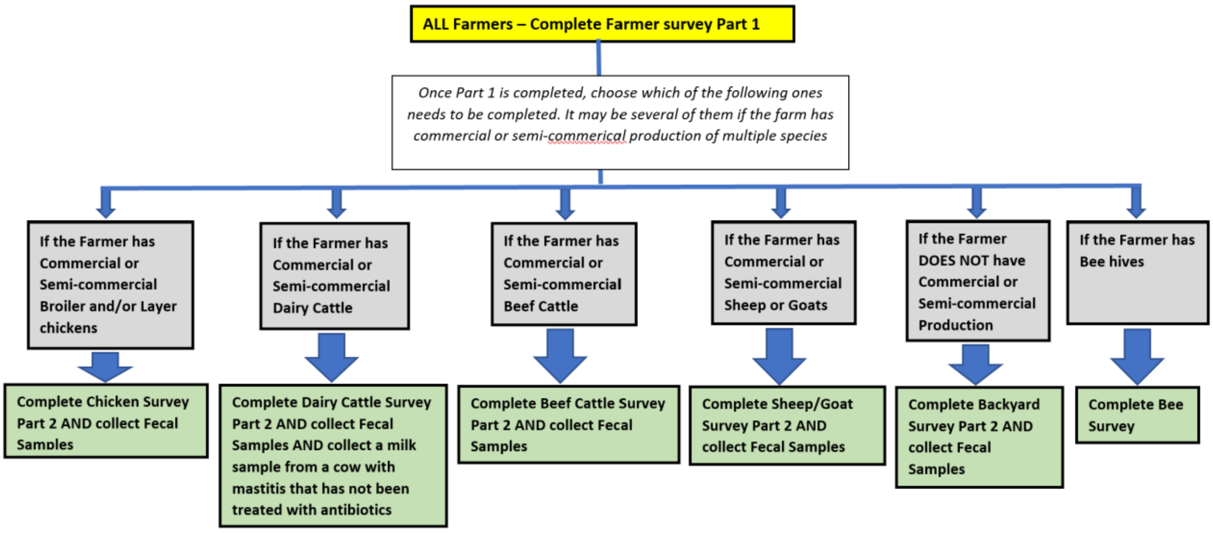

In the survey, there are directions to collect available **records** from participants for the previous 12 months if they are available. It is important to note that these records should be collected only IF the participant is willing to share these. Provision of these records is NOT a requirement of participation. However, if requested records are available and the participant is not willing to provide these, this should be recorded in the comments section at the end of the survey, as well as any reasons given for this decision.

In the survey, if there are questions that participants are **not willing to answer**, record this where indicated in the survey and provide the reasons given for not answering. Participants should not be pressured to provide answers; however, the interviewer should also avoid suggesting that not all questions need to be answered.

When misconceptions are identified, provide explanations and clarifications as described in Section E.

When the survey has been completed, provide the participant with the Antimicrobial Resistance information leaflet.

D. Definitions for the Antimicrobial Use Survey

Note: When translating these definitions, the terms used should be the same as the terms used in the KoboCollect survey.

Antimicrobial: antimicrobials are substances that kill or stop micro-organisms from growing and that help us treat diseases caused by microbes in humans, livestock, fish, plants and pets. For this survey, we are asking about antimicrobials that kill or stop bacteria from growing. These are often referred to as antibiotics.

Young animals: animals that have not reached breeding age.

Adult animals: animals that have reached breeding age.

Heifers: > 6 months old but have not given birth.

Piglets: pigs that are suckling the sow

Fattening pigs: pigs between the time of weaning and slaughter

Feed antimicrobials: antimicrobials that are mixed into or spread on top of the animal feed.

Water antimicrobials: antimicrobials that are mixed into the water that the animals drink.

Drench: an antimicrobial liquid that is given directly into the mouth.

Bolus: an antimicrobial tablet that is given directly into the mouth.

Topical: an antimicrobial that is applied to the skin or hooves/feet.

Backyard farm: an enterprise where the animals/birds raised are primarily for the consumption of the household.

Semi-commercial: an enterprise where the animals/birds are raised for the consumption of the household, as well as for sale, however this is not a primary source of income for the farmer.

Commercial: an enterprise where animals/birds are raised primarily for sale and this is a primary source of income for the farmer.

Treatment: antimicrobials given when a disease is present on the farm in at least one animal/bird.

Prevention: antimicrobials given to control disease when animals/birds when it is likely that they will become sick.

Growth promotion: antimicrobials given to make animals/birds grow bigger and/or faster, not to treat or control disease.

Withdrawal times (for vet survey)**:** the time required after giving an antimicrobial to an animal/bird before the animal/bird can be slaughtered for food or the milk or eggs from the animal can be consumed. This is to prevent antimicrobial residues in the meat or the milk, which can cause people to become sick.

On-farm culling: The animal/bird is euthanized and not used for meat.

Education: As part of the curriculum towards a degree or certificate.

Training: Short-term learning opportunity.

E. Information to provide to participants on antimicrobial resistance

The information provided in this section is intended to increase the participants' awareness and understanding of antimicrobial resistance. Other than the question on what antibiotics are, this information should not be provided to the participant until AFTER they have finished the survey.

1. **This information should be provided to the participant <u>immediately after they have answered the question</u>.**

Question:

Do you know what antimicrobials are? Yes No

If yes, please circle all that apply:

medicine that prevents disease
medicine that kills disease
medicine that kills germs
medicine that kills bacteria
medicine that makes animals grow faster/bigger
medicine that kills viruses
medicine that kills parasites
Other: ________________________________

Answers:

- If participant answers **No**
  - Microorganisms are everywhere and include bacteria, viruses, parasites and fungi. They include bacteria that can sometimes cause disease and infection in humans, animals and plants.
  - Antimicrobials are substances that kill or stop microorganisms from growing and that help us treat diseases caused by microbes in humans, livestock, fish, plants and pets. For this survey, we are asking about antimicrobials that kill or stop bacteria from growing. These are often referred to as antibiotics.

- If participant answers **Yes** to the first part AND answers **Yes to either "medicine that kills viruses"** or **"medicine that kills parasites"**:
  - Antimicrobials are substances that kill or stop microorganisms from growing and that help us treat diseases caused by microbes in humans, livestock, fish, plants and pets. For this survey, we are only asking about antimicrobials that kill or stop <u>bacteria</u> from growing. These are often referred to as antibiotics.

2. **The information for the questions below should be provided to the participant <u>at the conclusion of the survey</u>**

Question:

If the antimicrobials that you have become expired, what do you do?

Use them
Throw them in the garbage
Consult with a veterinarian (if yes pharmacy, private, government)
Pour them down the drain/sink
Return them to where you purchased them
I don’t look at the expiration date
Expiration date is not on the medications I use

Answers:

- If answer **"Use them"** or **"I don't look at the expiration date"**:
    - Expired medicines may have lost much of their potency or might even be harmful to the diseased animal.
    - They may also contribute to the development of resistant bacteria in the animal, herd or flock, which may make it more difficult to treat infections in these animals in the future.
- If answer **"Throw them in the garbage"** or **"Pour them down the drain/sink"**
    - Expired medicines may be harmful to the environment. They may contribute to the development of resistant bacteria in the environment, which may make it more difficult to treat infections in animals and people in the future. They should be returned to the retailer, e.g. pharmacy, if possible.
- If answer **"Expiration date is not on the medications I use"**:
    - This may be an indication that the medication you are using is not made by a reputable company. Medications are effective only for a specific time period.
    - Expired medicines may have lost much of their potency or might even be harmful to the diseased animal.
    - They may also contribute to the development of resistant bacteria in the animal, herd or flock, which may make it more difficult to treat infections in these animals in the future.

Question: How much do you agree with the following statements:

You can stop giving antimicrobials to an animal if their symptoms are improving

If **agree**: Antimicrobials should be given until the end of the period that is on the label or was recommended by your veterinarian. Even when an animal's symptoms are improving, the bacteria that caused the symptoms may still be present and if you stop giving the antimicrobials, the bacteria will cause disease again and the antimicrobials may not work at all because bacteria have become resistant.

If antibiotics are given too often they might stop working

If **disagree**:

Bacteria can become resistant to antibiotics when repeatedly exposed to them and then the antibiotic will not work the way it used to. Using antibiotics only when necessary will help to avoid this.

Giving antimicrobials to healthy animals will prevent them from getting sick in the future

If **agree**:

Antimicrobials can treat diseases caused by bacteria, sometimes just before symptoms appear. However, antimicrobials cannot prevent animals from getting diseases in the future.

Using vaccines can prevent the use of antibiotics

If **disagree**:

Vaccines for specific diseases can protect animals from becoming sick with those diseases. When the animals stay healthy, the antimicrobials are not needed.

Animals can transmit disease to humans

If **disagree**:

Some microorganisms can be present in more than one host or species, including both animals and people. These microorganisms can spread from animals to

humans, through food, direct contact or the environment. Sometimes these microorganisms do not make animals sick but can make people ill.

Antimicrobial use in animals does not affect human health

If **agree**:

Antimicrobial use in animals can affect human health in several different ways. When microorganisms become resistant to a particular antimicrobial, they can then infect different hosts, including humans, through the food chain or the environment. The antimicrobial will then no longer work to treat the infection or disease in humans.

Antimicrobial use in animals can also affect human health when antimicrobials are still present in meat, milk, or honey when consumed by people. This is called antimicrobial residue and can cause problems in people who are allergic or have a negative physical reaction when exposed to these antimicrobials.

Antimicrobials may be freely discarded without having an action/effect on the environment

If **agree**:

When antimicrobials are discarded improperly, they can persist in the environment. This can lead to antimicrobial resistance.

Antimicrobial resistance occurs when antibiotics are found in the meat or milk of an animal.

If **agree**:

When antimicrobials are found in the meat or milk of an animal or in honey, this is an antimicrobial residue. Antimicrobial residues can cause problems in people who are allergic or have a negative physical reaction when exposed to these antimicrobials. However, antimicrobial resistance is when microorganisms become resistant to a particular antimicrobial. The antimicrobial will then no longer work to treat the infection or disease in animals or humans.

When you use antimicrobials, there is a certain number of days you should wait before selling the animals for slaughter, selling eggs, milk or honey.

If **disagree**:

When animals or birds or bees are given antimicrobials, the antimicrobials can be present in the meat, milk, eggs or honey for a certain number of days after the last time they were given. When antimicrobials are still present in meat, milk, eggs or honey when it is consumed by people, this is called antimicrobial residue and can cause problems in people who are allergic or have a negative physical reaction when exposed to these antimicrobials.

With prevention and early detection, you can reduce the use of antimicrobials

If **disagree**:

When animals/birds/bees are fed a good diet, are in a good environment, and are not exposed to other animals/birds/bees that are carrying disease, they are less likely to get sick and require antimicrobial treatment. Therefore, disease is prevented and antimicrobial use is reduced. If disease in animals/birds/bees is detected when they first become sick or when it is present in only a few animals/birds/bees, then less antimicrobials are often required to make the animals/birds/bees healthy again. It is also possible that less animals/birds/bees will die.

<u>**Appendix 1: Instructions for downloading forms and uploading surveys**</u>

In the drop-down menu under "Collect data," you have several options available:

Choose “Online-Offline (multiple submission)”: This allows online and offline submissions and is the best option for collecting data in the field. Then press “OPEN” to open the survey form to a new tab in your browser. Once the form is opened, you should see a screen like the one shown in the image below:

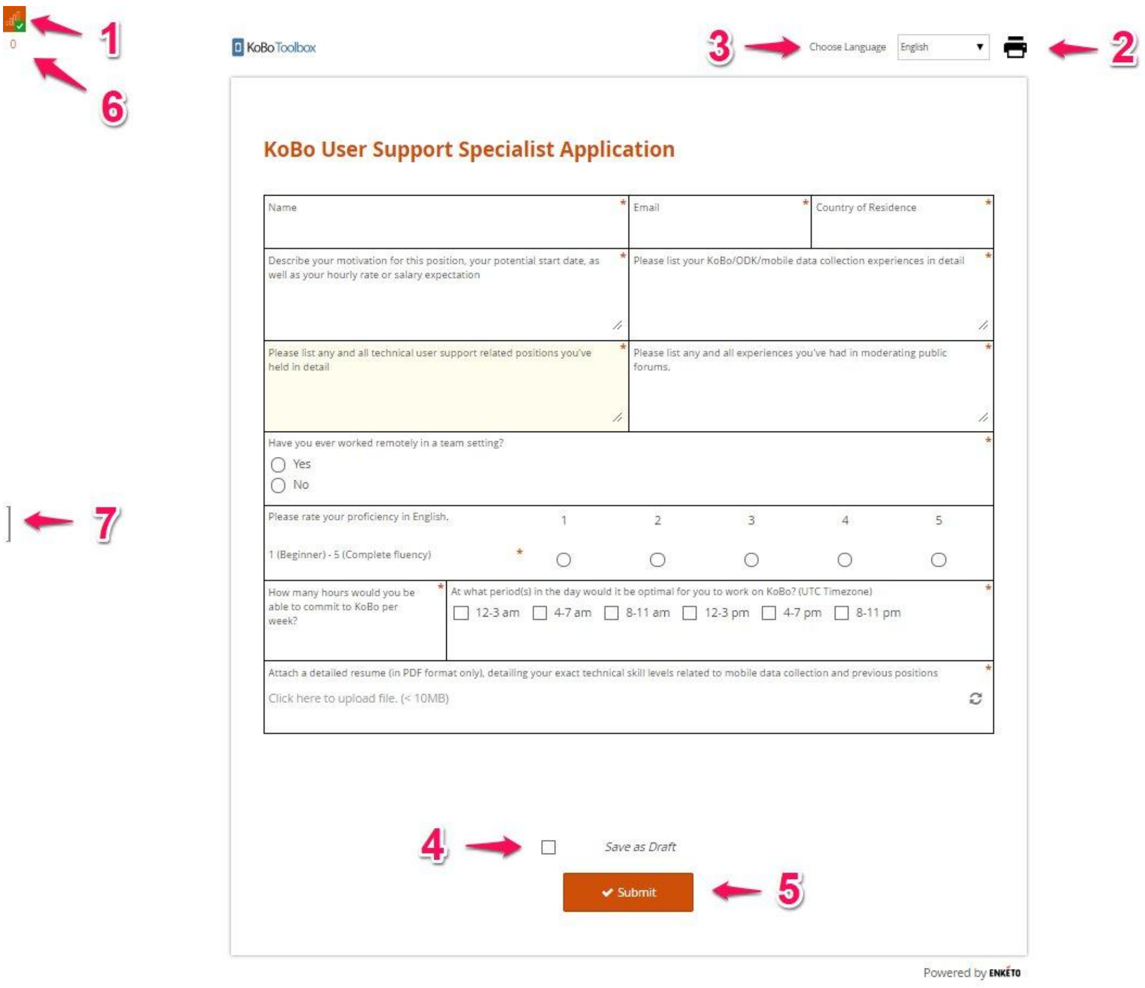

1. To enable offline data entry:

   a) Open the survey link provided (as was done during the pre-testing).

   b) Select “Add to Home screen” from the settings.

   c) Touch and hold the “Add” tab to add it to the home screen.

   d) The KoboCollect icon will appear on the screen of the tablet and the Interviewer will easily open the file from the screen during the surveys.

2. Printer icon: The printer icon provides you access to save it as a PDF version. For this, press the printer icon and then select Destination (“Save as PDF” to save your survey form as a PDF).

3. Choose language: The survey is available in various languages used in the Europe and Central Asia region. You’re able to toggle between the default language (English) and other languages present in the survey form.

4. Save as draft: Use this feature to edit or update the record before submitting it to the KoboToolbox server. Once you have checked "Save as Draft" you will have an option to "Save Draft." The draft record gets queued but does not sync with KoboToolbox server. To sync it with the server the Supervisor will open the record from the queued list and uncheck "Save as Draft" and press "Submit."

5. Submit: The Supervisor will press the "Submit" button once the information in a survey is verified as final and complete and ready to be uploaded to the KoboToolbox server. After pressing the "Submit" button, you will not have an option to edit the records on your device.

6. Queued records counter: The queued records counter shows you the total number of records submitted and waiting to be uploaded to a server. The queued records are uploaded automatically in the background every 5 minutes when the web page is open and an internet connection is available. The application will always attempt to upload data immediately and will retry until a connection has been established again.

All synchronization is proofed even against poor Internet connection quality. Should a connection time out or be interrupted while a specific form is being transferred, it will be resent with the next upload attempt. The server will not integrate half received data in this case. Only when a record has been uploaded successfully and the server confirms receipt will the survey data be removed from the upload queue.

7. Queue records pane: Clicking the side button shows you the records that are available as drafts (which can still be edited) and finalized submitted records queued to be uploaded to the server with an internet connection.

8. Open-ended Questions:

> Questions that are answered using text in the language of the country, for example “If other, please specify,” will be recorded in the database in the original language. In addition, an Excel file should be created with the following columns:
>
> Participant code Question number Original version Translated English

Farmer Section _Part 1_FAO AMU Survey

I. Has the informed consent form been explained, read by the participant and signed (please, accept)? *

- ◯ OK

II. Interviewer Identification code *

III. Date of Interview/Survey *

yyyy-mm-dd

IV. Farmer ID *

Please, collect the GPS point

latitude (x.y °)

longitude (x.y °)

altitude (m)

accuracy (m)

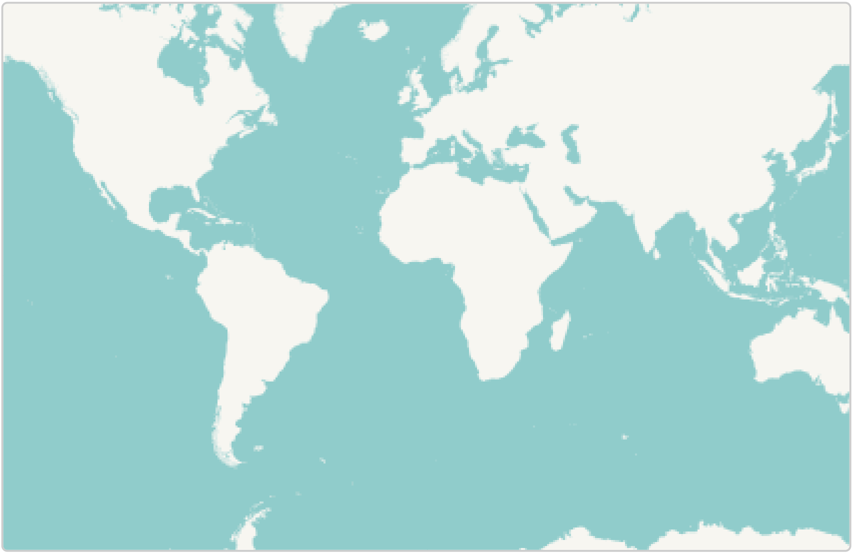

V. Choose the country *

- ◯ North Macedonia
- ◯ Bosnia and Herzegovina
- ◯ Serbia
- ◯ Albania
- ◯ Montenegro
- ◯ Kosovo

1. What Region/Districts/Counties/Canton is this farm in? *

- Eastern
- Northeastern
- Pelagonia
- Polog
- Skopje
- Southeastern
- Southwestern
- Vardar

1. What Region/Districts/Counties/Canton is this farm in? *

- Una-Sana
- Posavina
- Tuzla
- Zenica-Doboj
- Bosnian Podrinje
- Central Bosnia
- Herzegovina-Neretva
- West Herzegovina
- Sarajevo
- Canton 10
- Region Banja Luka
- Region Prijedor
- Region Doboj
- Region Bijeljina
- Region Istocno Sarajevo
- Region Trebinje

1. What Region/Districts/Counties/Canton is this farm in? *

- Belgrade
- Vojvodina
- Šumadija and Western Serbia
- Southern and Eastern Serbia
- Kosovo and Metohija

1. What Region/Districts/Counties/Canton is this farm in? *

- Tirana
- Elbasan
- Vlore
- Korce
- Dibra
- Kukes
- Durres
- Shkoder
- Fier
- Lezha
- Berat
- Gjirokastra

1. What Region/Districts/Counties/Canton is this farm in? *

- Central Region
- Coastal Region
- Northern Region

1. What Region/Districts/Counties/Canton is this farm in?

- District of Ferizaj
- District of Gjakova
- District of Gjilan
- District of Mitrovica
- District of Peja
- District of Pristina
- District of Prizren

1.1. Village/city/settlement

2. What type of farm is this?

- ◯ Large commercial Farm
- ◯ Semi-commerical Farm
- ◯ Government Farm
- ◯ Backyard Farm
- ◯ Other

Other, please specify

3. On this farm, are you

The owner

- ◯ Yes
- ◯ No

The manager

- ◯ Yes
- ◯ No

An employee

- ◯ Yes
- ◯ No

4. Are you a veterinarian

- ◯ Yes
- ◯ No

5. Have you had any training in the following areas

Animal health

- ☐ Yes, education
- ☐ Yes, training
- ☐ No

if yes, please select what education

- High school
- College education
- Higher education/master degree

Animal husbandry

- Yes, education
- Yes, training
- No

if yes, please select what education

- High school
- College education
- Higher education/master degree

Pharmacology

- Yes, education
- Yes, training
- No

if yes, please select what education

- High school
- College education
- Higher education/master degree

Other

- Yes, education
- Yes, training
- No

Other, please specify

if yes, please select what education

- High school
- College education
- Higher education/master degree

6. How many years have you worked in livestock farming

7. What is your age?

- ◯ <25 years old
- ◯ 25-40 years old
- ◯ 41-55 years old
- ◯ > 55 years old

8. What is your Gender?

- ◯ Male
- ◯ Female

9. Do you have the following farm records?

Net income

- ◯ Yes
- ◯ No

Animal medicines purchased

- ◯ Yes
- ◯ No

Treatment records

- ◯ Yes
- ◯ No

Vaccination records

- ◯ Yes
- ◯ No

Treatment protocols

- ◯ Yes
- ◯ No

Veterinarian visits

- ◯ Yes
- ◯ No

Prescription records

- ◯ Yes
- ◯ No

Amount of Feed manufactured on the farm

- ◯ Yes
- ◯ No

Amount of animals/eggs/milk sold

- ◯ Yes
- ◯ No

Amount of Feed Purchased

- ◯ Yes
- ◯ No

Mortality

- ◯ Yes
- ◯ No

Births

- ◯ Yes
- ◯ No

Other

- ◯ Yes
- ◯ No

Other, please specify

....

10. What type and number of animals are on your farm today

....

Type of animals	Number of chicks/young animals	Number of adult animals
**Chickens for meat (broilers)**	Number of chicks	Number of adult poultry
**Chickens for eggs (layers)**	Number of chicks	Number of adult poultry
**Geese**	Number of chicks	Number of adult poultry
**Ducks**	Number of chicks	Number of adult poultry
**Turkeys**	Number of chicks	Number of adult poultry
**Cattle for meat**	Number of young animals	Number of adult animals
**Cattle for milk**	Number of young animals	Number of adult animals
**Horses for meat**	Number of young animals	Number of adult animals
**Horses for milk**	Number of young animals	Number of adult animals
**Sheep**	Number of young animals	Number of adult animals
**Goats**	Number of young animals	Number of adult animals

**Pigs**	Number of young animals	Number of adult animals
**Rabbits**	Number of young animals	Number of adult animals
**Bees**	Number of nucleus hives (Beginner hives) /Swarms	Number production hives (colonies)
**other**	Number of chicks/young animals	Number of adult animals

Other, please specify

11. Do you know what antibiotics are?

- ◯ Yes
- ◯ No

11.1 If yes, please list all that apply

- ☐ medicine that prevents disease
- ☐ medicine that kills disease
- ☐ medicine that kills germs
- ☐ medicine that kill bacteria
- ☐ medicine that makes animals grow faster/bigger
- ☐ medicine that kills viruses
- ☐ medicine that kills parasites
- ☐ other

Other, please specify

12. Do you use antibiotics in your animals? (If no, go to Animal species part of survey)

- ◯ Yes
- ◯ No

13. Where do you buy your antibiotics?

Veterinary pharmacy

- All of the time
- Often
- Rarely
- Never

Human pharmacy

- All of the time
- Often
- Rarely
- Never

Private veterinarian

- All of the time
- Often
- Rarely
- Never

Government veterinarian

- All of the time
- Often
- Rarely
- Never

Local Market

- All of the time
- Often
- Rarely
- Never

Feed mill

- All of the time
- Often
- Rarely
- Never

Directly from outside of the country

- All of the time
- Often
- Rarely
- Never

Wholesalers

- All of the time
- Often
- Rarely
- Never

Other farmers

- All of the time
- Often
- Rarely
- Never

Other

- All of the time
- Often
- Rarely
- Never

Other, please specify

....

14. During or after the lockdown due to the COVID-19 situation, have you experienced any of the following?

....

Problems accessing antibiotics

- Yes
- No

Problems accessing disinfectants

- Yes
- No

Problems accessing vaccines

- Yes
- No

Increased mortality in your animals

- Yes
- No

Problems accessing veterinary support

- Yes
- No

Need to use antibiotics more often than normally

- Yes
- No

Need to use increased doses of antibiotics for your animals

- Yes
- No

Need to use expired antibiotics

- Yes
- No

15. Do you buy antibiotics following a veterinary prescription?

- All of the time
- Often
- Occasionally
- Rarely
- Never

16. Who makes the decision on when to use an antibiotic?

Farm owner

- All of the time
- Often
- Rarely
- Never

Farm manager

- All of the time
- Often
- Rarely
- Never

Private veterinarian

- All of the time
- Often
- Rarely
- Never

Government veterinarian

- All of the time
- Often
- Rarely
- Never

Company veterinarian

- All of the time
- Often
- Rarely
- Never

Laboratory veterinarian/technician

- All of the time
- Often
- Rarely
- Never

Feed mill

- All of the time
- Often
- Rarely
- Never

other

- All of the time
- Often
- Rarely
- Never

Other, please specify

17. Where do you get advice/information on which antibiotics to use for specific problems and how much to use and how long to use them?

Veterinary pharmacist

- All of the time
- Often
- Rarely
- Never

Private veterinarian

- All of the time
- Often
- Rarely
- Never

Company veterinarian

- All of the time
- Often
- Rarely
- Never

Government veterinarian

- All of the time
- Often
- Rarely
- Never

Internet

- All of the time
- Often
- Rarely
- Never

Other farmers

- All of the time
- Often
- Rarely
- Never

Friends/family

- All of the time
- Often
- Rarely
- Never

Feed mill

- All of the time
- Often
- Rarely
- Never

From product label

- All of the time
- Often
- Rarely
- Never

From previous experience

- All of the time
- Often
- Rarely
- Never

Veterinary laboratory results

- All of the time
- Often
- Rarely
- Never

other

- All of the time
- Often
- Rarely
- Never

Other, please specify

18. When using antibiotics, do you:

a) Dose choices

- Give more (higher dose)
- Give less (lower dose)
- Give the same dose as the advice

b) Time choices

- Give for longer time
- Give for shorter time
- Give for the same time as the advice

19. If the antibiotics that you have become expired, what do you do?

- Use them
- Throw them in the garbage
- Consult with a veterinarian
- Pour them down the drain/sink
- Return them to where you purchased them
- I don't look at the expiration date
- Expiration date is not on the medications I use
- other

if Consult with a veterinarian, please note what veterinarian?

- [ ] Private
- [ ] Government
- [ ] other

Other, please specify

....

Other, please specify

....

20. How much do you agree with the following statements?

....

You can stop giving antibiotics to an animal if their symptoms are improving

- Agree
- Disagree
- Don't know

If antibiotics are given too often they might stop working

- Agree
- Disagree
- Don't know

Giving antibiotics to healthy animals will prevent them from getting sick in the future

- Agree
- Disagree
- Don't know

Using vaccines can prevent the use of antibiotics

- Agree
- Disagree
- Don't know

Animals can transmit disease to humans

- Agree
- Disagree
- Don't know

Antibiotic use in animals does not affect human health

- Agree
- Disagree
- Don't know

Antibiotics may be freely discarded without having an action/effect on the environment

- Agree
- Disagree
- Don't know

You have heard of antibiotic resistance

- Agree
- Disagree
- Don't know

Antibiotic resistance occurs when antibiotics are found in the meat or milk of an animal

- Agree
- Disagree
- Don't know

When you use antibiotics there is a certain number of days you should wait before selling the animals for slaughter, selling eggs, milk or honey

- Agree
- Disagree
- Don't know

With prevention and early detection you can reduce the use of antibiotics

- Agree
- Disagree
- Don't know

21. Do you believe that antibiotics are:

- As effective as they always were
- A little less effective
- Much less effective
- Effective if I use more than I used to
- I don't know

22. Would you be interested in learning more about how to use antibiotics?

- Yes
- No

23. Have the photos of all antibiotics on the farm today been made (please, accept)? (Please take clear pictures of all antibiotics on the farm today, including medicated feeds and injectables, plus transcribe them below once you return to the office)

- Yes
- No

24. Did the participant refuse to answer any of these questions?

- ◯ Yes
- ◯ No

If yes, list the number(s) of these questions

If yes, check all reasons that participant gave for this refusal?

- ☐ Concerned about researchers knowing this information
- ☐ Concerned about government knowing this information
- ☐ Concerned about other farmers knowing this information
- ☐ Other

Other, please specify

Beef _Farmer Survey Part 2_FAO AMU Survey

Interviewer ID *

Interview Date *

yyyy-mm-dd

Farmer ID *

Choose the country *

- North Macedonia
- Bosnia and Herzegovina
- Serbia
- Albania
- Montenegro
- Kosovo

1. Over the last 12 months, what is the minimum number and the maximum number of each type of animal that you have had on your farm?

Stages of Life	Minimum number in last 12 months	Maximum number in last 12 months	Average number of animals on the farm over the last 12 months
**Cows**			

**Bulls**			
**Calves under 6 months**			
**Young cattle (Heifers/Steers) over 6 months**			

2. If you do any of the following when your cattle get sick, what order would you follow (e.g. 1st, 2nd, 3rd, etc.)	1st	2nd	3rd	4th	5th	6th	7th
Treat with antibiotics	○	○	○	○	○	○	○
Isolate the sick animal(s)	○	○	○	○	○	○	○
Call a veterinarian	○	○	○	○	○	○	○
Send samples to the diagnostic laboratory	○	○	○	○	○	○	○
Slaughter them for meat	○	○	○	○	○	○	○
On farm culling	○	○	○	○	○	○	○
Treat with non-antibiotic medications	○	○	○	○	○	○	○
Other	○	○	○	○	○	○	○

None of the above

○ OK

Other, please specify

3. Estimate the percentage of your cattle that receive antimicrobials at least once before they go to market (including medicated feed over the last 12 months.

4. What percentage of your animals died of disease over the last 12 months?

Cows

Calves under 6 months

Heifers/Steers over 6 months

5. Which of the following do you do on your farm (please check all that apply?

Buy new breeding animals from more than one source

- Yes, always
- Yes, sometimes
- No
- Don't know

Keep new animals introduced onto farm separately for at least 30 days.

- Yes, always
- Yes, sometimes
- No
- Don't know

Share bulls with other farmers

- Yes, always
- Yes, sometimes
- No
- Don't know

Use purchased semen for artificial insemination

- Yes, always
- Yes, sometimes
- No
- Don't know

Use shared/community pastures

- Yes, always
- Yes, sometimes
- No
- Don't know

Share equipment with other farmers

- Yes, always
- Yes, sometimes
- No
- Don't know

Clean and disinfect equipment if shared with other farmers

- Yes, always
- Yes, sometimes
- No
- Don't know

Keep sick animals separately

- Yes, always
- Yes, sometimes
- No
- Don't know

Keep cattle enclosed in pastures/ pens

- Yes, always
- Yes, sometimes
- No
- Don't know

Provide boots for visitors

- Yes, always
- Yes, sometimes
- No
- Don't know

Wheel dipping at the farm entrance

- Yes, always
- Yes, sometimes
- No
- Don't know

Register visitors

- Yes, always
- Yes, sometimes
- No
- Don't know

Prevent rodents and pests in the barn(s)

- Yes, always
- Yes, sometimes
- No
- Don't know

Have other domestic animals in the cattle barn

- Yes, always
- Yes, sometimes
- No
- Don't know

Keep wild animals at least 30 m from your animals/birds

- Yes, always
- Yes, sometimes
- No
- Don't know

Prevent wild birds from accessing the feed storage

- Yes, always
- Yes, sometimes
- No
- Don't know

6. Please complete the following table on your herd for the last 12 months

Calves under 6 months

**Disease/ Syndrome**	**Affected (%)**	**Mortality (%)**	**Vaccinated (%)**

**Anaplasmosis**			
**Babesiosis**			
**Brucellosis**			
**Nervous system problems**			
**Footrot**			
**Other lameness**			
**Diarrhea**			
**Hardware**			
**Other intestinal problems**			
**Breathing problems**			

**Other**			

Other Disease/ Syndrome, please specify

Heifers

Disease/ Syndrome	Affected (%)	Mortality (%)	Vaccinated (%)
**Anaplasmosis**			
**Babesiosis**			
**Brucellosis**			
**Nervous system problems**			
**Footrot**			
**Other lameness**			
**Diarrhea**			

**Hardware**			
**Other intestinal problems**			
**Metritis**			
**Other Reproductive problems**			
**Breathing problems**			
**Other**			

Other Disease/ Syndrome, please specify

Cows

**Disease/ Syndrome**	**Affected (%)**	**Mortality (%)**	**Vaccinated (%)**
**Anaplasmosis**			

**Babesiosis**			
**Brucellosis**			
**Nervous system problems**			
**Footrot**			
**Other lameness**			
**Diarrhea**			
**Hardware**			
**Other intestinal problems**			
**Mastitis**			
**Metritis**			

**Other Reproductive problems**			
**Breathing problems**			
**Other**			

Other Disease/ Syndrome, please specify

6.1 Have you experienced an in increased incidence of any of these diseases since the lockdown due to COVID?

- ◯ Yes
- ◯ No

If yes, please specify

7. Do you use antibiotics to make healthy animals grow faster/bigger even if the antibiotics are not needed to prevent or treat disease?

- ◯ Yes
- ◯ No

7.1 If yes, please complete the following questions

1

1.What Antibiotic (use numbered list provided)

2. Stages of Life

- Cows
- Heifers
- Calves

3. How

- Feed
- Water

4. For how many days in a row on average?

5. What percentage of animals per year?

8. Please list maximum of 5 antibiotics you've used most frequently for treatment or prevention in the last 12 months including medicated feed

The farmer does not use any antibiotic (to end the survey)

- OK

the 1st antibiotic

the 2nd antibiotic

the 3rd antibiotic

the 4th antibiotic

the 5th antibiotic

Please complete the following questions for the 1st Antibiotic

2. Reason for Antibiotic (from Disease/Syndrome)

- Anaplasmosis
- Babesiosis
- Brucellosis
- Nervous system problems
- Footrot
- Other lameness
- Diarrhea
- Hardware
- Other intestinal problems
- Mastitis
- Metritis
- Other Reproductive problems
- Breathing problems
- Other

Other, please specify

3. Stage of Life

- Cows
- Heifers
- Calves

4. Aim

- Treatment
- Prevention

5. How

- Feed
- Water
- Injection
- Topical
- Drench
- Bolus
- In the teat

6. For how many days in a row on average?

7. What percentage of animals per year?

8. If this antibiotic doesn't work, what do you do?

- Repeat the treatment with the same antibiotic
- Consult with a veterinarian
- Use a higher dose of the same antibiotic
- Use a different antibiotic
- Do nothing
- Send for slaughter
- Kill and discard the animal
- Slaughter for my own consumption
- Other

which one?

Do you test samples to identify the pathogen?

- Yes
- No

Do you wait for antibiotic susceptibility information from the laboratory?

- Yes
- No

Other, please specify

Please complete the following questions for the 2nd Antibiotic

2. Reason for Antibiotic (from Disease/Syndrome)

- Anaplasmosis
- Babesiosis
- Brucellosis
- Nervous system problems
- Footrot
- Other lameness
- Diarrhea
- Hardware
- Other intestinal problems
- Mastitis
- Metritis
- Other Reproductive problems
- Breathing problems
- Other

Other, please specify

3. Stage of Life

- Cows
- Heifers
- Calves

4. Aim

- Treatment
- Prevention

5. How

- Feed
- Water
- Injection
- Topical
- Drench
- Bolus
- In the teat

6. For how many days in a row on average?

7. What percentage of animals per year?

8. If this antibiotic doesn't work, what do you do?

- Repeat the treatment with the same antibiotic
- Consult with a veterinarian
- Use a higher dose of the same antibiotic
- Use a different antibiotic
- Do nothing
- Send for slaughter
- Kill and discard the animal
- Slaughter for my own consumption
- Other

which one?

Do you test samples to identify the pathogen?

- Yes
- No

Do you wait for antibiotic susceptibility information from the laboratory?

- Yes
- No

Other, please specify

Please complete the following questions for the 3rd Antibiotic

2. Reason for Antibiotic (from Disease/Syndrome)

- Anaplasmosis
- Babesiosis
- Brucellosis
- Nervous system problems
- Footrot
- Other lameness
- Diarrhea
- Hardware
- Other intestinal problems
- Mastitis
- Metritis
- Other Reproductive problems
- Breathing problems
- Other

Other, please specify

3. Stage of Life

- Cows
- Heifers
- Calves

4. Aim

- Treatment
- Prevention

5. How

- Feed
- Water
- Injection
- Topical
- Drench
- Bolus
- In the teat

6. For how many days in a row on average?

7. What percentage of animals per year?

8. If this antibiotic doesn't work, what do you do?

- Repeat the treatment with the same antibiotic
- Consult with a veterinarian
- Use a higher dose of the same antibiotic
- Use a different antibiotic
- Do nothing
- Send for slaughter
- Kill and discard the animal
- Slaughter for my own consumption
- Other

which one?

Do you test samples to identify the pathogen?

- Yes
- No

Do you wait for antibiotic susceptibility information from the laboratory?

- Yes
- No

Other, please specify

Please complete the following questions for the 4th Antibiotic

2. Reason for Antibiotic (from Disease/Syndrome)

- Anaplasmosis
- Babesiosis
- Brucellosis
- Nervous system problems
- Footrot
- Other lameness
- Diarrhea
- Hardware
- Other intestinal problems
- Mastitis
- Metritis
- Other Reproductive problems
- Breathing problems
- Other

Other, please specify

3. Stage of Life

- Cows
- Heifers
- Calves

4. Aim

- Treatment
- Prevention

5. How

- Feed
- Water
- Injection
- Topical
- Drench
- Bolus
- In the teat

6. For how many days in a row on average?

7. What percentage of animals per year?

8. If this antibiotic doesn't work, what do you do?

- Repeat the treatment with the same antibiotic
- Consult with a veterinarian
- Use a higher dose of the same antibiotic
- Use a different antibiotic
- Do nothing
- Send for slaughter
- Kill and discard the animal
- Slaughter for my own consumption
- Other

which one?

Do you test samples to identify the pathogen?

- Yes
- No

Do you wait for antibiotic susceptibility information from the laboratory?

- Yes
- No

Other, please specify

Please complete the following questions for the 5th Antibiotic

2. Reason for Antibiotic (from Disease/Syndrome)

- Anaplasmosis
- Babesiosis
- Brucellosis
- Nervous system problems
- Footrot
- Other lameness
- Diarrhea
- Hardware
- Other intestinal problems
- Mastitis
- Metritis
- Other Reproductive problems
- Breathing problems
- Other

Other, please specify

3. Stage of Life

- Cows
- Heifers
- Calves

4. Aim

- Treatment
- Prevention

5. How

- Feed
- Water
- Injection
- Topical
- Drench
- Bolus
- In the teat

6. For how many days in a row on average?

7. What percentage of animals per year?

8. If this antibiotic doesn't work, what do you do?

- Repeat the treatment with the same antibiotic
- Consult with a veterinarian
- Use a higher dose of the same antibiotic
- Use a different antibiotic
- Do nothing
- Send for slaughter
- Kill and discard the animal
- Slaughter for my own consumption
- Other

which one?

Do you test samples to identify the pathogen?

- Yes
- No

Do you wait for antibiotic susceptibility information from the laboratory?

- Yes
- No

Other, please specify

9. Did the participant refuse to answer any of these questions?

- Yes
- No

If yes, list the number(s) of these questions

If yes, check all reasons that participant gave for this refusal

- Concerned about researchers knowing this information
- Concerned about government knowing this information
- Concerned about other farmers knowing this information
- Other

Other, please specify

Comments

Dairy _Farmer Survey Part 2_FAO AMU Survey

Interviewer ID *

Interview Date *

yyyy-mm-dd

Farmer ID *

Choose the country *

- North Macedonia
- Bosnia and Herzegovina
- Serbia
- Albania
- Montenegro
- Kosovo

1. Over the last 12 months, what is the minimum number and the maximum number of each type of animal that you have had on your farm?

Stages of Life	Minimum number in last 12 months	Maximum number in last 12 months	Average number of animals on the farm over the last 12 months
Cows (milking and dry)			
Bulls			

**Calves under 6 months**			
**Heifers (include a hint "Heifers= more than 6 months old but have not calved yet")**			

2. Do your cows

- ◯ Spend all day in the stable
- ◯ Spend the day grazing on the same property as the barn
- ◯ Spend the day grazing on a different property than the barn

3. If you do any of the following when your cattle get sick, what order would you follow (e.g. 1st, 2nd, 3rd, etc.)	1st	2nd	3rd	4th	5th	6th	7th
Treat with antibiotics	◯	◯	◯	◯	◯	◯	◯
Isolate the sick animal(s)	◯	◯	◯	◯	◯	◯	◯
Call a veterinarian	◯	◯	◯	◯	◯	◯	◯
Send samples to the diagnostic laboratory	◯	◯	◯	◯	◯	◯	◯
Slaughter them for meat	◯	◯	◯	◯	◯	◯	◯
On farm culling	◯	◯	◯	◯	◯	◯	◯
Treat with non-antibiotic medications	◯	◯	◯	◯	◯	◯	◯
Other	◯	◯	◯	◯	◯	◯	◯

None of the above

- ◯ OK

Other, please specify

4. Estimate the percentage of your cattle that receive antimicrobials at least once before they go to market (including medicated feed over the last 12 months.

5. Estimate the percentage of your calves that receive antibiotics at least once before they are 6 months old (including medicated feed and medicated milk replacer)

6. Estimate the percentage of your heifers that receive antibiotics at least once after they are 6 months old and before they calve (including medicated feed and medicated milk replacer)

7. What percentage of your animals died of disease over the last 12 months?

Cows (milking and dry)

Calves under 6 months

Heifers

8. Which of the following do you do on your farm (please check all that apply?

Buy new breeding animals from more than one source

- ◯ Yes, always
- ◯ Yes, sometimes
- ◯ No
- ◯ Don't know

Keep cattle enclosed in pastures/pens

- Yes, always
- Yes, sometimes
- No
- Don't know

Use shared/community pastures

- Yes, always
- Yes, sometimes
- No
- Don't know

Keep new animals introduced to the farm separately for at least 30 days

- Yes, always
- Yes, sometimes
- No
- Don't know

Keep sick animals separately

- Yes, always
- Yes, sometimes
- No
- Don't know

Share bulls with other farmers

- Yes, always
- Yes, sometimes
- No
- Don't know

Use purchased semen for artificial insemination

- Yes, always
- Yes, sometimes
- No
- Don't know

Share equipment with other farmers

- ◯ Yes, always
- ◯ Yes, sometimes
- ◯ No
- ◯ Don't know

Clean and disinfect equipment if shared with other farmers

- ◯ Yes, always
- ◯ Yes, sometimes
- ◯ No
- ◯ Don't know

Provide boots and coveralls for visitors

- ◯ Yes, always
- ◯ Yes, sometimes
- ◯ No
- ◯ Don't know

Give colostrum to calves

- ◯ Yes, always
- ◯ Yes, sometimes
- ◯ No
- ◯ Don't know

If give colostrum, what is the average time (hours) after calving that the calf is given colostrum?

....

If give colostrum, how long (minutes) does the calf continually suck for on average?

....

Clean and dry calving boxes before calving

- ◯ Yes, always
- ◯ Yes, sometimes
- ◯ No
- ◯ Don't know

Perform California Mastitis Test on your farm

- ◯ Yes, always
- ◯ Yes, sometimes
- ◯ No
- ◯ Don't know

Wear gloves during milking

- ◯ Yes, always
- ◯ Yes, sometimes
- ◯ No
- ◯ Don't know

Use pre-milking teat dips

- ◯ Yes, always
- ◯ Yes, sometimes
- ◯ No
- ◯ Don't know

Use paper towels or reusable cloth towels to clean and dry teats

- ◯ Yes, always
- ◯ Yes, sometimes
- ◯ No
- ◯ Don't know

Use post-milking teat disinfection

- ◯ Yes, always
- ◯ Yes, sometimes
- ◯ No
- ◯ Don't know

Spritz some milk from each teat before milking into the bucket or putting on the machine

- ◯ Yes, always
- ◯ Yes, sometimes
- ◯ No
- ◯ Don't know

Milk sick/mastitis cows last

- Yes, always
- Yes, sometimes
- No
- Don't know

Track and keep record of cows with mastitis

- Yes, always
- Yes, sometimes
- No
- Don't know

Use calcium supplementation after calving

- Yes, always
- Yes, sometimes
- No
- Don't know

Have other domestic animals in the cattle barn

- Yes, always
- Yes, sometimes
- No
- Don't know

Keep all wild animals out of the cattle barn including birds

- Yes, always
- Yes, sometimes
- No
- Don't know

Prevent wild birds from accessing the feed storage

- Yes, always
- Yes, sometimes
- No
- Don't know

Have wheel dipping/vehicle disinfection at the entrance to the farm

- ◯ Yes, always
- ◯ Yes, sometimes
- ◯ No
- ◯ Don't know

9. Please complete the following table on your herd for the last 12 months

Calves under 6 months

**Disease/ Syndrome**	**Affected (%)**	**Mortality (%)**	**Vaccinated (%)**
**Anaplasmosis**			
**Babesiosis**			
**Brucellosis**			
**Nervous system problems**			
**Footrot**			
**Other lameness**			
**Diarrhea**			
**Hardware**			

**Other intestinal problems**			
**Breathing problems**			
**Other**			

Other Disease/ Syndrome, please specify

Heifers

Disease/ Syndrome	Affected (%)	Mortality (%)	Vaccinated (%)
**Anaplasmosis**			
**Babesiosis**			
**Brucellosis**			
**Nervous system problems**			
**Footrot**			
**Other lameness**			

**Diarrhea**			
**Hardware**			
**Other intestinal problems**			
**Metritis**			
**Other Reproductive problems**			
**Breathing problems**			
**Other**			

Other Disease/ Syndrome, please specify

Cows

Disease/ Syndrome	Affected (%)	Mortality (%)	Vaccinated (%)
**Anaplasmosis**			
**Babesiosis**			

**Brucellosis**			
**Nervous system problems**			
**Footrot**			
**Other lameness**			
**Diarrhea**			
**Hardware**			
**Other intestinal problems**			
**Mastitis**			
**Metritis**			
**Other Reproductive problems**			
**Breathing problems**			

**Other**			

Other Disease/ Syndrome, please specify

9.1 Have you experienced an in increased incidence of any of these diseases since the lockdown due to COVID?

- Yes
- No

If yes, please specify

10. Do you use antibiotics to make healthy animals grow faster/bigger even if the antibiotics are not needed to prevent or treat disease?

- Yes
- No

10.1 If yes, please complete the following questions

1

1.What Antibiotic (use numbered list provided)

2. Stages of Life

- Cows
- Heifers
- Calves

3. How

- Feed
- Water

4. For how many days in a row on average?

5. What percentage of animals per year?

11. Do you use dry cow treatment?

- ◯ Yes
- ◯ No

If yes, what antibiotic?

If yes, what percentage of cows do you treat?

- ◯ <5%
- ◯ 6-25%
- ◯ 26-50%
- ◯ 51-75%
- ◯ 76-99%
- ◯ 100%

12. Please list maximum of 5 antibiotics you've used most frequently for treatment or prevention in the last 12 months including medicated feed

The farmer does not use any antibiotic (to end the survey)

- ◯ OK

the 1st antibiotic

the 2nd antibiotic

the 3rd antibiotic

the 4th antibiotic

the 5th antibiotic

Please complete the following questions for the 1st Antibiotic

2. Reason for Antibiotic (from Disease/Syndrome)

- Anaplasmosis
- Babesiosis
- Brucellosis
- Nervous system problems
- Footrot
- Other lameness
- Diarrhea
- Hardware
- Other intestinal problems
- Mastitis
- Metritis
- Other Reproductive problems
- Breathing problems
- Other

Other, please specify

3. Stage of Life

- Cows
- Heifers
- Calves

4. Aim

- Treatment
- Prevention

5. How

- Feed
- Water
- Injection
- Topical
- Drench
- Bolus
- In the teat

6. For how many days in a row on average?

7. What percentage of animals per year?

8. If this antibiotic doesn't work, what do you do?

- Repeat the treatment with the same antibiotic
- Consult with a veterinarian
- Use a higher dose of the same antibiotic
- Use a different antibiotic
- Do nothing
- Send for slaughter
- Kill and discard the animal
- Slaughter for my own consumption
- Other

which one?

Do you test samples to identify the pathogen?

- Yes
- No

Do you wait for antibiotic susceptibility information from the laboratory?

- Yes
- No

Other, please specify

Please complete the following questions for the 2nd Antibiotic

2. Reason for Antibiotic (from Disease/Syndrome)

- Anaplasmosis
- Babesiosis
- Brucellosis
- Nervous system problems
- Footrot
- Other lameness
- Diarrhea
- Hardware
- Other intestinal problems
- Mastitis
- Metritis
- Other Reproductive problems
- Breathing problems
- Other

Other, please specify

3. Stage of Life

- Cows
- Heifers
- Calves

4. Aim

- Treatment
- Prevention

5. How

- Feed
- Water
- Injection
- Topical
- Drench
- Bolus
- In the teat

6. For how many days in a row on average?

7. What percentage of animals per year?

8. If this antibiotic doesn't work, what do you do?

- Repeat the treatment with the same antibiotic
- Consult with a veterinarian
- Use a higher dose of the same antibiotic
- Use a different antibiotic
- Do nothing
- Send for slaughter
- Kill and discard the animal
- Slaughter for my own consumption
- Other

which one?

Do you test samples to identify the pathogen?

- Yes
- No

Do you wait for antibiotic susceptibility information from the laboratory?

- Yes
- No

Other, please specify

Please complete the following questions for the 3rd Antibiotic

2. Reason for Antibiotic (from Disease/Syndrome)

- Anaplasmosis
- Babesiosis
- Brucellosis
- Nervous system problems
- Footrot
- Other lameness
- Diarrhea
- Hardware
- Other intestinal problems
- Mastitis
- Metritis
- Other Reproductive problems
- Breathing problems
- Other

Other, please specify

3. Stage of Life

- Cows
- Heifers
- Calves

4. Aim

- Treatment
- Prevention

5. How

- Feed
- Water
- Injection
- Topical
- Drench
- Bolus
- In the teat

6. For how many days in a row on average?

7. What percentage of animals per year?

8. If this antibiotic doesn't work, what do you do?

- Repeat the treatment with the same antibiotic
- Consult with a veterinarian
- Use a higher dose of the same antibiotic
- Use a different antibiotic
- Do nothing
- Send for slaughter
- Kill and discard the animal
- Slaughter for my own consumption
- Other

which one?

Do you test samples to identify the pathogen?

- Yes
- No

Do you wait for antibiotic susceptibility information from the laboratory?

- Yes
- No

Other, please specify

Please complete the following questions for the 4th Antibiotic

2. Reason for Antibiotic (from Disease/Syndrome)

- Anaplasmosis
- Babesiosis
- Brucellosis
- Nervous system problems
- Footrot
- Other lameness
- Diarrhea
- Hardware
- Other intestinal problems
- Mastitis
- Metritis
- Other Reproductive problems
- Breathing problems
- Other

Other, please specify

3. Stage of Life

- Cows
- Heifers
- Calves

4. Aim

- Treatment
- Prevention

5. How

- Feed
- Water
- Injection
- Topical
- Drench
- Bolus
- In the teat

6. For how many days in a row on average?

7. What percentage of animals per year?

8. If this antibiotic doesn't work, what do you do?

- Repeat the treatment with the same antibiotic
- Consult with a veterinarian
- Use a higher dose of the same antibiotic
- Use a different antibiotic
- Do nothing
- Send for slaughter
- Kill and discard the animal
- Slaughter for my own consumption
- Other

which one?

Do you test samples to identify the pathogen?

- Yes
- No

Do you wait for antibiotic susceptibility information from the laboratory?

- Yes
- No

Other, please specify

Please complete the following questions for the 5th Antibiotic

2. Reason for Antibiotic (from Disease/Syndrome)

- Anaplasmosis
- Babesiosis
- Brucellosis
- Nervous system problems
- Footrot
- Other lameness
- Diarrhea
- Hardware
- Other intestinal problems
- Mastitis
- Metritis
- Other Reproductive problems
- Breathing problems
- Other

Other, please specify

3. Stage of Life

- Cows
- Heifers
- Calves

4. Aim

- Treatment
- Prevention

5. How

- Feed
- Water
- Injection
- Topical
- Drench
- Bolus
- In the teat

6. For how many days in a row on average?

7. What percentage of animals per year?

8. If this antibiotic doesn't work, what do you do?

- Repeat the treatment with the same antibiotic
- Consult with a veterinarian
- Use a higher dose of the same antibiotic
- Use a different antibiotic
- Do nothing
- Send for slaughter
- Kill and discard the animal
- Slaughter for my own consumption
- Other

which one?

Do you test samples to identify the pathogen?

- Yes
- No

Do you wait for antibiotic susceptibility information from the laboratory?

- ○ Yes
- ○ No

Other, please specify

____

13. Did the participant refuse to answer any of these questions?

- ○ Yes
- ○ No

If yes, list the number(s) of these questions

____

If yes, check all reasons that participant gave for this refusal

- ☐ Concerned about researchers knowing this information
- ☐ Concerned about government knowing this information
- ☐ Concerned about other farmers knowing this information
- ☐ Other

Other, please specify

____

Comments

____

Small Ruminant _Farmer Survey Part 2_FAO AMU Survey_Balkan

Interviewer ID *

Interview Date *

yyyy-mm-dd

Farmer ID *

Choose the country *

- North Macedonia
- Bosnia and Herzegovina
- Serbia
- Albania
- Montenegro
- Kosovo

1. If you have sheep, are your sheep raised for

- Milk
- Meat
- Both milk and meat
- Wool
- Both meat and wool
- Meat, milk and wool

2. If you have goats, are your goats raised for

- Meat
- Milk
- Both meat and milk

3. Over the last 12 months, what is the minimum number and the maximum number of each type of animal that you have had on your farm?

Stages of Life	Minimum number in last 12 months	Maximum number in last 12 months	Average number of animals on the farm over the last 12 months
Ewes			
Rams			
Lambs			
Does			
Bucks			
Kids			

4. If you do any of the following when your sheep or goats get sick, what order would you follow (e.g. 1st, 2nd, 3rd, etc.)	1st	2nd	3rd	4th	5th	6th	7th
Treat with antibiotics	○	○	○	○	○	○	○
Isolate the sick animal(s)	○	○	○	○	○	○	○
Call a veterinarian	○	○	○	○	○	○	○
Send samples to the diagnostic laboratory	○	○	○	○	○	○	○
Slaughter them for meat	○	○	○	○	○	○	○
On farm culling	○	○	○	○	○	○	○

Treat with non-antibiotic medications	◯	◯	◯	◯	◯	◯	◯
Other	◯	◯	◯	◯	◯	◯	◯

None of the above

◯ OK

Other, please specify

5. Estimate the percentage of your cattle that receive antibiotics at least once during their lactation (including medicated feed)

6. What percentage of your animals died of disease over the last 12 months?

Ewes

Lambs

Does

Kids

7. Which of the following do you do on your farm (please check all that apply?

Buy new breeding animals from more than one source

◯ Yes, always

◯ Yes, sometimes

◯ No

◯ Don't know

Keep new animals introduced onto the farm separately for at least 30 days

- ◯ Yes, always
- ◯ Yes, sometimes
- ◯ No
- ◯ Don't know

Share rams/bucks with other farmers

- ◯ Yes, always
- ◯ Yes, sometimes
- ◯ No
- ◯ Don't know

Move your sheep/goats between summer and winter pastures

- ◯ Yes, always
- ◯ Yes, sometimes
- ◯ No
- ◯ Don't know

Use shared/community pastures

- ◯ Yes, always
- ◯ Yes, sometimes
- ◯ No
- ◯ Don't know

Share equipment with other farmers

- ◯ Yes, always
- ◯ Yes, sometimes
- ◯ No
- ◯ Don't know

Clean and disinfect equipment if shared with other farmers

- ◯ Yes, always
- ◯ Yes, sometimes
- ◯ No
- ◯ Don't know

Keep sick animals separately

- Yes, always
- Yes, sometimes
- No
- Don't know

Keep sheep/goats enclosed in pastures/ pens

- Yes, always
- Yes, sometimes
- No
- Don't know

Provide boots for visitors

- Yes, always
- Yes, sometimes
- No
- Don't know

Register visitors

- Yes, always
- Yes, sometimes
- No
- Don't know

Have rodent and pest control measures in the barn(s)

- Yes, always
- Yes, sometimes
- No
- Don't know

Have other domestic animals in the sheep/goat barn

- Yes, always
- Yes, sometimes
- No
- Don't know

Keep all wild animals out of the sheep/goat barn

- Yes, always
- Yes, sometimes
- No
- Don't know

Prevent wild birds from accessing the feed storage

- Yes, always
- Yes, sometimes
- No
- Don't know

Have wheel dipping/vehicle disinfection at the entrance to the farm

- Yes, always
- Yes, sometimes
- No
- Don't know

8. Please complete the following table on your herd for the last 12 months

Lambs

**Disease/ Syndrome**	**Affected (%)**	**Mortality (%)**	**Vaccinated (%)**
**Breathing problems**			
**Nervous System problems**			
**Brucellosis**			

**Footrot**			
**Skin disease**			
**Diarrhea**			
**Other intestinal problems**			
**Lameness**			
**Sudden death**			
**other**			

Other Disease/ Syndrome, please specify

Kids

**Disease/ Syndrome**	**Affected (%)**	**Mortality (%)**	**Vaccinated (%)**
**Breathing problems**			
**Nervous System problems**			

**Brucellosis**			
**Footrot**			
**Skin disease**			
**Diarrhea**			
**Other intestinal problems**			
**Lameness**			
**Sudden death**			
**Other**			

Other, please specify

Adult Ewes/Does

**Disease/ Syndrome**	**Affected (%)**	**Mortality (%)**	**Vaccinated (%)**
**Breathing problems**			

**Nervous System problems**			
**Brucellosis**			
**Footrot**			
**Skin disease**			
**Diarrhea**			
**Other intestinal problems**			
**Mastitis**			
**Other reproductive problems**			
**Lameness**			
**Sudden death**			
**Other**			

Other, please specify

8.1 Have you experienced an in increased incidence of any of these diseases since the lockdown due to COVID?

- ◯ Yes
- ◯ No

If yes, please specify

9. Do you use antibiotics to make healthy animals grow faster/bigger/give more milk even if the antibiotics are not needed to prevent or treat disease?

- ◯ Yes
- ◯ No

9.1 If yes, please complete the following questions

1

1.What Antibiotic (use numbered list provided)

2. Stages of Life

- ◯ Ewes
- ◯ Does
- ◯ Lambs
- ◯ Kids

3. How

- ◯ Feed
- ◯ Water

4. For how many days in a row on average?

5. What percentage of animals per year?

10. Please list maximum of 5 antibiotics you've used most frequently for treatment or prevention in the last 12 months including medicated feed

The farmer does not use any antibiotic (to end the survey)

- ○ OK

the 1st antibiotic

the 2nd antibiotic

the 3rd antibiotic

the 4th antibiotic

the 5th antibiotic

Please complete the following questions for the 1st Antibiotic

2. Reason for Antibiotic (from Disease/Syndrome)

- Breathing problems
- Nervous System problems
- Brucellosis
- Footrot
- Skin disease
- Diarrhea
- Other intestinal problems
- Mastitis
- Other reproductive problems
- Lameness
- Sudden death
- Other

Other, please specify

3. Stage of Life

- Ewes
- Does
- Lambs
- Kids

4. Aim

- Treatment
- Prevention

5. How

- Feed
- Water
- Injection
- Topical
- Drench
- Bolus
- In the teat

6. For how many days in a row on average?

7. What percentage of animals per year?

8. If this antibiotic doesn't work, what do you do?

- Repeat the treatment with the same antibiotic
- Consult with a veterinarian
- Use a higher dose of the same antibiotic
- Use a different antibiotic
- Do nothing
- Send for slaughter
- Kill and discard the animal
- Slaughter for my own consumption
- Other

which one?

Do you test samples to identify the pathogen?

- Yes
- No

Do you wait for antibiotic susceptibility information from the laboratory?

- Yes
- No

Other, please specify

Please complete the following questions for the 2nd Antibiotic

2. Reason for Antibiotic (from Disease/Syndrome)

- Breathing problems
- Nervous System problems
- Brucellosis
- Footrot
- Skin disease
- Diarrhea
- Other intestinal problems
- Mastitis
- Other reproductive problems
- Lameness
- Sudden death
- Other

Other, please specify

3. Stage of Life

- Ewes
- Does
- Lambs
- Kids

4. Aim

- Treatment
- Prevention

5. How

- Feed
- Water
- Injection
- Topical
- Drench
- Bolus
- In the teat

6. For how many days in a row on average?

7. What percentage of animals per year?

8. If this antibiotic doesn't work, what do you do?

- Repeat the treatment with the same antibiotic
- Consult with a veterinarian
- Use a higher dose of the same antibiotic
- Use a different antibiotic
- Do nothing
- Send for slaughter
- Kill and discard the animal
- Slaughter for my own consumption
- Other

which one?

Do you test samples to identify the pathogen?

- Yes
- No

Do you wait for antibiotic susceptibility information from the laboratory?

- Yes
- No

Other, please specify

Please complete the following questions for the 3rd Antibiotic

2. Reason for Antibiotic (from Disease/Syndrome)

- Breathing problems
- Nervous System problems
- Brucellosis
- Footrot
- Skin disease
- Diarrhea
- Other intestinal problems
- Mastitis
- Other reproductive problems
- Lameness
- Sudden death
- Other

Other, please specify

3. Stage of Life

- Ewes
- Does
- Lambs
- Kids

4. Aim

- Treatment
- Prevention

5. How

- Feed
- Water
- Injection
- Topical
- Drench
- Bolus
- In the teat

6. For how many days in a row on average?

7. What percentage of animals per year?

8. If this antibiotic doesn't work, what do you do?

- Repeat the treatment with the same antibiotic
- Consult with a veterinarian
- Use a higher dose of the same antibiotic
- Use a different antibiotic
- Do nothing
- Send for slaughter
- Kill and discard the animal
- Slaughter for my own consumption
- Other

which one?

Do you test samples to identify the pathogen?

- Yes
- No

Do you wait for antibiotic susceptibility information from the laboratory?

- Yes
- No

Other, please specify

Please complete the following questions for the 4th Antibiotic

2. Reason for Antibiotic (from Disease/Syndrome)

- Breathing problems
- Nervous System problems
- Brucellosis
- Footrot
- Skin disease
- Diarrhea
- Other intestinal problems
- Mastitis
- Other reproductive problems
- Lameness
- Sudden death
- Other

Other, please specify

3. Stage of Life

- Ewes
- Does
- Lambs
- Kids

4. Aim

- Treatment
- Prevention

5. How

- Feed
- Water
- Injection
- Topical
- Drench
- Bolus
- In the teat

6. For how many days in a row on average?

7. What percentage of animals per year?

8. If this antibiotic doesn't work, what do you do?

- Repeat the treatment with the same antibiotic
- Consult with a veterinarian
- Use a higher dose of the same antibiotic
- Use a different antibiotic
- Do nothing
- Send for slaughter
- Kill and discard the animal
- Slaughter for my own consumption
- Other

which one?

Do you test samples to identify the pathogen?

- Yes
- No

Do you wait for antibiotic susceptibility information from the laboratory?

- Yes
- No

Other, please specify

Please complete the following questions for the 5th Antibiotic

2. Reason for Antibiotic (from Disease/Syndrome)

- Breathing problems
- Nervous System problems
- Brucellosis
- Footrot
- Skin disease
- Diarrhea
- Other intestinal problems
- Mastitis
- Other reproductive problems
- Lameness
- Sudden death
- Other

Other, please specify

3. Stage of Life

- Ewes
- Does
- Lambs
- Kids

4. Aim

- Treatment
- Prevention

5. How

- Feed
- Water
- Injection
- Topical
- Drench
- Bolus
- In the teat

6. For how many days in a row on average?

7. What percentage of animals per year?

8. If this antibiotic doesn't work, what do you do?

- Repeat the treatment with the same antibiotic
- Consult with a veterinarian
- Use a higher dose of the same antibiotic
- Use a different antibiotic
- Do nothing
- Send for slaughter
- Kill and discard the animal
- Slaughter for my own consumption
- Other

which one?

Do you test samples to identify the pathogen?

- Yes
- No

Do you wait for antibiotic susceptibility information from the laboratory?

- Yes
- No

Other, please specify

11. Did the participant refuse to answer any of these questions?

- Yes
- No

If yes, list the number(s) of these questions

If yes, check all reasons that participant gave for this refusal

- Concerned about researchers knowing this information
- Concerned about government knowing this information
- Concerned about other farmers knowing this information
- Other

Other, please specify

Comments

Pigs _Farmer Survey Part 2_FAO AMU Survey

Interviewer ID *

Interview Date *

yyyy-mm-dd

Farmer ID *

Choose the country *

- North Macedonia
- Bosnia and Herzegovina
- Serbia
- Albania
- Montenegro
- Kosovo

1. Over the last 12 months, what is the minimum number and the maximum number of each type of animal that you have had on your farm?

Stages of Life	Minimum number in last 12 months	Maximum number in last 12 months	Average number of animals on the farm over the last 12 months
Sows			
Boars			
Piglets (nursing the sow)			

**Fattening pigs**			

2. If you do any of the following when your pigs get sick, what order would you follow (eg. 1st, 2nd, 3rd, etc)	1st	2nd	3rd	4th	5th	6th
Treat with antibiotics	○	○	○	○	○	○
Isolate the sick pig(s)	○	○	○	○	○	○
Call a veterinarian	○	○	○	○	○	○
Send samples to the diagnostic laboratory	○	○	○	○	○	○
Slaughter them for meat	○	○	○	○	○	○
Treat with non-antibiotic medications	○	○	○	○	○	○
Other	○	○	○	○	○	○

None of the above

○ OK

Other, please specify

3. Estimate the percentage of your fattening pigs that receive antibiotics at least once between birth and market (including medicated feed)

4. What percentage of pigs died of disease over the last 12 months

Sows

Piglets (nursing the sow)

Fattening pigs

5. Which of the following do you do on your farm (please check all that apply?

Buy new breeding animals from more than one source

- Yes, always
- Yes, sometimes
- No
- Don't know

Keep new animals introduced onto the farm separately for at least 30 days

- Yes, always
- Yes, sometimes
- No
- Don't know

Share boars with other farmers

- Yes, always
- Yes, sometimes
- No
- Don't know

Use purchased semen for artificial insemination

- Yes, always
- Yes, sometimes
- No
- Don't know

Keep weaned pigs separated from adult and nursing pigs

- Yes, always
- Yes, sometimes
- No
- Don't know

Share equipment with other farmers

- Yes, always
- Yes, sometimes
- No
- Don't know

Clean and disinfect equipment if shared with other farmers

- Yes, always
- Yes, sometimes
- No
- Don't know

Keep sick animals separately

- Yes, always
- Yes, sometimes
- No
- Don't know

Provide coveralls for visitors

- Yes, always
- Yes, sometimes
- No
- Don't know

Provide boots for visitors

- Yes, always
- Yes, sometimes
- No
- Don't know

Have wheel dipping/vehicle disinfection at the entrance to the farm

- Yes, always
- Yes, sometimes
- No
- Don't know

Register visitors

- Yes, always
- Yes, sometimes
- No
- Don't know

Have a rodent and pest control program in the barn(s)

- Yes, always
- Yes, sometimes
- No
- Don't know

Have other domestic animals in the pig barn

- Yes, always
- Yes, sometimes
- No
- Don't know

Keep all wild animals out of the pig barn including birds

- Yes, always
- Yes, sometimes
- No
- Don't know

Prevent wild birds from accessing the feed storage

- Yes, always
- Yes, sometimes
- No
- Don't know

6. Please complete the following table on your herd for the last 12 months

Piglets (nursing the sow)

Disease/ Syndrome	Affected (%)	Mortality (%)	Vaccinated (%)
Breathing problems			
Nervous System problems			
Swine Dysentery			
Porcine Reproductive and Respiratory Syndrome (PRRS)			
Skin disease			
Diarrhea			
Other intestinal problems			
Lameness			
African Swine Fever			
Sudden death			

**other**			

Other Disease/ Syndrome, please specify

Fattening Pigs

**Disease/ Syndrome**	**Affected (%)**	**Mortality (%)**	**Vaccinated (%)**
**Breathing problems**			
**Nervous System problems**			
**Swine Dysentery**			
**Porcine Reproductive and Respiratory Syndrome (PRRS)**			
**Skin disease**			
**Diarrhea**			
**Other intestinal problems**			

**Lameness**			
**African Swine Fever**			
**Sudden death**			
**Other**			

Other, please specify

Adult sows/ boars

**Disease/ Syndrome**	**Affected (%)**	**Mortality (%)**	**Vaccinated (%)**
**Breathing problems**			
**Nervous System problems**			
**Swine Dysentery**			
**Porcine Reproductive and Respiratory Syndrome (PRRS)**			

**Skin disease**			
**Diarrhea (PRRS)**			
**Other intestinal problems**			
**Not milking**			
**Reproductive problems**			
**Lameness**			
**African Swine Fever**			
**Sudden death**			
**Other**			

Other, please specify

6.1 Have you experienced an in increased incidence of any of these diseases since the lockdown due to COVID?

- ◯ Yes
- ◯ No

If yes, please specify

7. Do you use antibiotics to make healthy pigs grow faster/bigger even if the antibiotics are not needed to prevent or treat disease?

- Yes
- No

7.1 If yes, please complete the following questions

1

1.What Antibiotic (use numbered list provided)

2. Stages of Life

- Sows/boars
- Piglets
- Fattening pigs

3. How

- Feed
- Water

4. For how many days in a row on average?

5. What percentage of animals per year?

8. Please list maximum of 5 antibiotics you've used most frequently for treatment or prevention in the last 12 months including medicated feed

The farmer does not use any antibiotic (to end the survey)

- OK

the 1st antibiotic

the 2nd antibiotic
the 3rd antibiotic
the 4th antibiotic
the 5th antibiotic

Please complete the following questions for the 1st Antibiotic

2. Reason for Antibiotic (from Disease/Syndrome)

- Breathing problems
- Nervous System problems
- Swine Dysentery
- Porcine Reproductive and Respiratory Syndrome (PRRS)
- Skin disease
- Diarrhea
- Other intestinal problems
- Not milking
- Reproductive problems
- Lameness
- Sudden death
- African Swine Fever
- Other

Other, please specify

3. Stage of Life

- Sows/boars
- Piglets
- Fattening pigs

4. Aim

- Treatment
- Prevention

5. How

- Feed
- Water
- Injection
- Bolus
- Drench
- Topical

6. For how many days in a row on average?

7. What percentage of animals per year?

8. If this antibiotic doesn't work, what do you do?

- Repeat the treatment with the same antibiotic
- Consult with a veterinarian
- Use a higher dose of the same antibiotic
- Use a different antibiotic
- Do nothing
- Send for slaughter
- Kill and discard the animal
- Slaughter for my own consumption
- Other

which one?

Do you test samples to identify the pathogen?

- Yes
- No

Do you wait for antibiotic susceptibility information from the laboratory?

- Yes
- No

Other, please specify

Please complete the following questions for the 2nd Antibiotic

2. Reason for Antibiotic (from Disease/Syndrome)

- Breathing problems
- Nervous System problems
- Swine Dysentery
- Porcine Reproductive and Respiratory Syndrome (PRRS)
- Skin disease
- Diarrhea
- Other intestinal problems
- Not milking
- Reproductive problems
- Lameness
- Sudden death
- African Swine Fever
- Other

Other, please specify

3. Stage of Life

- Sows/boars
- Piglets
- Fattening pigs

4. Aim

- Treatment
- Prevention

5. How

- Feed
- Water
- Injection
- Bolus
- Drench
- Topical

6. For how many days in a row on average?

7. What percentage of animals per year?

8. If this antibiotic doesn't work, what do you do?

- Repeat the treatment with the same antibiotic
- Consult with a veterinarian
- Use a higher dose of the same antibiotic
- Use a different antibiotic
- Do nothing
- Send for slaughter
- Kill and discard the animal
- Slaughter for my own consumption
- Other

which one?

Do you test samples to identify the pathogen?

- Yes
- No

Do you wait for antibiotic susceptibility information from the laboratory?

- Yes
- No

Other, please specify

Please complete the following questions for the 3rd Antibiotic

2. Reason for Antibiotic (from Disease/Syndrome)

- Breathing problems
- Nervous System problems
- Swine Dysentery
- Porcine Reproductive and Respiratory Syndrome (PRRS)
- Skin disease
- Diarrhea
- Other intestinal problems
- Not milking
- Reproductive problems
- Lameness
- Sudden death
- African Swine Fever
- Other

Other, please specify

3. Stage of Life

- Sows/boars
- Piglets
- Fattening pigs

4. Aim

- Treatment
- Prevention

5. How

- Feed
- Water
- Injection
- Bolus
- Drench
- Topical

6. For how many days in a row on average?

7. What percentage of animals per year?

8. If this antibiotic doesn't work, what do you do?

- Repeat the treatment with the same antibiotic
- Consult with a veterinarian
- Use a higher dose of the same antibiotic
- Use a different antibiotic
- Do nothing
- Send for slaughter
- Kill and discard the animal
- Slaughter for my own consumption
- Other

which one?

Do you test samples to identify the pathogen?

- Yes
- No

Do you wait for antibiotic susceptibility information from the laboratory?

- Yes
- No

Other, please specify

Please complete the following questions for the 4th Antibiotic

2. Reason for Antibiotic (from Disease/Syndrome)

- Breathing problems
- Nervous System problems
- Swine Dysentery
- Porcine Reproductive and Respiratory Syndrome (PRRS)
- Skin disease
- Diarrhea
- Other intestinal problems
- Not milking
- Reproductive problems
- Lameness
- Sudden death
- African Swine Fever
- Other

Other, please specify

3. Stage of Life

- Sows/boars
- Piglets
- Fattening pigs

4. Aim

- Treatment
- Prevention

5. How

- Feed
- Water
- Injection
- Bolus
- Drench
- Topical

6. For how many days in a row on average?

7. What percentage of animals per year?

8. If this antibiotic doesn't work, what do you do?

- Repeat the treatment with the same antibiotic
- Consult with a veterinarian
- Use a higher dose of the same antibiotic
- Use a different antibiotic
- Do nothing
- Send for slaughter
- Kill and discard the animal
- Slaughter for my own consumption
- Other

which one?

Do you test samples to identify the pathogen?

- Yes
- No

Do you wait for antibiotic susceptibility information from the laboratory?

- Yes
- No

Other, please specify

Please complete the following questions for the 5th Antibiotic

2. Reason for Antibiotic (from Disease/Syndrome)

- Breathing problems
- Nervous System problems
- Swine Dysentery
- Porcine Reproductive and Respiratory Syndrome (PRRS)
- Skin disease
- Diarrhea
- Other intestinal problems
- Not milking
- Reproductive problems
- Lameness
- Sudden death
- African Swine Fever
- Other

Other, please specify

3. Stage of Life

- Sows/boars
- Piglets
- Fattening pigs

4. Aim

- Treatment
- Prevention

5. How

- Feed
- Water
- Injection
- Bolus
- Drench
- Topical

6. For how many days in a row on average?

7. What percentage of animals per year?

8. If this antibiotic doesn't work, what do you do?

- Repeat the treatment with the same antibiotic
- Consult with a veterinarian
- Use a higher dose of the same antibiotic
- Use a different antibiotic
- Do nothing
- Send for slaughter
- Kill and discard the animal
- Slaughter for my own consumption
- Other

which one?

Do you test samples to identify the pathogen?

- Yes
- No

Do you wait for antibiotic susceptibility information from the laboratory?

- ◯ Yes
- ◯ No

Other, please specify

....

9. Did the participant refuse to answer any of these questions?

- ◯ Yes
- ◯ No

If yes, list the number(s) of these questions

....

If yes, check all reasons that participant gave for this refusal

- ☐ Concerned about researchers knowing this information
- ☐ Concerned about government knowing this information
- ☐ Concerned about other farmers knowing this information
- ☐ Other

Other, please specify

....

Comments

....

Chicken _Farmer Survey Part 2_FAO AMU Survey

Interviewer ID *

Interview Date *

yyyy-mm-dd

Farmer ID *

Choose the country *

- ◯ North Macedonia
- ◯ Bosnia and Herzegovina
- ◯ Serbia
- ◯ Albania
- ◯ Montenegro
- ◯ Kosovo

1. Are your chickens raised for:

- ◯ Chicken meat
- ◯ Eggs
- ◯ Both meat and eggs

2. Where do you obtain your chicks/ hatching eggs

- ☐ Raise your own
- ☐ Purchase

If purchase, please indicate source

3. What is the minimum number and the maximum number of each type of chicken that you have had on your farm?

**Stages of Life**	**Minimum number in last 12 months**	**Maximum number in last 12 months**	**Average number of chickens on the farm over the last 12 months**
**Chicks under 1 week old**			
**Broiler Chickens**			
**Layers**			

4. If you do any of the following when your birds get sick, what order would you follow (e.g. 1st, 2nd, 3rd, etc.)	1st	2nd	3rd	4th	5th	6th
Treat with antibiotics	○	○	○	○	○	○
Isolate the sick chicken(s)	○	○	○	○	○	○
Call a veterinarian	○	○	○	○	○	○
Send samples to the diagnostic laboratory	○	○	○	○	○	○
Slaughter them for meat	○	○	○	○	○	○
Treat with non-antibiotic medications	○	○	○	○	○	○
Other	○	○	○	○	○	○

None of the above

○ OK

Other, please specify

5. Estimate the percentage of your chickens that receive antibiotics at least once between birth and market (including medicated feed)

6. What percentage of your chickens died of disease over the last 12 months?

Chicks under 1 week old

Broiler Chickens

Layers

7. Which of the following do you do on your farm (please check all that apply)?

Buy eggs/chicks from more than one source

- Yes, always
- Yes, sometimes
- No
- Don't know

The litter is removed between batches of birds

- Yes, always
- Yes, sometimes
- No
- Don't know

The barn is disinfected between batches of birds

- Yes, always
- Yes, sometimes
- No
- Don't know

There is a footbath at the entrance to each barn

- ◯ Yes, always
- ◯ Yes, sometimes
- ◯ No
- ◯ Don't know

Have wheel dipping/vehicle disinfection at the entrance to the farm

- ◯ Yes, always
- ◯ Yes, sometimes
- ◯ No
- ◯ Don't know

All of the birds are emptied out of the barn before new birds are put in, i.e. all in/all out

- ◯ Yes, always
- ◯ Yes, sometimes
- ◯ No
- ◯ Don't know

There are at least 14 days where the barn is empty between flocks

- ◯ Yes, always
- ◯ Yes, sometimes
- ◯ No
- ◯ Don't know

Keep age groups separate

- ◯ Yes, always
- ◯ Yes, sometimes
- ◯ No
- ◯ Don't know

Share equipment with other farmers

- ◯ Yes, always
- ◯ Yes, sometimes
- ◯ No
- ◯ Don't know

Clean and disinfect equipment if shared with other farmers

- Yes, always
- Yes, sometimes
- No
- Don't know

Give visitors overalls and boots

- Yes, always
- Yes, sometimes
- No
- Don't know

Have rodent and pest control measures in the barn(s)

- Yes, always
- Yes, sometimes
- No
- Don't know

Have measures to keep litter in a relatively dry condition

- Yes, always
- Yes, sometimes
- No
- Don't know

Provide good ventilation

- Yes, always
- Yes, sometimes
- No
- Don't know

Have other animals in the poultry house

- Yes, always
- Yes, sometimes
- No
- Don't know

Prevent wild birds from accessing the feed storage

- ◯ Yes, always
- ◯ Yes, sometimes
- ◯ No
- ◯ Don't know

8. Please complete the following table on your birds for the last 12 months

Chicks

Disease/ Syndrome	Affected (%)	Mortality (%)	Vaccinated (%)
**Breathing problems**			
**Intestinal problems**			
**Skin/ Feather problems**			
**Lameness**			
**Injuries**			
**Blindness**			
**Nervous system**			

**other**			

Other Disease/ Syndrome, please specify

Broiler Chickens

Disease/ Syndrome	Affected (%)	Mortality (%)	Vaccinated (%)
**Breathing problems**			
**Intestinal problems**			
**Skin/ Feather problems**			
**Lameness**			
**Injuries**			
**Blindness**			
**Nervous system**			
**Other**			

Other, please specify

Layers

Disease/ Syndrome	Affected (%)	Mortality (%)	Vaccinated (%)
Breathing problems			
Intestinal problems			
Skin/ Feather problems			
Lameness			
Injuries			
Blindness			
Nervous system			
Not laying			
Other			

Other, please specify

8.1 Have you experienced an in increased incidence of any of these diseases since the lockdown due to COVID?

- Yes
- No

If yes, please specify

9. Do you use antibiotics to make healthy birds grow faster/bigger or lay more eggs even if the antibiotics are not needed to prevent or treat disease

- Yes
- No

9.1 If yes, please complete the following questions

1

1.What Antibiotic (use numbered list provided)

2. Stages of Life

- Chicks
- Broilers
- Layers

3. How

- Feed
- Water

4. For how many days in a row on average?

5. What percentage of birds per year?

10. Please list maximum of 5 antibiotics you've used most frequently for treatment or prevention in the last 12 months including medicated feed

The farmer does not use any antibiotic (to end the survey)

- OK

the 1st antibiotic

the 2nd antibiotic

the 3rd antibiotic

the 4th antibiotic

the 5th antibiotic

Please complete the following questions for the 1st Antibiotic

2. Reason for Antibiotic (from Disease/Syndrome)

- Breathing problems
- Intestinal problems
- Skin/ Feather problems
- Lameness
- Injuries
- Blindness
- Nervous system
- Not laying
- Other

Other, please specify

3. Stage of Life

- Chicks
- Broilers
- Layers

4. Aim

- Treatment
- Prevention

5. How

- Feed
- Water
- Injection
- Topical

6. For how many days in a row on average?

7. What percentage of birds per year?

8. If this antibiotic doesn't work, what do you do?

- Repeat the treatment with the same antibiotic
- Consult with a veterinarian
- Use a higher dose of the same antibiotic
- Use a different antibiotic
- Do nothing
- Send for slaughter
- Kill and discard the animal
- Slaughter for my own consumption
- Other

which one?

Do you test samples to identify the pathogen?

- Yes
- No

Do you wait for antibiotic susceptibility information from the laboratory?

- Yes
- No

Other, please specify

Please complete the following questions for the 2nd Antibiotic

2. Reason for Antibiotic (from Disease/Syndrome)

- Breathing problems
- Intestinal problems
- Skin/ Feather problems
- Lameness
- Injuries
- Blindness
- Nervous system
- Not laying
- Other

Other, please specify

3. Stage of Life

- Chicks
- Broilers
- Layers

4. Aim

- Treatment
- Prevention

5. How

- Feed
- Water
- Injection
- Topical

6. For how many days in a row on average?

7. What percentage of birds per year?

8. If this antibiotic doesn't work, what do you do?

- Repeat the treatment with the same antibiotic
- Consult with a veterinarian
- Use a higher dose of the same antibiotic
- Use a different antibiotic
- Do nothing
- Send for slaughter
- Kill and discard the animal
- Slaughter for my own consumption
- Other

which one?

Do you test samples to identify the pathogen?

- Yes
- No

Do you wait for antibiotic susceptibility information from the laboratory?

- Yes
- No

Other, please specify

Please complete the following questions for the 3rd Antibiotic

2. Reason for Antibiotic (from Disease/Syndrome)

- Breathing problems
- Intestinal problems
- Skin/ Feather problems
- Lameness
- Injuries
- Blindness
- Nervous system
- Not laying
- Other

Other, please specify

3. Stage of Life

- Chicks
- Broilers
- Layers

4. Aim

- Treatment
- Prevention

5. How

- Feed
- Water
- Injection
- Topical

6. For how many days in a row on average?

7. What percentage of birds per year?

8. If this antibiotic doesn't work, what do you do?

- Repeat the treatment with the same antibiotic
- Consult with a veterinarian
- Use a higher dose of the same antibiotic
- Use a different antibiotic
- Do nothing
- Send for slaughter
- Kill and discard the animal
- Slaughter for my own consumption
- Other

which one?

Do you test samples to identify the pathogen?

- Yes
- No

Do you wait for antibiotic susceptibility information from the laboratory?

- Yes
- No

Other, please specify

Please complete the following questions for the 4th Antibiotic

2. Reason for Antibiotic (from Disease/Syndrome)

- Breathing problems
- Intestinal problems
- Skin/ Feather problems
- Lameness
- Injuries
- Blindness
- Nervous system
- Not laying
- Other

Other, please specify

3. Stage of Life

- Chicks
- Broilers
- Layers

4. Aim

- Treatment
- Prevention

5. How

- Feed
- Water
- Injection
- Topical

6. For how many days in a row on average?

7. What percentage of birds per year?

8. If this antibiotic doesn't work, what do you do?

- Repeat the treatment with the same antibiotic
- Consult with a veterinarian
- Use a higher dose of the same antibiotic
- Use a different antibiotic
- Do nothing
- Send for slaughter
- Kill and discard the animal
- Slaughter for my own consumption
- Other

which one?

Do you test samples to identify the pathogen?

- Yes
- No

Do you wait for antibiotic susceptibility information from the laboratory?

- Yes
- No

Other, please specify

Please complete the following questions for the 5th Antibiotic

2. Reason for Antibiotic (from Disease/Syndrome)

- Breathing problems
- Intestinal problems
- Skin/ Feather problems
- Lameness
- Injuries
- Blindness
- Nervous system
- Not laying
- Other

Other, please specify

3. Stage of Life

- Chicks
- Broilers
- Layers

4. Aim

- Treatment
- Prevention

5. How

- Feed
- Water
- Injection
- Topical

6. For how many days in a row on average?

7. What percentage of birds per year?

8. If this antibiotic doesn't work, what do you do?

- Repeat the treatment with the same antibiotic
- Consult with a veterinarian
- Use a higher dose of the same antibiotic
- Use a different antibiotic
- Do nothing
- Send for slaughter
- Kill and discard the animal
- Slaughter for my own consumption
- Other

which one?

Do you test samples to identify the pathogen?

- Yes
- No

Do you wait for antibiotic susceptibility information from the laboratory?

- Yes
- No

Other, please specify

11. Did the participant refuse to answer any of these questions?

- Yes
- No

If yes, list the number(s) of these questions

If yes, check all reasons that participant gave for this refusal

- Concerned about researchers knowing this information
- Concerned about government knowing this information
- Concerned about other farmers knowing this information
- Other

Other, please specify

Comments

Bees _Farmer Survey Part 2_FAO AMU Survey

Interviewer ID *

Interview Date *

yyyy-mm-dd

Farmer ID *

Choose the country *

- ◯ North Macedonia
- ◯ Bosnia and Herzegovina
- ◯ Serbia
- ◯ Albania
- ◯ Montenegro
- ◯ Kosovo

1. Are your bees raised for:

- ☐ Pollination
- ☐ Honey
- ☐ Both pollination and honey
- ☐ Royal jelly
- ☐ Wax
- ☐ Pollen
- ☐ Propolis
- ☐ Queen bees
- ☐ Swarms

2. Where do you obtain your bees

- [ ] Raise your own
- [ ] Purchase

If purchase, please indicate source

If purchase, please indicate geographical origin of bees

3. Do you move your bees to different locations or do they remain on your farm?

- ○ On your farm only
- ○ Move once a year
- ○ Move twice a year
- ○ Move more than twice a year

4. Over the last 12 months, what is the minimum number and the maximum number of each type of hive that you have had on your farm?

Type of hives	Minimum number in last 12 months	Maximum number in last 12 months	Average number of bees on the farm over the last 12 months
**Nucleus hives (Beginner hives) /Swarms**			
**Production hives (colonies)**			

5. If you do any of the following when your bees get sick, what order would you follow (e.g. 1st, 2nd, 3rd, etc.)	1st	2nd	3rd	4th	5th	6th
Treat with antibiotics	○	○	○	○	○	○
Isolate the sick hive	○	○	○	○	○	○
Call a veterinarian	○	○	○	○	○	○
Send samples to the diagnostic laboratory	○	○	○	○	○	○
Eradicate the hive	○	○	○	○	○	○
Treat with non-antibiotic medications	○	○	○	○	○	○
Other	○	○	○	○	○	○

None of the above

- ○ OK

Other, please specify

6. Estimate the percentage of your bees that receive antibiotics at least once (including medicated syrup) in the last year

7. Which of the following do you do on your farm (please check all that apply?

Obtain your bees from more than one source

- ○ Yes, always
- ○ Yes, sometimes
- ○ No
- ○ Don't know

Completely clean bee-hive before introducing a new colony/swarm

- Yes, always
- Yes, sometimes
- No
- Don't know

Keep affected hives in separate locations (quarantine apiary) at least 30 days

- Yes, always
- Yes, sometimes
- No
- Don't know

Use removable frames

- Yes, always
- Yes, sometimes
- No
- Don't know

Share equipment with other farmers

- Yes, always
- Yes, sometimes
- No
- Don't know

Clean and disinfect equipment if shared with other farmers

- Yes, always
- Yes, sometimes
- No
- Don't know

Use a vehicle dip/vehicle disinfection

- ◯ Yes, always
- ◯ Yes, sometimes
- ◯ No
- ◯ Don't know

8. Please complete the following table on your herd for the last 12 months

Nucleus hives

Disease/ Syndrome	Affected (%)	Mortality (%)
**Varroa**		
**American Foulbrood**		
**Breathing problems**		
**Deformed wings**		
**Diarrhea**		
**European Foulbrood**		
**Nosema**		

**Pesticide toxicity**		
**Other disease**		
**Unknown disease**		

Other Disease/ Syndrome, please specify

Production hives

**Disease/ Syndrome**	**Affected (%)**	**Mortality (%)**
**Varroa**		
**American Foulbrood**		
**Breathing problems**		
**Deformed wings**		
**Diarrhea**		
**European Foulbrood**		

**Nosema**		
**Pesticide toxicity**		
**Other disease**		
**Unknown disease**		

Other Disease/ Syndrome, please specify

8.1 Have you experienced an in increased incidence of any of these diseases since the lockdown due to COVID?

- ◯ Yes
- ◯ No

8.1.1 If yes, please specify

9. Do you use antibiotics (to end the survey if the answer is "No")

- ◯ Yes
- ◯ No

9.1 If yes, Do you use registered products

- ◯ Yes
- ◯ No
- ◯ Depends

If Depends, please specify

9.2 Do you treat the single affected hives or all the apiary?

- Single affected
- All the apiary
- Depends

If Depends, please specify

10. Please list maximum of 10 antibiotics you've used most frequently for treatment or prevention in the last 12 months including medicated feed

the 1st antibiotic

the 2nd antibiotic

the 3rd antibiotic

the 4th antibiotic

the 5th antibiotic

the 6th antibiotic

the 7th antibiotic

the 8th antibiotic

the 9th antibiotic

the 10th antibiotic

Please complete the following questions for the 1st Antibiotic

2. Reason for Antibiotic (from Disease/Syndrome)

- Varroa
- American Foulbrood
- Breathing problems
- Deformed wings
- Diarrhea
- Foulbrood
- Nosema
- Pesticide toxicity
- Other disease
- Unknown disease

Other, please specify

3. Stage of Life

- Nucleus hives
- Production hives

4. Aim

- Treatment
- Prevention

5. How

- Sugar water
- Powdered sugar

6. For how many days in a row on average?

7. What percentage of hives per year?

8. If this antibiotic doesn't work, what do you do?

- Repeat the treatment with the same antibiotic
- Consult with an expert
- Use a higher dose of the same antibiotic
- Use a different antibiotic
- Do nothing
- Eradicate hive
- Other

which one?

Do you test samples to identify the pathogen?

- Yes
- No

Do you wait for antibiotic susceptibility information from the laboratory?

- Yes
- No

Other, please specify

Please complete the following questions for the 2nd Antibiotic

2. Reason for Antibiotic (from Disease/Syndrome)

- Varroa
- American Foulbrood
- Breathing problems
- Deformed wings
- Diarrhea
- Foulbrood
- Nosema
- Pesticide toxicity
- Other disease
- Unknown disease

Other, please specify

3. Stage of Life

- Nucleus hives
- Production hives

4. Aim

- Treatment
- Prevention

5. How

- Sugar water
- Powdered sugar

6. For how many days in a row on average?

7. What percentage of hives per year?

8. If this antibiotic doesn't work, what do you do?

- Repeat the treatment with the same antibiotic
- Consult with an expert
- Use a higher dose of the same antibiotic
- Use a different antibiotic
- Do nothing
- Eradicate hive
- Other

which one?

Do you test samples to identify the pathogen?

- Yes
- No

Do you wait for antibiotic susceptibility information from the laboratory?

- Yes
- No

Other, please specify

Please complete the following questions for the 3rd Antibiotic

2. Reason for Antibiotic (from Disease/Syndrome)

- Varroa
- American Foulbrood
- Breathing problems
- Deformed wings
- Diarrhea
- Foulbrood
- Nosema
- Pesticide toxicity
- Other disease
- Unknown disease

Other, please specify

3. Stage of Life

- Nucleus hives
- Production hives

4. Aim

- Treatment
- Prevention

5. How

- Sugar water
- Powdered sugar

6. For how many days in a row on average?

7. What percentage of hives per year?

8. If this antibiotic doesn't work, what do you do?

- Repeat the treatment with the same antibiotic
- Consult with an expert
- Use a higher dose of the same antibiotic
- Use a different antibiotic
- Do nothing
- Eradicate hive
- Other

which one?

Do you test samples to identify the pathogen?

- Yes
- No

Do you wait for antibiotic susceptibility information from the laboratory?

- Yes
- No

Other, please specify

Please complete the following questions for the 4th Antibiotic

2. Reason for Antibiotic (from Disease/Syndrome)

- Varroa
- American Foulbrood
- Breathing problems
- Deformed wings
- Diarrhea
- Foulbrood
- Nosema
- Pesticide toxicity
- Other disease
- Unknown disease

Other, please specify

3. Stage of Life

- Nucleus hives
- Production hives

4. Aim

- Treatment
- Prevention

5. How

- Sugar water
- Powdered sugar

6. For how many days in a row on average?

7. What percentage of hives per year?

8. If this antibiotic doesn't work, what do you do?

- Repeat the treatment with the same antibiotic
- Consult with an expert
- Use a higher dose of the same antibiotic
- Use a different antibiotic
- Do nothing
- Eradicate hive
- Other

which one?

Do you test samples to identify the pathogen?

- Yes
- No

Do you wait for antibiotic susceptibility information from the laboratory?

- Yes
- No

Other, please specify

Please complete the following questions for the 5th Antibiotic

2. Reason for Antibiotic (from Disease/Syndrome)

- Varroa
- American Foulbrood
- Breathing problems
- Deformed wings
- Diarrhea
- Foulbrood
- Nosema
- Pesticide toxicity
- Other disease
- Unknown disease

Other, please specify

3. Stage of Life

- Nucleus hives
- Production hives

4. Aim

- Treatment
- Prevention

5. How

- Sugar water
- Powdered sugar

6. For how many days in a row on average?

7. What percentage of hives per year?

8. If this antibiotic doesn't work, what do you do?

- Repeat the treatment with the same antibiotic
- Consult with an expert
- Use a higher dose of the same antibiotic
- Use a different antibiotic
- Do nothing
- Eradicate hive
- Other

which one?

Do you test samples to identify the pathogen?

- Yes
- No

Do you wait for antibiotic susceptibility information from the laboratory?

- Yes
- No

Other, please specify

Please complete the following questions for the 6th Antibiotic

2. Reason for Antibiotic (from Disease/Syndrome)

- Varroa
- American Foulbrood
- Breathing problems
- Deformed wings
- Diarrhea
- Foulbrood
- Nosema
- Pesticide toxicity
- Other disease
- Unknown disease

Other, please specify

3. Stage of Life

- Nucleus hives
- Production hives

4. Aim

- Treatment
- Prevention

5. How

- Sugar water
- Powdered sugar

6. For how many days in a row on average?

7. What percentage of hives per year?

8. If this antibiotic doesn't work, what do you do?

- Repeat the treatment with the same antibiotic
- Consult with an expert
- Use a higher dose of the same antibiotic
- Use a different antibiotic
- Do nothing
- Eradicate hive
- Other

which one?

Do you test samples to identify the pathogen?

- Yes
- No

Do you wait for antibiotic susceptibility information from the laboratory?

- Yes
- No

Other, please specify

Please complete the following questions for the 7th Antibiotic

2. Reason for Antibiotic (from Disease/Syndrome)

- Varroa
- American Foulbrood
- Breathing problems
- Deformed wings
- Diarrhea
- Foulbrood
- Nosema
- Pesticide toxicity
- Other disease
- Unknown disease

Other, please specify

3. Stage of Life

- Nucleus hives
- Production hives

4. Aim

- Treatment
- Prevention

5. How

- Sugar water
- Powdered sugar

6. For how many days in a row on average?

7. What percentage of hives per year?

8. If this antibiotic doesn't work, what do you do?

- Repeat the treatment with the same antibiotic
- Consult with an expert
- Use a higher dose of the same antibiotic
- Use a different antibiotic
- Do nothing
- Eradicate hive
- Other

which one?

Do you test samples to identify the pathogen?

- Yes
- No

Do you wait for antibiotic susceptibility information from the laboratory?

- Yes
- No

Other, please specify

Please complete the following questions for the 8th Antibiotic

2. Reason for Antibiotic (from Disease/Syndrome)

- Varroa
- American Foulbrood
- Breathing problems
- Deformed wings
- Diarrhea
- Foulbrood
- Nosema
- Pesticide toxicity
- Other disease
- Unknown disease

Other, please specify

3. Stage of Life

- Nucleus hives
- Production hives

4. Aim

- Treatment
- Prevention

5. How

- Sugar water
- Powdered sugar

6. For how many days in a row on average?

7. What percentage of hives per year?

8. If this antibiotic doesn't work, what do you do?

- Repeat the treatment with the same antibiotic
- Consult with an expert
- Use a higher dose of the same antibiotic
- Use a different antibiotic
- Do nothing
- Eradicate hive
- Other

which one?

Do you test samples to identify the pathogen?

- Yes
- No

Do you wait for antibiotic susceptibility information from the laboratory?

- Yes
- No

Other, please specify

Please complete the following questions for the 9th Antibiotic

2. Reason for Antibiotic (from Disease/Syndrome)

- Varroa
- American Foulbrood
- Breathing problems
- Deformed wings
- Diarrhea
- Foulbrood
- Nosema
- Pesticide toxicity
- Other disease
- Unknown disease

Other, please specify

3. Stage of Life

- Nucleus hives
- Production hives

4. Aim

- Treatment
- Prevention

5. How

- Sugar water
- Powdered sugar

6. For how many days in a row on average?

7. What percentage of hives per year?

8. If this antibiotic doesn't work, what do you do?

- Repeat the treatment with the same antibiotic
- Consult with an expert
- Use a higher dose of the same antibiotic
- Use a different antibiotic
- Do nothing
- Eradicate hive
- Other

which one?

Do you test samples to identify the pathogen?

- Yes
- No

Do you wait for antibiotic susceptibility information from the laboratory?

- Yes
- No

Other, please specify

Please complete the following questions for the 10th Antibiotic

2. Reason for Antibiotic (from Disease/Syndrome)

- Varroa
- American Foulbrood
- Breathing problems
- Deformed wings
- Diarrhea
- Foulbrood
- Nosema
- Pesticide toxicity
- Other disease
- Unknown disease

Other, please specify

3. Stage of Life

- Nucleus hives
- Production hives

4. Aim

- Treatment
- Prevention

5. How

- Sugar water
- Powdered sugar

6. For how many days in a row on average?

7. What percentage of hives per year?

8. If this antibiotic doesn't work, what do you do?

- ◯ Repeat the treatment with the same antibiotic
- ◯ Consult with an expert
- ◯ Use a higher dose of the same antibiotic
- ◯ Use a different antibiotic
- ◯ Do nothing
- ◯ Eradicate hive
- ◯ Other

which one?

Do you test samples to identify the pathogen?

- ◯ Yes
- ◯ No

Do you wait for antibiotic susceptibility information from the laboratory?

- ◯ Yes
- ◯ No

Other, please specify

11. Did the participant refuse to answer any of these questions?

- ◯ Yes
- ◯ No

If yes, list the number(s) of these questions

If yes, check all reasons that participant gave for this refusal

- ☐ Concerned about researchers knowing this information
- ☐ Concerned about government knowing this information
- ☐ Concerned about other farmers knowing this information
- ☐ Other

Other, please specify
Comments

Backyard _Farmer Survey Part 2_FAO AMU Survey

Interviewer ID *

Interview Date *

yyyy-mm-dd

Farmer ID *

Choose the country *

- North Macedonia
- Bosnia and Herzegovina
- Serbia
- Albania
- Montenegro
- Kosovo

1. If you do any of the following when your animals/birds get sick, what order would you follow (e.g. 1st, 2nd, 3rd, etc.)	1st	2nd	3rd	4th	5th	6th
Treat with antibiotics	○	○	○	○	○	○
Isolate the sick animal(s)/chicken(s)	○	○	○	○	○	○
Call a veterinarian	○	○	○	○	○	○
Send samples to the diagnostic laboratory	○	○	○	○	○	○
Slaughter them for meat	○	○	○	○	○	○
Treat with non-antibiotic medications	○	○	○	○	○	○

Other	◯	◯	◯	◯	◯	◯

None of the above

◯ OK

Other, please specify

2. Estimate the percentage of your birds/animals that receive antibiotics at least once before they go to market (including medicated feed)

3. What percentage of your animals/birds died of disease over the last 12 months

**Type of animals**	**Percentage of chicks/young animals**	**Percentage of adult animals**
**Chickens for meat (broilers)**	Percentage of chicks	Percentage of adult birds
**Chickens for eggs (layers)**	Percentage of chicks	Percentage of adult birds
**Geese**	Percentage of chicks	Percentage of adult birds
**Ducks**	Percentage of chicks	Percentage of adult birds
**Turkeys**	Percentage of chicks	Percentage of adult birds
**Cattle for meat**	Percentage of young animals	Percentage of adult animals

**Cattle for milk**	Percentage of young animals	Percentage of adult animals
**Yaks**	Percentage of young animals	Percentage of adult animals
**Horses for meat**	Percentage of young animals	Percentage of adult animals
**Donkeys**	Percentage of young animals	Percentage of adult animals
**Sheep**	Percentage of young animals	Percentage of adult animals
**Goats**	Percentage of young animals	Percentage of adult animals
**Pigs**	Percentage of young animals	Percentage of adult animals
**Rabbits**	Percentage of young animals	Percentage of adult animals
**Bees**	Percentage of nucleus hives (Beginner hives) /Swarms	Percentage of production hives (colonies)
**Other**	Percentage of chicks/young animals	Percentage of adult animals

Specify if Other

4. Which of the following do you do on your farm (please check all that apply?

Buy birds/animals from the market

- Yes, always
- Yes, sometimes
- No
- Don't know

Keep new birds/animals separately for at least 30 days

- Yes, always
- Yes, sometimes
- No
- Don't know

Use shared/community pastures

- Yes, always
- Yes, sometimes
- No
- Don't know

Share breeding stock with other farmers, eg. Bulls, rams, boars, etc

- Yes, always
- Yes, sometimes
- No
- Don't know

Keep sick birds/animals separately

- Yes, always
- Yes, sometimes
- No
- Don't know

Share equipment with other farmers

- Yes, always
- Yes, sometimes
- No
- Don't know

Clean and disinfect equipment if shared with other farmers

- Yes, always
- Yes, sometimes
- No
- Don't know

Use a vehicle dip

- Yes, always
- Yes, sometimes
- No
- Don't know

Provide visitors with coveralls and boots

- Yes, always
- Yes, sometimes
- No
- Don't know

Have rodent and pest control measures in the barn(s)

- Yes, always
- Yes, sometimes
- No
- Don't know

Keep animals enclosed in pastures/pens

- Yes, always
- Yes, sometimes
- No
- Don't know

Keep birds enclosed in pastures/pens

- Yes, always
- Yes, sometimes
- No
- Don't know

Keep wild animals at least 30 m from your animals/birds

- Yes, always
- Yes, sometimes
- No
- Don't know

Prevent wild birds from accessing the feed storage

- Yes, always
- Yes, sometimes
- No
- Don't know

5. Please complete the following questions for any of these syndromes that you have seen or vaccinated for on your farm in the last 12 months

Type of animal and Disease/ Syndrome

1

Species of animals

- Chickens for meat (broilers)
- Chickens for eggs (layers)
- Geese
- Ducks
- Turkeys
- Cattle for meat
- Cattle for milk
- Horses for meat
- Sheep
- Goats
- Pigs
- Rabbits
- Bees
- Other

Other animal, please specify

Disease/ Syndrome

- Breathing problems
- Ear or Eye problems
- Intestinal problems
- Skin/ Feather problems
- Lameness
- Injuries
- Blindness
- Nervous system
- Reproductive problems
- Mastitis
- Not laying
- Other

Other Disease/ Syndrome, please specify

Young animals/birds

Affected (%)

Mortality (%)

Vaccinated (%)

Adults animals/birds

Affected (%)

Mortality (%)

Vaccinated (%)

5.1 Have you experienced an in increased incidence of any of these diseases since the lockdown due to COVID?

- ◯ Yes
- ◯ No

If yes, please specify

6. Do you use antibiotics to make healthy animals/birds grow faster/bigger or lay more eggs or give more milk even if the antibiotics are not needed to prevent or treat disease?

- ◯ Yes
- ◯ No

6.1 If yes complete the following questions

1

1.What Antibiotic (use numbered list provided)

2.Species of animal

- Chickens for meat (broilers)
- Chickens for eggs (layers)
- Geese
- Ducks
- Turkeys
- Cattle for meat
- Cattle for milk
- Horses for meat
- Sheep
- Goats
- Pigs
- Rabbits
- Bees
- Other

Other animal, please specify

3. Stages of Life

- Young
- Adult

4. How

- Feed
- Water

5. For how many days in a row on average?

6. What percentage of birds/animals per year?

7. Please complete the following table for the antibiotics you've used most frequently for treatment or prevention in the last 12 months (maximum of 5 antibiotics) including medicated feed

The farmer does not use any antibiotic (to end the survey)

- ○ OK

the 1st antibiotic

the 2nd antibiotic

the 3rd antibiotic

the 4th antibiotic

the 5th antibiotic

Please complete the following questions for the 1st Antibiotic

1. Reason for Antibiotic (from Disease/Syndrome)

- Breathing problems
- Ear or Eye problems
- Intestinal problems
- Skin/ Feather problems
- Lameness
- Injuries
- Blindness
- Nervous system
- Reproductive problems
- Mastitis
- Not laying
- Other

Other, please specify

2. Species of animal

- Chickens for meat (broilers)
- Chickens for eggs (layers)
- Geese
- Ducks
- Turkeys
- Cattle for meat
- Cattle for milk
- Horses for meat
- Sheep
- Goats
- Pigs
- Rabbits
- Bees
- other

Other, please specify

3. Stage of Life

- Young
- Adult

4. Aim

- Treatment
- Prevention

5. How

- Feed
- Water
- Injection
- Bolus
- Drench
- Topical

6. For how many days in a row on average?

7. What percentage of animals/birds per year?

8. If this antibiotic doesn't work, what do you do?

- Repeat the treatment with the same antibiotic
- Consult with a veterinarian
- Use a higher dose of the same antibiotic
- Use a different antibiotic
- Do nothing
- Send for slaughter
- Other

which one?

Other, please specify

Please complete the following questions for the 2nd Antibiotic

1. Reason for Antibiotic (from Disease/Syndrome)

- Breathing problems
- Ear or Eye problems
- Intestinal problems
- Skin/ Feather problems
- Lameness
- Injuries
- Blindness
- Nervous system
- Reproductive problems
- Mastitis
- Not laying
- Other

Other, please specify

2. Species of animal

- Chickens for meat (broilers)
- Chickens for eggs (layers)
- Geese
- Ducks
- Turkeys
- Cattle for meat
- Cattle for milk
- Horses for meat
- Sheep
- Goats
- Pigs
- Rabbits
- Bees
- other

Other, please specify

3. Stage of Life

- Young
- Adult

4. Aim

- Treatment
- Prevention

5. How

- Feed
- Water
- Injection
- Bolus
- Drench
- Topical

6. For how many days in a row on average?

7. What percentage of animals/birds per year?

8. If this antibiotic doesn't work, what do you do?

- Repeat the treatment with the same antibiotic
- Consult with a veterinarian
- Use a higher dose of the same antibiotic
- Use a different antibiotic
- Do nothing
- Send for slaughter
- Other

which one?

Other, please specify

Please complete the following questions for the 3rd Antibiotic

1. Reason for Antibiotic (from Disease/Syndrome)

- Breathing problems
- Ear or Eye problems
- Intestinal problems
- Skin/ Feather problems
- Lameness
- Injuries
- Blindness
- Nervous system
- Reproductive problems
- Mastitis
- Not laying
- Other

Other, please specify

2. Species of animal

- Chickens for meat (broilers)
- Chickens for eggs (layers)
- Geese
- Ducks
- Turkeys
- Cattle for meat
- Cattle for milk
- Horses for meat
- Sheep
- Goats
- Pigs
- Rabbits
- Bees
- other

Other, please specify

3. Stage of Life

- Young
- Adult

4. Aim

- Treatment
- Prevention

5. How

- Feed
- Water
- Injection
- Bolus
- Drench
- Topical

6. For how many days in a row on average?

7. What percentage of animals/birds per year?

8. If this antibiotic doesn't work, what do you do?

- Repeat the treatment with the same antibiotic
- Consult with a veterinarian
- Use a higher dose of the same antibiotic
- Use a different antibiotic
- Do nothing
- Send for slaughter
- Other

which one?

Other, please specify

Please complete the following questions for the 4th Antibiotic

1. Reason for Antibiotic (from Disease/Syndrome)

- Breathing problems
- Ear or Eye problems
- Intestinal problems
- Skin/ Feather problems
- Lameness
- Injuries
- Blindness
- Nervous system
- Reproductive problems
- Mastitis
- Not laying
- Other

Other, please specify

2. Species of animal

- Chickens for meat (broilers)
- Chickens for eggs (layers)
- Geese
- Ducks
- Turkeys
- Cattle for meat
- Cattle for milk
- Horses for meat
- Sheep
- Goats
- Pigs
- Rabbits
- Bees
- other

Other, please specify

3. Stage of Life

- Young
- Adult

4. Aim

- Treatment
- Prevention

5. How

- Feed
- Water
- Injection
- Bolus
- Drench
- Topical

6. For how many days in a row on average?

7. What percentage of animals/birds per year?

8. If this antibiotic doesn't work, what do you do?

- Repeat the treatment with the same antibiotic
- Consult with a veterinarian
- Use a higher dose of the same antibiotic
- Use a different antibiotic
- Do nothing
- Send for slaughter
- Other

which one?

Other, please specify

Please complete the following questions for the 5th Antibiotic

1. Reason for Antibiotic (from Disease/Syndrome)

- Breathing problems
- Ear or Eye problems
- Intestinal problems
- Skin/ Feather problems
- Lameness
- Injuries
- Blindness
- Nervous system
- Reproductive problems
- Mastitis
- Not laying
- Other

Other, please specify

2. Species of animal

- Chickens for meat (broilers)
- Chickens for eggs (layers)
- Geese
- Ducks
- Turkeys
- Cattle for meat
- Cattle for milk
- Horses for meat
- Sheep
- Goats
- Pigs
- Rabbits
- Bees
- other

Other, please specify

3. Stage of Life
- Young
- Adult

4. Aim
- Treatment
- Prevention

5. How
- Feed
- Water
- Injection
- Bolus
- Drench
- Topical

6. For how many days in a row on average?

7. What percentage of animals/birds per year?

8. If this antibiotic doesn't work, what do you do?
- Repeat the treatment with the same antibiotic
- Consult with a veterinarian
- Use a higher dose of the same antibiotic
- Use a different antibiotic
- Do nothing
- Send for slaughter
- Other

which one?

Other, please specify

8. Did the participant refuse to answer any of these questions?

- ◯ Yes
- ◯ No

If yes, list the number(s) of these questions

If yes, check all reasons that participant gave for this refusal

- ☐ Concerned about researchers knowing this information
- ☐ Concerned about government knowing this information
- ☐ Concerned about other farmers knowing this information
- ☐ Other

Other, please specify

Veterinarian Section _FAO AMU Survey

I. Has the informed consent form been explained, read by the participant and signed (please, accept)? *

- OK

II. Interviewer Identification code *

III. Date of Interview/Survey *

yyyy-mm-dd

VI. Veterinarian ID *

Please, collect the GPS point

latitude (x.y °)

longitude (x.y °)

altitude (m)

accuracy (m)

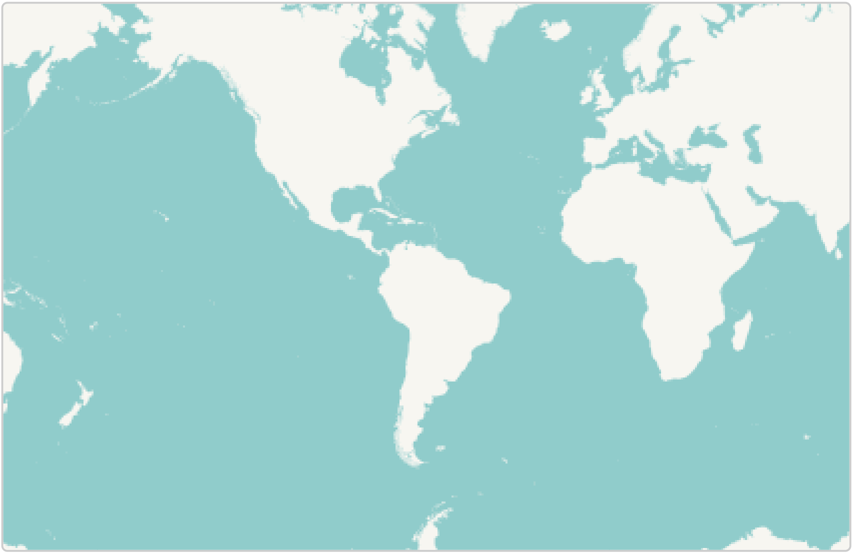

V. Choose the country *

- North Macedonia
- Bosnia and Herzegovina
- Serbia
- Albania
- Montenegro
- Kosovo

1. In what Region/Districts/Counties/Canton do you practice veterinary medicine in?

- [ ] Eastern
- [ ] Northeastern
- [ ] Pelagonia
- [ ] Polog
- [ ] Skopje
- [ ] Southeastern
- [ ] Southwestern
- [ ] Vardar

1. In what Region/Districts/Counties/Canton do you practice veterinary medicine in?

- [ ] Una-Sana
- [ ] Posavina
- [ ] Tuzla
- [ ] Zenica-Doboj
- [ ] Bosnian Podrinje
- [ ] Central Bosnia
- [ ] Herzegovina-Neretva
- [ ] West Herzegovina
- [ ] Sarajevo
- [ ] Canton 10
- [ ] Region Banja Luka
- [ ] Region Prijedor
- [ ] Region Doboj
- [ ] Region Bijeljina
- [ ] Region Istocno Sarajevo
- [ ] Region Trebinje

1. In what Region/Districts/Counties/Canton do you practice veterinary medicine in?

- [ ] Belgrade
- [ ] Vojvodina
- [ ] Šumadija and Western Serbia
- [ ] Southern and Eastern Serbia
- [ ] Kosovo and Metohija

1. In what Region/Districts/Counties/Canton do you practice veterinary medicine in?

- [ ] Tirana
- [ ] Elbasan
- [ ] Vlore
- [ ] Korce
- [ ] Dibra
- [ ] Kukes
- [ ] Durres
- [ ] Shkoder
- [ ] Fier
- [ ] Lezha
- [ ] Berat
- [ ] Gjirokastra

1. In what Region/Districts/Counties/Canton do you practice veterinary medicine in?

- [ ] Central Region
- [ ] Coastal Region
- [ ] Northern Region

1. In what Region/Districts/Counties/Canton do you practice veterinary medicine in?

- [ ] District of Ferizaj
- [ ] District of Gjakova
- [ ] District of Gjilan
- [ ] District of Mitrovica
- [ ] District of Peja
- [ ] District of Pristina
- [ ] District of Prizren

2. How many years have you been practicing as a veterinarian?

....

3. What is your age?

- ◯ <25 years old
- ◯ 25-40 years old
- ◯ 41-55 years old
- ◯ > 55 years old

4. What is your Gender?

- ◯ Male
- ◯ Female

5. Do you have income from other sources?

- ◯ Yes
- ◯ No

If yes, what other sources?

....

6. In this veterinary practice, are you:

....

The owner/ co-owner

- ◯ Yes
- ◯ No

The manager

- ◯ Yes
- ◯ No

An employee

- ◯ Yes
- ◯ No

Other

- ◯ Yes
- ◯ No

Specify if answer was "Other"

....

7. Was information on antibiotic resistance included in your veterinary education/training?

- Yes
- No

If yes, at what institution(s)?

8. What practice records do you have?

Number of farms in practice

- Yes
- No

Type of Livestock species in practice: e.g. Cattle, Horses

- Yes
- No

Size of farms in practice

- Yes
- No

Names of antibiotics sold/prescribed per year

- Yes
- No

Names of antibiotics sold/prescribed per farm

- Yes
- No

Names of antibiotics sold/prescribed per farm visit

- Yes
- No

Volume of each antibiotic sold/prescribed per year

- Yes
- No

Volume of each antibiotic sold/prescribed per farm

- Yes
- No

Volume of each antibiotic sold/prescribed per farm visit

- Yes
- No

Other

- Yes
- No

Other, please specify

9. Please indicate the number of each type of farm in your practice and the percentage of each type that is backyard, semi-commercial

Type of animals	Do you practice?	Total # farms	% of Backyard	% of Semi-commerical	% of Commercial
**Chickens for meat (broilers)**	Yes / No				
**Chickens for eggs (layers)**	Yes / No				
**Geese**	Yes / No				
**Ducks**	Yes / No				

**Turkeys**	Yes / No				
**Cattle for meat**	Yes / No				
**Cattle for milk**	Yes / No				
**Horses for meat**	Yes / No				
**Sheep**	Yes / No				
**Goats**	Yes / No				
**Pigs**	Yes / No				
**Rabbits**	Yes / No				
**Bees**	Yes / No				
**other**	Yes / No				

Other, please specify

10. Please rank the top 5 species of animals that are in your practice by the volume of antibiotic you prescribe for them, considering the type of farm

species of animals	rank the top 5 species of animals (1-5)	type of farm
**Chickens for meat (broilers)**		Backyard<br>Semi-commercial<br>Commercial
**Chickens for eggs (layers)**		Backyard<br>Semi-commercial<br>Commercial
**Geese**		Backyard<br>Semi-commercial<br>Commercial
**Ducks**		Backyard<br>Semi-commercial<br>Commercial
**Turkeys**		Backyard<br>Semi-commercial<br>Commercial
**Cattle for meat**		Backyard<br>Semi-commercial<br>Commercial

**Cattle for milk**		☐ Backyard<br>☐ Semi-commercial<br>☐ Commercial
**Horses for meat**		☐ Backyard<br>☐ Semi-commercial<br>☐ Commercial
**Sheep**		☐ Backyard<br>☐ Semi-commercial<br>☐ Commercial
**Goats**		☐ Backyard<br>☐ Semi-commercial<br>☐ Commercial
**Pigs**		☐ Backyard<br>☐ Semi-commercial<br>☐ Commercial
**Rabbits**		☐ Backyard<br>☐ Semi-commercial<br>☐ Commercial
**Bees**		☐ Backyard<br>☐ Semi-commercial<br>☐ Commercial
**Other**		☐ Backyard<br>☐ Semi-commercial<br>☐ Commercial

Other, please specify

11. When animal(s) needs antibiotic(s)

Do you write a prescription?

- Always
- Usually
- Rarely
- Never

Do you sell antibiotics to the farmer?

- Always
- Usually
- Rarely
- Never

Do your send a prescription to the feedmill for antibiotics given in feed?

- Always
- Usually
- Rarely
- Never

12. Where do you buy your antibiotics?

Veterinary pharmacy

- Always
- Usually
- Rarely
- Never

Human pharmacy

- Always
- Usually
- Rarely
- Never

Directly from outside of the country

- Always
- Usually
- Rarely
- Never

Wholesaler

- Always
- Usually
- Rarely
- Never

Market

- Always
- Usually
- Rarely
- Never

Other veterinarians

- Always
- Usually
- Rarely
- Never

Other

- Always
- Usually
- Rarely
- Never

Other, please specify

13. During or after the lockdown due to the COVID-19 situation, have you experienced any of the following?

I have had problems accessing antibiotics

- Yes
- No

I have had problems accessing disinfectants

- Yes
- No

I have had problems accessing vaccines

- Yes
- No

I have observed an increased mortality

- Yes
- No

I have had problems accessing farms

- Yes
- No

I had to increase the frequency of antibiotic treatments

- Yes
- No

I had to increase the dose of antibiotics

- Yes
- No

14. Do you combine individual antibiotics to create new products to prescribe/sell to your clients?

- Yes
- No

15. Why do you advise farmers to use antibiotics?

To help sick birds/animals get better

- ◯ Yes
- ◯ No

To prevent a healthy-looking birds/ animals from getting sick

- ◯ Yes
- ◯ No

To help the birds/ animals grow faster and bigger

- ◯ Yes
- ◯ No

other

- ◯ Yes
- ◯ No

Other, please specify

16. What diseases are the 5 most frequently diagnosed in your veterinary practice?

**Disease by species of animal**	**Disease**	**Species of animal**

**1st Disease**	◯ Breathing problems<br>◯ Ear or Eye problems<br>◯ Intestinal problems<br>◯ Skin/ Feather problems<br>◯ Lameness ◯ Injuries<br>◯ Blindness<br>◯ Nervous system<br>◯ Reproductive problems<br>◯ Mastitis ◯ Not laying<br>◯ Other reason	◯ Chickens for meat (broilers)<br>◯ Chickens for eggs (layers)<br>◯ Geese ◯ Ducks<br>◯ Turkeys ◯ Cattle for meat<br>◯ Cattle for milk<br>◯ Horses for meat ◯ Sheep<br>◯ Goats ◯ Pigs<br>◯ Rabbits ◯ Bees<br>◯ Other
**2nd Disease**	◯ Breathing problems<br>◯ Ear or Eye problems<br>◯ Intestinal problems<br>◯ Skin/ Feather problems<br>◯ Lameness ◯ Injuries<br>◯ Blindness<br>◯ Nervous system<br>◯ Reproductive problems<br>◯ Mastitis ◯ Not laying<br>◯ Other reason	◯ Chickens for meat (broilers)<br>◯ Chickens for eggs (layers)<br>◯ Geese ◯ Ducks<br>◯ Turkeys ◯ Cattle for meat<br>◯ Cattle for milk<br>◯ Horses for meat ◯ Sheep<br>◯ Goats ◯ Pigs<br>◯ Rabbits ◯ Bees<br>◯ Other
**3rd Disease**	◯ Breathing problems<br>◯ Ear or Eye problems<br>◯ Intestinal problems<br>◯ Skin/ Feather problems<br>◯ Lameness ◯ Injuries<br>◯ Blindness<br>◯ Nervous system<br>◯ Reproductive problems<br>◯ Mastitis ◯ Not laying<br>◯ Other reason	◯ Chickens for meat (broilers)<br>◯ Chickens for eggs (layers)<br>◯ Geese ◯ Ducks<br>◯ Turkeys ◯ Cattle for meat<br>◯ Cattle for milk<br>◯ Horses for meat ◯ Sheep<br>◯ Goats ◯ Pigs<br>◯ Rabbits ◯ Bees<br>◯ Other

**4th Disease**	○ Breathing problems<br>○ Ear or Eye problems<br>○ Intestinal problems<br>○ Skin/ Feather problems<br>○ Lameness ○ Injuries<br>○ Blindness<br>○ Nervous system<br>○ Reproductive problems<br>○ Mastitis ○ Not laying<br>○ Other reason	○ Chickens for meat (broilers)<br>○ Chickens for eggs (layers)<br>○ Geese ○ Ducks<br>○ Turkeys ○ Cattle for meat<br>○ Cattle for milk<br>○ Horses for meat ○ Sheep<br>○ Goats ○ Pigs<br>○ Rabbits ○ Bees<br>○ Other
**5th Disease**	○ Breathing problems<br>○ Ear or Eye problems<br>○ Intestinal problems<br>○ Skin/ Feather problems<br>○ Lameness ○ Injuries<br>○ Blindness<br>○ Nervous system<br>○ Reproductive problems<br>○ Mastitis ○ Not laying<br>○ Other reason	○ Chickens for meat (broilers)<br>○ Chickens for eggs (layers)<br>○ Geese ○ Ducks<br>○ Turkeys ○ Cattle for meat<br>○ Cattle for milk<br>○ Horses for meat ○ Sheep<br>○ Goats ○ Pigs<br>○ Rabbits ○ Bees<br>○ Other

Specify if Other Disease

Specify if Other Disease

Specify if Other Disease

Specify if Other Disease

Specify if Other Disease

Specify if Other Species of animal

Specify if Other Species of animal

Specify if Other Species of animal

Specify if Other Species of animal

Specify if Other Species of animal

17. What are the 5 diseases/syndromes that you most commonly advised your clients to use antibiotics for over the last 12 months

1st disease that you most commonly advised your clients to use antibiotics for over the last 12 months

Disease

- ◯ Breathing problems
- ◯ Ear or Eye problems
- ◯ Intestinal problems
- ◯ Skin/ Feather problems
- ◯ Lameness
- ◯ Injuries
- ◯ Blindness
- ◯ Nervous system
- ◯ Reproductive problems
- ◯ Mastitis
- ◯ Not laying
- ◯ Other reason

Specify if Other Disease

Species of animal

- Chickens for meat (broilers)
- Chickens for eggs (layers)
- Geese
- Ducks
- Turkeys
- Cattle for meat
- Cattle for milk
- Horses for meat
- Sheep
- Goats
- Pigs
- Rabbits
- Bees
- Other

Specify if Other Species of animal

Stage of Life

- Young
- Adult

Antibiotic used

Aim

- Treatment
- Prevention
- Growth Promotion

2nd disease that you most commonly advised your clients to use antibiotics for over the last 12 months

Disease

- ◯ Breathing problems
- ◯ Ear or Eye problems
- ◯ Intestinal problems
- ◯ Skin/ Feather problems
- ◯ Lameness
- ◯ Injuries
- ◯ Blindness
- ◯ Nervous system
- ◯ Reproductive problems
- ◯ Mastitis
- ◯ Not laying
- ◯ Other reason

Specify if Other Disease

Species of animal

- Chickens for meat (broilers)
- Chickens for eggs (layers)
- Geese
- Ducks
- Turkeys
- Cattle for meat
- Cattle for milk
- Horses for meat
- Sheep
- Goats
- Pigs
- Rabbits
- Bees
- Other

Specify if Other Species of animal

Stage of Life

- Young
- Adult

Antibiotic used

Aim

- Treatment
- Prevention
- Growth Promotion

3rd disease that you most commonly advised your clients to use antibiotics for over the last 12 months

Disease

- ◯ Breathing problems
- ◯ Ear or Eye problems
- ◯ Intestinal problems
- ◯ Skin/ Feather problems
- ◯ Lameness
- ◯ Injuries
- ◯ Blindness
- ◯ Nervous system
- ◯ Reproductive problems
- ◯ Mastitis
- ◯ Not laying
- ◯ Other reason

Specify if Other Disease

Species of animal

- Chickens for meat (broilers)
- Chickens for eggs (layers)
- Geese
- Ducks
- Turkeys
- Cattle for meat
- Cattle for milk
- Horses for meat
- Sheep
- Goats
- Pigs
- Rabbits
- Bees
- Other

Specify if Other Species of animal

Stage of Life

- Young
- Adult

Antibiotic used

Aim

- Treatment
- Prevention
- Growth Promotion

4th disease that you most commonly advised your clients to use antibiotics for over the last 12 months

Disease

- ◯ Breathing problems
- ◯ Ear or Eye problems
- ◯ Intestinal problems
- ◯ Skin/ Feather problems
- ◯ Lameness
- ◯ Injuries
- ◯ Blindness
- ◯ Nervous system
- ◯ Reproductive problems
- ◯ Mastitis
- ◯ Not laying
- ◯ Other reason

Specify if Other Disease

Species of animal

- Chickens for meat (broilers)
- Chickens for eggs (layers)
- Geese
- Ducks
- Turkeys
- Cattle for meat
- Cattle for milk
- Horses for meat
- Sheep
- Goats
- Pigs
- Rabbits
- Bees
- Other

Specify if Other Species of animal

Stage of Life

- Young
- Adult

Antibiotic used

Aim

- Treatment
- Prevention
- Growth Promotion

5th disease that you most commonly advised your clients to use antibiotics for over the last 12 months

Disease

- ◯ Breathing problems
- ◯ Ear or Eye problems
- ◯ Intestinal problems
- ◯ Skin/ Feather problems
- ◯ Lameness
- ◯ Injuries
- ◯ Blindness
- ◯ Nervous system
- ◯ Reproductive problems
- ◯ Mastitis
- ◯ Not laying
- ◯ Other reason

Specify if Other Disease

....

Species of animal

- Chickens for meat (broilers)
- Chickens for eggs (layers)
- Geese
- Ducks
- Turkeys
- Cattle for meat
- Cattle for milk
- Horses for meat
- Sheep
- Goats
- Pigs
- Rabbits
- Bees
- Other

Specify if Other Species of animal

Stage of Life

- Young
- Adult

Antibiotic used

Aim

- Treatment
- Prevention
- Growth Promotion

18. When a farm has sick animals that require antibiotics, do you advise the farmer to give antibiotics to:

Just the sick birds/ animals

- ◯ Always
- ◯ Usually
- ◯ Rarely
- ◯ Never

All of the birds/ animals in the pen

- ◯ Always
- ◯ Usually
- ◯ Rarely
- ◯ Never

All of the birds/ animals on the farm

- ◯ Always
- ◯ Usually
- ◯ Rarely
- ◯ Never

18.1 Would this advice be different for different animal species?

- ◯ Yes
- ◯ No

18.2 Would this advice be different for young animals vs adult animals?

- ◯ Yes
- ◯ No

19. Do you examine an animal(s) from a farm before you give advice on the use of antibiotics ?

- ◯ Always
- ◯ Usually
- ◯ Rarely
- ◯ Never

20. If you do not examine an animal from a farm before giving advice on the use of antibiotics, do you base that advice on

- [ ] Previous experience with that farm
- [ ] Previous experience with similar farms
- [ ] Experiences of other veterinarians
- [ ] The request of the farmer
- [ ] Other

Specify if Other

21. In your practice, for the issues listed below, are antibiotics?

Disease	Important to use	Commonly used
**Breathing problems/pneumonia**	◯ Yes ◯ No	◯ Yes ◯ No
**Lameness**	◯ Yes ◯ No	◯ Yes ◯ No
**Not eating/abnormal stools**	◯ Yes ◯ No	◯ Yes ◯ No
**Feather/ Skin problems**	◯ Yes ◯ No	◯ Yes ◯ No
**Nervous system**	◯ Yes ◯ No	◯ Yes ◯ No
**Improved production – growth/milk/eggs**	◯ Yes ◯ No	◯ Yes ◯ No

**Animals are to be stressed (weaned, surgery, grouped, transferred)**	Yes No	Yes No
**Reproductive problems**	Yes No	Yes No
**Mastitis**	Yes No	Yes No
**Other birds/animals are dying**	Yes No	Yes No
**Other**	Yes No	Yes No

Other, please specify

22. If you do any of the following when a farmer has sick birds/animals, what order would you follow (e.g. 1st, 2nd, 3rd, etc.)	1st	2nd	3rd	4th
Treat with antibiotics				
Isolate the sick bird(s)/animal(s)				
Send samples to the diagnostic laboratory				
Other				

Other, please specify

23. When would you submit samples to a diagnostic laboratory?

Single case of infectious disease in a regular client herd

- ◯ Yes
- ◯ No

Outbreak of infectious disease in a regular client herd

- ◯ Yes
- ◯ No

Single case of infectious disease in a herd where I have no previous information

- ◯ Yes
- ◯ No

Outbreak of disease in a herd where I have no previous information

- ◯ Yes
- ◯ No

Single case of infectious disease in certain food animal species

- ◯ Yes
- ◯ No

If yes, which species?

- ☐ Chickens for meat (broilers)
- ☐ Chickens for eggs (layers)
- ☐ Geese
- ☐ Ducks
- ☐ Turkeys
- ☐ Cattle for meat
- ☐ Cattle for milk
- ☐ Horses for meat
- ☐ Sheep
- ☐ Goats
- ☐ Pigs
- ☐ Rabbits
- ☐ Bees
- ☐ Other

Specify if Other Species of animal

Outbreak of disease in certain food animals species

- Yes
- No

If yes, which species?

- Chickens for meat (broilers)
- Chickens for eggs (layers)
- Geese
- Ducks
- Turkeys
- Cattle for meat
- Cattle for milk
- Horses for meat
- Sheep
- Goats
- Pigs
- Rabbits
- Bees
- Other

Specify if Other Species of animal

24. Please describe your access to a veterinary diagnostic laboratory:

My access is good, I have no issues with accessing diagnostic laboratory testing

- Yes
- No

I do not access the veterinary diagnostic laboratory because the laboratory is too far away

- Yes
- No

I do not access the veterinary diagnostic laboratory because my client does not want to pay for the testing

- ◯ Yes
- ◯ No

I do not access the veterinary diagnostic laboratory because the laboratory testing takes too long

- ◯ Yes
- ◯ No

I do not access the veterinary diagnostic laboratory because laboratory testing is not necessary

- ◯ Yes
- ◯ No

Other

- ◯ Yes
- ◯ No

Specify if Other

25. Is antibiotic susceptibility testing of bacteria available at your diagnostic laboratory?

- ◯ Yes, it is available for an additional cost
- ◯ No, it is not available
- ◯ I don't know

26. Would you change the prescribed treatment based on the antibiotic susceptibility testing results?

- ◯ Yes
- ◯ No

If no, why?

27. Do you use the results from previous antibiotic susceptibility results for future treatment decisions?

- ◯ Yes
- ◯ No

If no, why?

28. Do you believe that antibiotics are:

- As effective as they always were
- A little less effective
- Much less effective
- Effective if I use more than I used to
- I don't know

29. When you advise using antibiotics, do you provide information on withdrawal times for meat and/or milk?

- Always
- Usually
- Rarely
- Never

30. Did the participant refuse to answer any of these questions?

- Yes
- No

If yes, list the number(s) of these questions

If yes, check all reasons that participant gave for this refusal?

- Concerned about researchers knowing this information
- Concerned about government knowing this information
- Concerned about other Veterinarians knowing this information
- Other

Other, please specify

Comments

Pharmacy Section _FAO AMU Survey

I. Has the informed consent form been explained, read by the participant and signed (please, accept)? *

○ OK

II. Interviewer Identification code *

III. Date of Interview/Survey *

yyyy-mm-dd

IV. Veterinary Pharmacy ID *

Please, collect the GPS point

latitude (x.y °)

longitude (x.y °)

altitude (m)

accuracy (m)

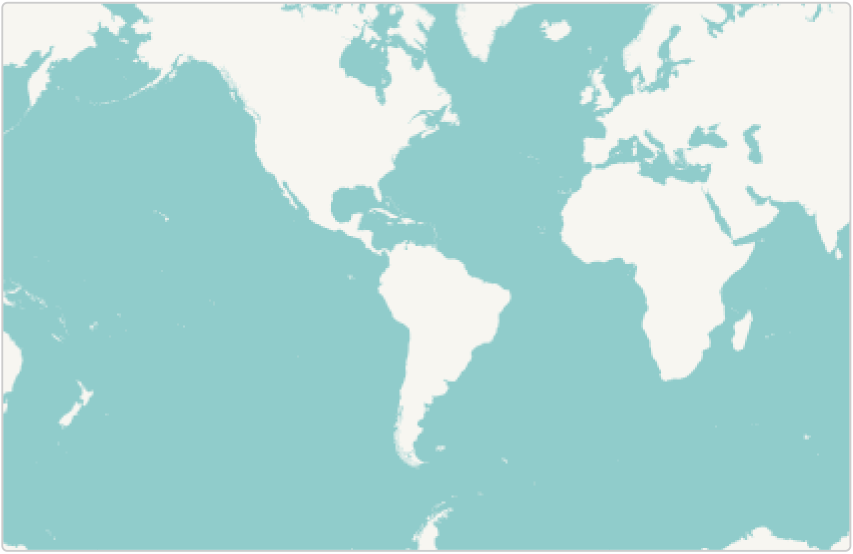

V. Choose the country *

○ North Macedonia
○ Bosnia and Herzegovina
○ Serbia
○ Albania
○ Montenegro
○ Kosovo

1. In whatRegion/Districts/Counties/Canton do you have clients?

- [ ] Eastern
- [ ] Northeastern
- [ ] Pelagonia
- [ ] Polog
- [ ] Skopje
- [ ] Southeastern
- [ ] Southwestern
- [ ] Vardar

1. In whatRegion/Districts/Counties/Canton do you have clients?

- [ ] Una-Sana
- [ ] Posavina
- [ ] Tuzla
- [ ] Zenica-Doboj
- [ ] Bosnian Podrinje
- [ ] Central Bosnia
- [ ] Herzegovina-Neretva
- [ ] West Herzegovina
- [ ] Sarajevo
- [ ] Canton 10
- [ ] Region Banja Luka
- [ ] Region Prijedor
- [ ] Region Doboj
- [ ] Region Bijeljina
- [ ] Region Istocno Sarajevo
- [ ] Region Trebinje

1. In whatRegion/Districts/Counties/Canton do you have clients?

- [ ] Belgrade
- [ ] Vojvodina
- [ ] Šumadija and Western Serbia
- [ ] Southern and Eastern Serbia
- [ ] Kosovo and Metohija

1. In whatRegion/Districts/Counties/Canton do you have clients?

- [ ] Tirana
- [ ] Elbasan
- [ ] Vlore
- [ ] Korce
- [ ] Dibra
- [ ] Kukes
- [ ] Durres
- [ ] Shkoder
- [ ] Fier
- [ ] Lezha
- [ ] Berat
- [ ] Gjirokastra

1. In whatRegion/Districts/Counties/Canton do you have clients?

- [ ] Central Region
- [ ] Coastal Region
- [ ] Northern Region

1. In whatRegion/Districts/Counties/Canton do you have clients?

- [ ] District of Ferizaj
- [ ] District of Gjakova
- [ ] District of Gjilan
- [ ] District of Mitrovica
- [ ] District of Peja
- [ ] District of Pristina
- [ ] District of Prizren

2. Are you a veterinary pharmacist?

- ◯ Yes
- ◯ No

2.1 If yes, how many years have you been a veterinary pharmacist?

....

3. Did you complete education or training in order to become a veterinary pharmacist?

- [ ] Yes, education
- [ ] Yes, training
- [ ] No

if education, please select what education?

- ○ High school
- ○ College education
- ○ Higher education/master degree
- ○ Other

Specify if answer was "Other"

....

if training, what training did you complete in order to become a veterinarian?

....

4. Was information on antibiotic resistance included in your training?

- ○ Yes
- ○ No

If yes, at what institution(s)?

....

5. What is your age?

- ○ <25 years old
- ○ 25-40 years old
- ○ 41-55 years old
- ○ > 55 years old

6. What is your Gender?

- ○ Male
- ○ Female

7. In this Veterinary Pharmacy, are you:

....

The owner/ co-owner

- Yes
- No

The manager

- Yes
- No

An employee

- Yes
- No

Other

- Yes
- No

Specify if answer was "Other"

8. What Pharmacy records do you have?

Number of clients

- Yes
- No

Names and addresses of clients

- Yes
- No

Species and production stage of animals that antibiotics are sold for

- Yes
- No

Number of animals that antibiotics are to be used in

- Yes
- No

Names of antibiotics sold per year

- Yes
- No

Names of antibiotics sold per client

- Yes
- No

Names of antibiotics sold per purchase

- Yes
- No

Volume of each antibiotic sold per year

- Yes
- No

Volume of each antibiotic sold per client

- Yes
- No

Volume of each antibiotic sold per purchase

- Yes
- No

Volume of each antibiotic sold per animal species

- Yes
- No

Value of each antibiotic sold per client

- Yes
- No

Value of each antibiotic sold per year

- Yes
- No

Other

- ◯ Yes
- ◯ No

Other, please specify

9. Where do you buy your medications/antibiotics?

Veterinary wholesaler

- ◯ Always
- ◯ Usually
- ◯ Rarely
- ◯ Never

Human pharmacy

- ◯ Always
- ◯ Usually
- ◯ Rarely
- ◯ Never

Directly from outside of your country

- ◯ Always
- ◯ Usually
- ◯ Rarely
- ◯ Never

Market

- ◯ Always
- ◯ Usually
- ◯ Rarely
- ◯ Never

Other pharmacists

- Always
- Usually
- Rarely
- Never

Other

- Always
- Usually
- Rarely
- Never

Other, please specify

10. Do you know what products containing antibiotics that you sell most frequently? (Hint: Example, trimethoprim-sulfamethazine oral powder, or tylosin premix, or Enroxil)

- Yes
- No

10.1 If yes, what are the 10 products containing antibiotics that you sell most frequently?

1st product containing antibiotics that you sell most frequently

Antibiotic

Species of animal

- Chickens for meat (broilers)
- Chickens for eggs (layers)
- Geese
- Ducks
- Turkeys
- Cattle for meat
- Cattle for milk
- Horses for meat
- Sheep
- Goats
- Pigs
- Rabbits
- Bees
- Other

Specify if Other Species of animal

Stage of life

- Growing animals
- Adult animals

Aim

- Treatment
- Prevention
- Growth Promotion

For which disease

- Breathing problems
- Ear or Eye problems
- Intestinal problems
- Skin/ Feather problems
- Lameness
- Injuries
- Blindness
- Nervous system
- Reproductive problems
- Mastitis
- Not laying
- Other

Specify if Other Disease

2nd product containing antibiotics that you sell most frequently

Antibiotic

Species of animal

- Chickens for meat (broilers)
- Chickens for eggs (layers)
- Geese
- Ducks
- Turkeys
- Cattle for meat
- Cattle for milk
- Horses for meat
- Sheep
- Goats
- Pigs
- Rabbits
- Bees
- Other

Specify if Other Species of animal

Stage of life

- Growing animals
- Adult animals

Aim

- Treatment
- Prevention
- Growth Promotion

For which disease

- Breathing problems
- Ear or Eye problems
- Intestinal problems
- Skin/ Feather problems
- Lameness
- Injuries
- Blindness
- Nervous system
- Reproductive problems
- Mastitis
- Not laying
- Other

Specify if Other Disease

3rd product containing antibiotics that you sell most frequently

Antibiotic

Species of animal

- Chickens for meat (broilers)
- Chickens for eggs (layers)
- Geese
- Ducks
- Turkeys
- Cattle for meat
- Cattle for milk
- Horses for meat
- Sheep
- Goats
- Pigs
- Rabbits
- Bees
- Other

Specify if Other Species of animal

Stage of life

- Growing animals
- Adult animals

Aim

- Treatment
- Prevention
- Growth Promotion

For which disease

- Breathing problems
- Ear or Eye problems
- Intestinal problems
- Skin/ Feather problems
- Lameness
- Injuries
- Blindness
- Nervous system
- Reproductive problems
- Mastitis
- Not laying
- Other

Specify if Other Disease

4th product containing antibiotics that you sell most frequently

Antibiotic

Species of animal

- Chickens for meat (broilers)
- Chickens for eggs (layers)
- Geese
- Ducks
- Turkeys
- Cattle for meat
- Cattle for milk
- Horses for meat
- Sheep
- Goats
- Pigs
- Rabbits
- Bees
- Other

Specify if Other Species of animal

Stage of life

- Growing animals
- Adult animals

Aim

- Treatment
- Prevention
- Growth Promotion

For which disease

- Breathing problems
- Ear or Eye problems
- Intestinal problems
- Skin/ Feather problems
- Lameness
- Injuries
- Blindness
- Nervous system
- Reproductive problems
- Mastitis
- Not laying
- Other

Specify if Other Disease

5th product containing antibiotics that you sell most frequently

Antibiotic

Species of animal

- Chickens for meat (broilers)
- Chickens for eggs (layers)
- Geese
- Ducks
- Turkeys
- Cattle for meat
- Cattle for milk
- Horses for meat
- Sheep
- Goats
- Pigs
- Rabbits
- Bees
- Other

Specify if Other Species of animal

Stage of life

- Growing animals
- Adult animals

Aim

- Treatment
- Prevention
- Growth Promotion

For which disease

- Breathing problems
- Ear or Eye problems
- Intestinal problems
- Skin/ Feather problems
- Lameness
- Injuries
- Blindness
- Nervous system
- Reproductive problems
- Mastitis
- Not laying
- Other

Specify if Other Disease

6th product containing antibiotics that you sell most frequently

Antibiotic

Species of animal

- Chickens for meat (broilers)
- Chickens for eggs (layers)
- Geese
- Ducks
- Turkeys
- Cattle for meat
- Cattle for milk
- Horses for meat
- Sheep
- Goats
- Pigs
- Rabbits
- Bees
- Other

Specify if Other Species of animal

Stage of life

- Growing animals
- Adult animals

Aim

- Treatment
- Prevention
- Growth Promotion

For which disease

- Breathing problems
- Ear or Eye problems
- Intestinal problems
- Skin/ Feather problems
- Lameness
- Injuries
- Blindness
- Nervous system
- Reproductive problems
- Mastitis
- Not laying
- Other

Specify if Other Disease

7th product containing antibiotics that you sell most frequently

Antibiotic

Species of animal

- Chickens for meat (broilers)
- Chickens for eggs (layers)
- Geese
- Ducks
- Turkeys
- Cattle for meat
- Cattle for milk
- Horses for meat
- Sheep
- Goats
- Pigs
- Rabbits
- Bees
- Other

Specify if Other Species of animal

Stage of life

- Growing animals
- Adult animals

Aim

- Treatment
- Prevention
- Growth Promotion

For which disease

- Breathing problems
- Ear or Eye problems
- Intestinal problems
- Skin/ Feather problems
- Lameness
- Injuries
- Blindness
- Nervous system
- Reproductive problems
- Mastitis
- Not laying
- Other

Specify if Other Disease

8th product containing antibiotics that you sell most frequently

Antibiotic

Species of animal

- Chickens for meat (broilers)
- Chickens for eggs (layers)
- Geese
- Ducks
- Turkeys
- Cattle for meat
- Cattle for milk
- Horses for meat
- Sheep
- Goats
- Pigs
- Rabbits
- Bees
- Other

Specify if Other Species of animal

Stage of life

- Growing animals
- Adult animals

Aim

- Treatment
- Prevention
- Growth Promotion

For which disease

- Breathing problems
- Ear or Eye problems
- Intestinal problems
- Skin/ Feather problems
- Lameness
- Injuries
- Blindness
- Nervous system
- Reproductive problems
- Mastitis
- Not laying
- Other

Specify if Other Disease

9th product containing antibiotics that you sell most frequently

Antibiotic

Species of animal

- Chickens for meat (broilers)
- Chickens for eggs (layers)
- Geese
- Ducks
- Turkeys
- Cattle for meat
- Cattle for milk
- Horses for meat
- Sheep
- Goats
- Pigs
- Rabbits
- Bees
- Other

Specify if Other Species of animal

Stage of life

- Growing animals
- Adult animals

Aim

- Treatment
- Prevention
- Growth Promotion

For which disease

- Breathing problems
- Ear or Eye problems
- Intestinal problems
- Skin/ Feather problems
- Lameness
- Injuries
- Blindness
- Nervous system
- Reproductive problems
- Mastitis
- Not laying
- Other

Specify if Other Disease

10th product containing antibiotics that you sell most frequently

Antibiotic

Species of animal

- Chickens for meat (broilers)
- Chickens for eggs (layers)
- Geese
- Ducks
- Turkeys
- Cattle for meat
- Cattle for milk
- Horses for meat
- Sheep
- Goats
- Pigs
- Rabbits
- Bees
- Other

Specify if Other Species of animal

Stage of life

- Growing animals
- Adult animals

Aim

- Treatment
- Prevention
- Growth Promotion

For which disease

- Breathing problems
- Ear or Eye problems
- Intestinal problems
- Skin/ Feather problems
- Lameness
- Injuries
- Blindness
- Nervous system
- Reproductive problems
- Mastitis
- Not laying
- Other

Specify if Other Disease

11. What percentage of your sales are to each of the following (they should add up to 100%)

Private veterinarians

Official veterinarians

Farmers/ public

Distributors

Feedmills

Unknown

Other

Specify if Other

12. Do you require a veterinary prescription to sell antibiotics to farmers directly?

- Always
- Usually
- Rarely
- Never

13. In your opinion, for these issues, are antibiotics?

Disease	Important to use	Commonly used
**Breathing problems/pneumonia**	Yes No Don't know	Yes No Don't know
**Lameness**	Yes No Don't know	Yes No Don't know
**Not eating/abnormal stools**	Yes No Don't know	Yes No Don't know
**Feather/ Skin problems**	Yes No Don't know	Yes No Don't know
**Nervous system**	Yes No Don't know	Yes No Don't know
**Improved production – growth/milk/eggs**	Yes No Don't know	Yes No Don't know

**Animals are to be stressed (weaned, surgery, grouped, transferred)**	Yes No Don't know	Yes No Don't know
**Reproductive problems**	Yes No Don't know	Yes No Don't know
**Mastitis**	Yes No Don't know	Yes No Don't know
**Other birds/animals are dying**	Yes No Don't know	Yes No Don't know
**Other**	Yes No Don't know	Yes No Don't know

Other, please specify

14. Do you believe that antimicrobials are:

- As effective as they always were
- A little less effective
- Much less effective
- Effective if I use more than I used to
- I don't know

15. Do you provide information on meat, eggs, or milk withdrawal information when you dispense antimicrobials?

- Yes
- No

If yes, where do you get this information?

16. During or after the lockdown due to the COVID-19 situation, have you experienced any of the following

I have had problems accessing antibiotics

- Yes
- No

I have had problems accessing disinfectants

- Yes
- No

I have had problems accessing vaccines

- Yes
- No

I was forced to sell expired antibiotics

- Yes
- No

17. Did the participant refuse to answer any of these questions?

- Yes
- No

If yes, list the number(s) of these questions

If yes, check all reasons that participant gave for this refusal?

- Concerned about researchers knowing this information
- Concerned about government knowing this information
- Concerned about other pharmacists knowing this information
- Other

Other, please specify

Comments

Feed Mill Section _FAO AMU Survey

I. Has the informed consent form been explained, read by the participant and signed (please, accept)? *

- ◯ OK

II. Interviewer Identification code *

III. Date of Interview/Survey *

yyyy-mm-dd

VI. Feed Mill ID *

Please, collect the GPS point

latitude (x.y °)

longitude (x.y °)

altitude (m)

accuracy (m)

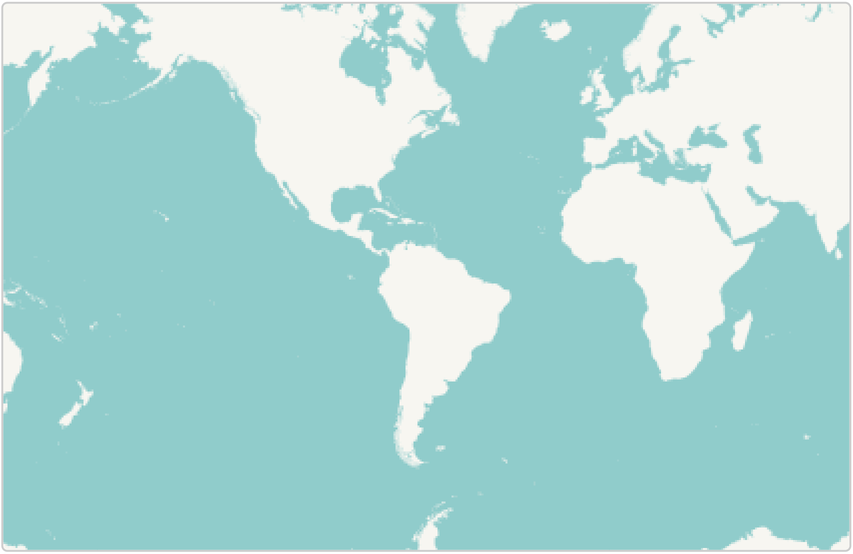

V. Choose the country *

- ◯ North Macedonia
- ◯ Bosnia and Herzegovina
- ◯ Serbia
- ◯ Albania
- ◯ Montenegro
- ◯ Kosovo

1. In what Region/Districts/Counties/Canton do you have clients?

- [ ] Eastern
- [ ] Northeastern
- [ ] Pelagonia
- [ ] Polog
- [ ] Skopje
- [ ] Southeastern
- [ ] Southwestern
- [ ] Vardar

1. In what Region/Districts/Counties/Canton do you have clients?

- [ ] Una-Sana
- [ ] Posavina
- [ ] Tuzla
- [ ] Zenica-Doboj
- [ ] Bosnian Podrinje
- [ ] Central Bosnia
- [ ] Herzegovina-Neretva
- [ ] West Herzegovina
- [ ] Sarajevo
- [ ] Canton 10
- [ ] Region Banja Luka
- [ ] Region Prijedor
- [ ] Region Doboj
- [ ] Region Bijeljina
- [ ] Region Istocno Sarajevo
- [ ] Region Trebinje

1. In what Region/Districts/Counties/Canton do you have clients?

- [ ] Belgrade
- [ ] Vojvodina
- [ ] Šumadija and Western Serbia
- [ ] Southern and Eastern Serbia
- [ ] Kosovo and Metohija

1. In what Region/Districts/Counties/Canton do you have clients?

- [ ] Tirana
- [ ] Elbasan
- [ ] Vlore
- [ ] Korce
- [ ] Dibra
- [ ] Kukes
- [ ] Durres
- [ ] Shkoder
- [ ] Fier
- [ ] Lezha
- [ ] Berat
- [ ] Gjirokastra

1. In what Region/Districts/Counties/Canton do you have clients?

- [ ] Central Region
- [ ] Coastal Region
- [ ] Northern Region

1. In what Region/Districts/Counties/Canton do you have clients?

- [ ] District of Ferizaj
- [ ] District of Gjakova
- [ ] District of Gjilan
- [ ] District of Mitrovica
- [ ] District of Peja
- [ ] District of Pristina
- [ ] District of Prizren

2. Did you complete education /training in order to work at the feed mill?

- [ ] Yes, education
- [ ] Yes, training
- [ ] No

if education, please select what education?

- High school
- College education
- Higher education/master degree
- Other

Specify if answer was "Other"

if training, what training did you complete in order to work at the feed mill?

3. Was information on antibiotic resistance included in your education /training?

- Yes
- No

If yes, at what institution(s)?

4. What is your age?

- <25 years old
- 25-40 years old
- 41-55 years old
- > 55 years old

5. What is your Gender?

- Male
- Female

6. In this Feed Mill, are you:

The owner/ co-owner

- Yes
- No

The manager

- Yes
- No

An employee

- Yes
- No

Other

- Yes
- No

Specify if answer was "Other"

7. Which of the following records do you have?

Number of clients

- Yes
- No

Names and addresses of clients

- Yes
- No

Type of animals that medicated feed is used in

- Yes
- No

Number of animals that medicated feed is fed to

- Yes
- No

Names of feed containing antibiotics sold per year

- Yes
- No

Names of feed containing antibiotics sold per client

- Yes
- No

Names of feed containing antibiotics sold per purchase

- Yes
- No

Volume of each feed containing antibiotics sold per year

- Yes
- No

Volume of each feed containing antibiotics sold per client

- Yes
- No

Volume of each feed containing antibiotics sold per purchase

- Yes
- No

Volume of each feed containing antibiotics sold per animal species

- Yes
- No

Total volume of feed sold per animal species

- Yes
- No

Value of medicated feed sales per antibiotic per client

- Yes
- No

Value of medicated feed sales per antibiotic per year

- Yes
- No

Other

- ◯ Yes
- ◯ No

Other, please specify

8. Where do you buy your medications/antibiotics?

Veterinary wholesaler

- ◯ Always
- ◯ Usually
- ◯ Rarely
- ◯ Never

Human pharmacy

- ◯ Always
- ◯ Usually
- ◯ Rarely
- ◯ Never

Directly from outside of your country

- ◯ Always
- ◯ Usually
- ◯ Rarely
- ◯ Never

Provided by Farmer

- ◯ Always
- ◯ Usually
- ◯ Rarely
- ◯ Never

Market

- Always
- Usually
- Rarely
- Never

Other feed mills

- Always
- Usually
- Rarely
- Never

Other

- Always
- Usually
- Rarely
- Never

Other, please specify

9. Do you require a prescription to sell medicated feed to a farmer?

- Always
- Usually
- Rarely
- Never

10. Do you keep medicated feed in inventory so it's available when requested?

- Always
- Usually
- Rarely
- Never

11. Which types of animals does your mill make feed for? Please indicate the approximate annual volume of feed sold and whether the feeds you make for each animal type include antibiotics automatically (A) or only when requested (R) or both (B).

Types of animals	Does the mill make feed for	Growing Animals	Growing Animals	Breeding/ Adult Animals	Breeding Adult animals
**Chickens for meat (broilers)**	Yes<br>No	Approximate annual volume of feed sold (tonnes)	The feeds you make include antibiotics<br>Automatically<br>Only when requested<br>Both	Approximate annual volume of feed sold (tonnes)	The feeds you make include antibiotics<br>Automatically<br>Only when requested<br>Both
**Chickens for eggs (layers)**	Yes<br>No	Approximate annual volume of feed sold (tonnes)	The feeds you make include antibiotics<br>Automatically<br>Only when requested<br>Both	Approximate annual volume of feed sold (tonnes)	The feeds you make include antibiotics<br>Automatically<br>Only when requested<br>Both
**Geese**	Yes<br>No	Approximate annual volume of feed sold (tonnes)	The feeds you make include antibiotics<br>Automatically<br>Only when requested<br>Both	Approximate annual volume of feed sold (tonnes)	The feeds you make include antibiotics<br>Automatically<br>Only when requested<br>Both

**Ducks**	◯ Yes<br>◯ No	Approximate annual volume of feed sold (tonnes)	The feeds you make include antibiotics<br>◯ Automatically<br>◯ Only when requested<br>◯ Both	Approximate annual volume of feed sold (tonnes)	The feeds you make include antibiotics<br>◯ Automatically<br>◯ Only when requested<br>◯ Both
**Turkeys**	◯ Yes<br>◯ No	Approximate annual volume of feed sold (tonnes)	The feeds you make include antibiotics<br>◯ Automatically<br>◯ Only when requested<br>◯ Both	Approximate annual volume of feed sold (tonnes)	The feeds you make include antibiotics<br>◯ Automatically<br>◯ Only when requested<br>◯ Both
**Cattle for meat**	◯ Yes<br>◯ No	Approximate annual volume of feed sold (tonnes)	The feeds you make include antibiotics<br>◯ Automatically<br>◯ Only when requested<br>◯ Both	Approximate annual volume of feed sold (tonnes)	The feeds you make include antibiotics<br>◯ Automatically<br>◯ Only when requested<br>◯ Both
**Cattle for milk**	◯ Yes<br>◯ No	Approximate annual volume of feed sold (tonnes)	The feeds you make include antibiotics<br>◯ Automatically<br>◯ Only when requested<br>◯ Both	Approximate annual volume of feed sold (tonnes)	The feeds you make include antibiotics<br>◯ Automatically<br>◯ Only when requested<br>◯ Both

**Horses for meat**	◯ Yes<br>◯ No	Approximate annual volume of feed sold (tonnes)	The feeds you make include antibiotics<br>◯ Automatically<br>◯ Only when requested<br>◯ Both	Approximate annual volume of feed sold (tonnes)	The feeds you make include antibiotics<br>◯ Automatically<br>◯ Only when requested<br>◯ Both
**Horses for milk**	◯ Yes<br>◯ No	Approximate annual volume of feed sold (tonnes)	The feeds you make include antibiotics<br>◯ Automatically<br>◯ Only when requested<br>◯ Both	Approximate annual volume of feed sold (tonnes)	The feeds you make include antibiotics<br>◯ Automatically<br>◯ Only when requested<br>◯ Both
**Sheep**	◯ Yes<br>◯ No	Approximate annual volume of feed sold (tonnes)	The feeds you make include antibiotics<br>◯ Automatically<br>◯ Only when requested<br>◯ Both	Approximate annual volume of feed sold (tonnes)	The feeds you make include antibiotics<br>◯ Automatically<br>◯ Only when requested<br>◯ Both
**Goats**	◯ Yes<br>◯ No	Approximate annual volume of feed sold (tonnes)	The feeds you make include antibiotics<br>◯ Automatically<br>◯ Only when requested<br>◯ Both	Approximate annual volume of feed sold (tonnes)	The feeds you make include antibiotics<br>◯ Automatically<br>◯ Only when requested<br>◯ Both

**Pigs**	◯ Yes<br>◯ No	Approximate annual volume of feed sold (tonnes)	The feeds you make include antibiotics<br>◯ Automatically<br>◯ Only when requested<br>◯ Both	Approximate annual volume of feed sold (tonnes)	The feeds you make include antibiotics<br>◯ Automatically<br>◯ Only when requested<br>◯ Both
**Rabbits**	◯ Yes<br>◯ No	Approximate annual volume of feed sold (tonnes)	The feeds you make include antibiotics<br>◯ Automatically<br>◯ Only when requested<br>◯ Both	Approximate annual volume of feed sold (tonnes)	The feeds you make include antibiotics<br>◯ Automatically<br>◯ Only when requested<br>◯ Both
**Bees**	◯ Yes<br>◯ No	Nucleus hives (Beginner hives) /Swarms/ approximate annual volume of feed sold (tonnes)	Nucleus hives (Beginner hives) /Swarms/: the feeds you make include antibiotics<br>◯ Automatically<br>◯ Only when requested<br>◯ Both	Production hives (colonies)/ aproximate annual volume of feed sold (tonnes)	Production hives (colonies)/: Antibiotics in feed<br>◯ Automatically<br>◯ Only when requested<br>◯ Both

**Other**	◯ Yes<br>◯ No	Approximate annual volume of feed sold (tonnes)	The feeds you make include antibiotics<br>◯ Automatically<br>◯ Only when requested<br>◯ Both	Approximate annual volume of feed sold (tonnes)	The feeds you make include antibiotics<br>◯ Automatically<br>◯ Only when requested<br>◯ Both

Other, please specify

12. Do you know the antibiotics that you most commonly include in medicated feed? (Select one)

◯ Yes

◯ No

12.1 If yes, what are the antibiotics that you most commonly include in medicated feed?

1st antibiotic that you most commonly include in medicated feed?

Antibiotic

Species of animal

- Chickens for meat (broilers)
- Chickens for eggs (layers)
- Geese
- Ducks
- Turkeys
- Cattle for meat
- Cattle for milk
- Horses for meat
- Horses for milk
- Sheep
- Goats
- Pigs
- Rabbits
- Bees
- Other

Specify if Other Species of animal

Aim

- Treatment
- Prevention
- Growth Promotion

For which disease

- Breathing problems
- Ear or Eye problems
- Intestinal problems
- Skin/ Feather problems
- Lameness
- Injuries
- Blindness
- Nervous system
- Reproductive problems
- Mastitis
- Not laying
- Other

Specify if Other Disease

2nd antibiotic that you most commonly include in medicated feed?

Antibiotic

Species of animal

- Chickens for meat (broilers)
- Chickens for eggs (layers)
- Geese
- Ducks
- Turkeys
- Cattle for meat
- Cattle for milk
- Horses for meat
- Horses for milk
- Sheep
- Goats
- Pigs
- Rabbits
- Bees
- Other

Specify if Other Species of animal

Aim

- Treatment
- Prevention
- Growth Promotion

For which disease

- Breathing problems
- Ear or Eye problems
- Intestinal problems
- Skin/ Feather problems
- Lameness
- Injuries
- Blindness
- Nervous system
- Reproductive problems
- Mastitis
- Not laying
- Other

Specify if Other Disease

3rd antibiotic that you most commonly include in medicated feed?

Antibiotic

Species of animal

- Chickens for meat (broilers)
- Chickens for eggs (layers)
- Geese
- Ducks
- Turkeys
- Cattle for meat
- Cattle for milk
- Horses for meat
- Horses for milk
- Sheep
- Goats
- Pigs
- Rabbits
- Bees
- Other

Specify if Other Species of animal

Aim

- Treatment
- Prevention
- Growth Promotion

For which disease

- Breathing problems
- Ear or Eye problems
- Intestinal problems
- Skin/ Feather problems
- Lameness
- Injuries
- Blindness
- Nervous system
- Reproductive problems
- Mastitis
- Not laying
- Other

Specify if Other Disease

4th antibiotic that you most commonly include in medicated feed?

Antibiotic

Species of animal

- Chickens for meat (broilers)
- Chickens for eggs (layers)
- Geese
- Ducks
- Turkeys
- Cattle for meat
- Cattle for milk
- Horses for meat
- Horses for milk
- Sheep
- Goats
- Pigs
- Rabbits
- Bees
- Other

Specify if Other Species of animal

Aim

- Treatment
- Prevention
- Growth Promotion

For which disease

- Breathing problems
- Ear or Eye problems
- Intestinal problems
- Skin/ Feather problems
- Lameness
- Injuries
- Blindness
- Nervous system
- Reproductive problems
- Mastitis
- Not laying
- Other

Specify if Other Disease

5th antibiotic that you most commonly include in medicated feed?

Antibiotic

Species of animal

- Chickens for meat (broilers)
- Chickens for eggs (layers)
- Geese
- Ducks
- Turkeys
- Cattle for meat
- Cattle for milk
- Horses for meat
- Horses for milk
- Sheep
- Goats
- Pigs
- Rabbits
- Bees
- Other

Specify if Other Species of animal

Aim

- Treatment
- Prevention
- Growth Promotion

For which disease

- Breathing problems
- Ear or Eye problems
- Intestinal problems
- Skin/ Feather problems
- Lameness
- Injuries
- Blindness
- Nervous system
- Reproductive problems
- Mastitis
- Not laying
- Other

Specify if Other Disease

6th antibiotic that you most commonly include in medicated feed?

Antibiotic

Species of animal

- Chickens for meat (broilers)
- Chickens for eggs (layers)
- Geese
- Ducks
- Turkeys
- Cattle for meat
- Cattle for milk
- Horses for meat
- Horses for milk
- Sheep
- Goats
- Pigs
- Rabbits
- Bees
- Other

Specify if Other Species of animal

Aim

- Treatment
- Prevention
- Growth Promotion

For which disease

- Breathing problems
- Ear or Eye problems
- Intestinal problems
- Skin/ Feather problems
- Lameness
- Injuries
- Blindness
- Nervous system
- Reproductive problems
- Mastitis
- Not laying
- Other

Specify if Other Disease

7th antibiotic that you most commonly include in medicated feed?

Antibiotic

Species of animal

- Chickens for meat (broilers)
- Chickens for eggs (layers)
- Geese
- Ducks
- Turkeys
- Cattle for meat
- Cattle for milk
- Horses for meat
- Horses for milk
- Sheep
- Goats
- Pigs
- Rabbits
- Bees
- Other

Specify if Other Species of animal

Aim

- Treatment
- Prevention
- Growth Promotion

For which disease

- Breathing problems
- Ear or Eye problems
- Intestinal problems
- Skin/ Feather problems
- Lameness
- Injuries
- Blindness
- Nervous system
- Reproductive problems
- Mastitis
- Not laying
- Other

Specify if Other Disease

8th antibiotic that you most commonly include in medicated feed?

Antibiotic

Species of animal

- Chickens for meat (broilers)
- Chickens for eggs (layers)
- Geese
- Ducks
- Turkeys
- Cattle for meat
- Cattle for milk
- Horses for meat
- Horses for milk
- Sheep
- Goats
- Pigs
- Rabbits
- Bees
- Other

Specify if Other Species of animal

Aim

- Treatment
- Prevention
- Growth Promotion

For which disease

- Breathing problems
- Ear or Eye problems
- Intestinal problems
- Skin/ Feather problems
- Lameness
- Injuries
- Blindness
- Nervous system
- Reproductive problems
- Mastitis
- Not laying
- Other

Specify if Other Disease

9th antibiotic that you most commonly include in medicated feed?

Antibiotic

Species of animal

- Chickens for meat (broilers)
- Chickens for eggs (layers)
- Geese
- Ducks
- Turkeys
- Cattle for meat
- Cattle for milk
- Horses for meat
- Horses for milk
- Sheep
- Goats
- Pigs
- Rabbits
- Bees
- Other

Specify if Other Species of animal

Aim

- Treatment
- Prevention
- Growth Promotion

For which disease

- Breathing problems
- Ear or Eye problems
- Intestinal problems
- Skin/ Feather problems
- Lameness
- Injuries
- Blindness
- Nervous system
- Reproductive problems
- Mastitis
- Not laying
- Other

Specify if Other Disease

10th antibiotic that you most commonly include in medicated feed?

Antibiotic

Species of animal

- Chickens for meat (broilers)
- Chickens for eggs (layers)
- Geese
- Ducks
- Turkeys
- Cattle for meat
- Cattle for milk
- Horses for meat
- Horses for milk
- Sheep
- Goats
- Pigs
- Rabbits
- Bees
- Other

Specify if Other Species of animal

Aim

- Treatment
- Prevention
- Growth Promotion

For which disease

- Breathing problems
- Ear or Eye problems
- Intestinal problems
- Skin/ Feather problems
- Lameness
- Injuries
- Blindness
- Nervous system
- Reproductive problems
- Mastitis
- Not laying
- Other

Specify if Other Disease

13. Do you believe that antibiotics are:

- As effective as they always were
- A little less effective
- Much less effective
- Effective if I use more than I used to
- I don't know

14. Do you provide information on meat or milk withdrawal information when you sell medicated feed?

- Yes
- No

If yes, where do you obtain this information?

15. During or after the lockdown due to the COVID-19 situation, have you experienced any of the following

I have had problems accessing antibiotics

- Yes
- No

I was forced to sell expired antibiotics

- Yes
- No

I have had problems accessing farms

- Yes
- No

16. Did the participant refuse to answer any of these questions?

- Yes
- No

If yes, list the number(s) of these questions

If yes, check all reasons that participant gave for this refusal?

- Concerned about researchers knowing this information
- Concerned about government knowing this information
- Concerned about other feed mills knowing this information
- Other

Other, please specify

Comments
